# The sine-Gordon equation in light-cone coordinates on the half-lines revisited: a Riemann–Hilbert approach

**DOI:** 10.1007/s11005-026-02147-8

**Published:** 2026-09-03

**Authors:** Iryna Karpenko

**Affiliations:** 1 Faculty of Mathematics, University of Vienna, Oskar-Morgenstern-Platz 1, 1090 Vienna, Austria; 2 B. Verkin Institute for Low Temperature Physics and Engineering, 47, Nauky Ave, Kharkiv, 61103 Ukraine

**Keywords:** Riemann–Hilbert problem, Fokas method, Initial-boundary value problem, Sine-Gordon equation, 35Q55, 35Q15, 37K15

## Abstract

In this work, we study the initial-boundary value (IBV) problems for the sine-Gordon (sG) equation in the light-cone coordinates $u_{xt}=\sin u$ in the quarter-planes x>0,t>0 and x<0,t>0, assuming a suitable decay as $x\rightarrow +\infty$ or as $x\rightarrow -\infty$. Employing the Riemann–Hilbert (RH) problem framework, we demonstrate that these two IBV problems differ significantly with respect to the boundary data required for well-posedness. Specifically, the solution of the “right problem” (x≥0) is uniquely determined by the initial data *u*(*x*, 0), x≥0 alone, whereas for the “left problem” (x≤0), the boundary data *u*(0, *t*) have to be prescribed in addition to the initial data in order to obtain a well-posed problem. The latter problem is solved using the unified transform method (also known as the Fokas method).

## Introduction

The sine-Gordon (sG) equation is one of the classical integrable nonlinear equations and has been extensively studied. In the laboratory coordinates (y,τ) it takes the form

$$ u_{yy}-u_{\tau \tau }+\sin u=0. $$

The sG equation arises in differential geometry in the theory of surfaces of constant negative curvature and appears in several areas of physics, including the theory of crystal dislocations and the dynamics of Josephson junctions (see, e.g., [1–4]).

Introducing the light-cone variables

$$ x=\frac{1}{2}(y+\tau ),\quad t=\frac{1}{2}(y-\tau ) $$

the equation transforms into

1.1 $$\begin{aligned} u_{xt} = \sin u. \end{aligned}$$

In these variables, *x* and *t* correspond to the characteristic directions of a hyperbolic equation.

The Cauchy problem on the whole line for the sG equation was solved using the inverse scattering transform by Ablowitz, Kaup, Newell, and Segur (see [5, 6]). This method reveals the rich structure of the sG equation, including the existence of localized solutions such as kinks and breathers, which correspond to discrete eigenvalues of the associated spectral problem (see [6]).

The analysis becomes considerably more complicated when the sG equation is posed in domains with boundaries, such as quarter-planes and semi-strips. To address such problems, Fokas introduced a general and systematic framework known as the unified transform method (UTM), widely referred to as the Fokas method (see [7]), which has subsequently been developed and extended by many authors [8–13]. The method is based on the simultaneous spectral analysis of both equations of the associated Lax pair.

In [14], Fokas and Its studied the sG equation in laboratory coordinates in the quarter-plane with prescribed initial data and boundary values (see also [7, 15]). In a later work [16], the equation $u_{xt}=-\sin u$ was considered on a finite spatial interval (0, *l*). In both formulations, the initial profile and the boundary values are assumed to be prescribed as part of the data of the problem.

An inverse spectral approach was used by Sakhnovich, who investigated the sG equation in semi-strip domains using the Weyl function associated with an auxiliary Dirac-type system arising from the Lax pair (see [17, 18]). In particular, this approach makes it possible to study the evolution of the corresponding spectral data and to describe situations in which solutions become unbounded in the quarter-plane. In [19], the Dirichlet problem on the half-line is considered with prescribed initial data *u*(*x*, 0) and boundary data *u*(0, *t*). These data are required to satisfy appropriate compatibility conditions, and the solution is reconstructed from the corresponding scattering data.

A different inverse spectral approach to the Dirichlet problem on the half-line was proposed by Leon [20]. In this work, the sG equation in light-cone coordinates (in the form $u_{xt}=-\sin u$, which can be related to (1.1) by the change of variable x↦-x) is analyzed using an alternative Lax pair associated with a Schrödinger-type spectral problem on the half-line.

Despite the extensive literature on initial-boundary value (IBV) problems for the sine-Gordon equation, several questions concerning the well-posedness of these problems have remained open, particularly regarding the decay conditions as $|x|\rightarrow \infty$. In the present paper, we address these questions by studying two initial-boundary value problems for the sG equation in the light-cone coordinates (1.1), posed on the half-lines x≥0 (Problem I) and x≤0 (Problem II), building on the Fokas method for half-line problems.

In what follows, we will use standard functional spaces, with $\Omega =(0,\infty )$ or $\Omega =(-\infty ,0)$, depending on the context:

$$\begin{aligned}&H^{2,2}(\Omega ):=\{f(x)\in L^2(\Omega ):~ x^2f(x), f'(x), x^2f'(x),f''(x), x^2f''(x) \in L^2(\Omega )\},\\&C([0,T],X) := \left\{ u:[0,T]\rightarrow X: u \text { is continuous on } [0,T] \text { with values in } X \right\} ,\\&C^{k}([0,T],X) := \{ u:[0,T]\rightarrow X: \partial _t^{,j}u\in C([0,T],X), \quad j=0,1,\ldots ,k \}, \end{aligned}$$

and

$$ \tilde{H}^{1,2}(\Omega ):=\{f(x)\in L^1_{loc}(\Omega ): (1+x^2)\sin \frac{f(x)}{2},(1+x^2)f_x(x)\in L^2(\Omega )\} $$

with a pseudometric

$$ d_{\tilde{H}^{1,2}}(f,g):= \left( \left\| (1+x^2)\sin \!\left( \frac{f-g}{2}\right) \right\| _{L^2(\Omega )}^2 + \left\| (1+x^2)(f_x-g_x)\right\| _{L^2(\Omega )}^2 \right) ^{\frac{1}{2}}. $$

For Problem I, we assume that the initial condition $u(x,0) = u_0(x)$ for x≥0 is given and $u_0(x)\in H^{2,2}((0,+\infty ))+2\pi k$. We consider the following problem

1.2 $$\begin{aligned} \begin{aligned}&u_{xt} = \sin u,\quad x\ge 0,\quad 0\le t<T<\infty ,\\&u(x,0) = u_0(x), \quad x \ge 0 \end{aligned} \end{aligned}$$

and seek solutions *u*(*x*, *t*) such that $u\in C^1([0,T],\tilde{H}^{1,2}(0,\infty ))$.

For Problem II, we assume that the initial condition $u(x,0) = u_0(x)\in H^{2,2}((-\infty ,0))+2\pi k$ for x≤0, and the boundary condition $u(0,t)=v_0(t)\in H^{1}(0,T)$ are given. We consider the following IBV problem

1.3 $$\begin{aligned} \begin{aligned}&u_{xt} = \sin u,\quad x\le 0,\quad 0\le t<T<\infty ,\\&u(x,0) = u_0(x), \quad x \le 0,\\&u(0,t)=v_0(t),\qquad t\in [0,T] \end{aligned} \end{aligned}$$

and seek solutions *u*(*x*, *t*) such that $u\in C^1([0,T],\tilde{H}^{1,2}((-\infty ,0)))$.

The conditions imposed on the behavior of solutions of the IBV problems at the spatial infinity affect the well-posedness of half-line problems crucially. A striking illustration is provided by Sakhnovich’s treatment of the sine-Gordon equation on the quarter-plane x≥0, t≥0 [18]. Under suitable assumptions on the boundary data, assuming also that the spatial derivative of the solution is bounded in the whole quarter-plane, the boundary data determine uniquely the initial Weyl function and thus the initial values; see [18, Corollary 4.10]. In particular, zero boundary data force the initial Weyl function, and therefore the corresponding initial data, to be trivial; see [18, Example 4.11].

The outline of the paper is as follows.

In Sect. 2, we present the basic tools for our analysis derived from the Lax pair representation of the sG equation, namely the Jost solutions (“eigenfunctions”) and the associated spectral functions (scattering coefficients).

In Sect. 3, we discuss the compatibility conditions for the initial and boundary values.

In Sect. 4, we describe the inverse spectral problems for the initial and boundary values in terms of solutions of the associated RH problems.

In Sect. 5, the master RH problems are constructed, whose solutions provide the solutions to Problem I and Problem II.

In Sect. 6, we show how a local solution of the sG equation can be constructed from the solution of a RH problem parametrized by *x* and *t*.

In Sect. 7, the differentiability of the solution of the RH problem is discussed.

In Sect. 8, we address Problem I, for which we find that the initial data alone uniquely determine its solution.

In Sect. 9, we focus on Problem II, where we show that in addition to the initial data, the boundary value *u*(0, *t*) should be prescribed to uniquely determine the solution.

The main results of the paper are formulated in Theorems 8.1, 8.3, 8.8, 9.1, and 9.3.

### Notations

In what follows, $$\sigma _1:=\left( {\begin{smallmatrix}0& 1\\ 1& 0\end{smallmatrix}}\right) $$, $$\sigma _2:=\left( {\begin{smallmatrix}0& -\textrm{i}\\ \textrm{i}& 0\end{smallmatrix}}\right) $$, and $$\sigma _3:=\left( {\begin{smallmatrix}1& 0\\ 0& -1\end{smallmatrix}}\right) $$ denote the standard Pauli matrices, $e^{\hat{\sigma }_3}A:=e^{\sigma _3}A e^{-\sigma _3}$, $\mathbb {C}^+:=\{\lambda \in \mathbb {C}|\operatorname {Im}(\lambda )> 0\}$, and $\mathbb {C}^-:=\{\lambda \in \mathbb {C}|\operatorname {Im}(\lambda )< 0\}$. We also let $f^*(k):=\overline{f(\bar{k})}$ denote the Schwarz reflection of a function *f*(*k*), $k\in \mathbb {C}$.

## Eigenfunctions and spectral functions

The sine-Gordon equation (1.1) is the compatibility condition for the Lax pair

2.1a $$\begin{aligned} \tilde{\Phi }_x&=U\tilde{\Phi }, \end{aligned}$$

2.1b $$\begin{aligned} \tilde{\Phi }_t&=V\tilde{\Phi }, \end{aligned}$$

where the coefficients U≡U(x,t,k) and V≡V(x,t,k) are defined by

2.2a $$\begin{aligned} U&=-\textrm{i}k \sigma _3+\frac{u_x}{2}\begin{pmatrix} 0& 1\\ -1& 0 \end{pmatrix},\end{aligned}$$

2.2b $$\begin{aligned} V&=-\frac{1}{4\textrm{i}k}\begin{pmatrix} \cos u& -\sin u\\ -\sin u& -\cos u \end{pmatrix}. \end{aligned}$$

The first step in the spectral analysis is to investigate the analyticity properties, in the complex spectral parameter *k*, of the eigenfunctions associated with the Lax pair. Studying their analyticity, symmetry, and boundedness in appropriate regions of the complex *k*-plane leads to precise relations among the different eigenfunctions. These relations can be formulated as a matrix Riemann–Hilbert factorization problem in the spectral variable.

Moreover, the coefficient matrices *U* and *V* in (2.2) are traceless. Consequently, for any matrix solution Φ(x,t,k) of the Lax pair (2.1) (constructed from two linearly independent vector solutions), we have

$$ \partial _x \det \Phi = 0, \qquad \partial _t \det \Phi = 0. $$

Hence, $\det \Phi$ is independent of both *x* and *t*, and therefore is constant throughout the domain.

### Problem I

Assume that we are given a solution *u*(*x*, *t*) of the Problem I (1.2) such that $u(\cdot , t)\in \tilde{H}^{1,2}(0,\infty )$ for all t∈[0,T]. We study the analytic properties of the associated eigenfunctions of the Lax pair in the complex spectral plane and derive relations among them that can be formulated as a factorization Riemann–Hilbert problem.

#### Eigenfunctions near k=∞

To have good control as $k\rightarrow \infty$, we rewrite the Lax pair (2.1) as

2.3a $$\begin{aligned} \tilde{\Phi }_x+\textrm{i}k \sigma _3\tilde{\Phi }&=U_\infty \tilde{\Phi },\end{aligned}$$

2.3b $$\begin{aligned} \tilde{\Phi }_t+\frac{1}{4\textrm{i}k}\sigma _3\tilde{\Phi }&=V_\infty \tilde{\Phi }, \end{aligned}$$

where the coefficients U≡U(x,t,k) and V≡V(x,t,k) are defined by

2.4a $$\begin{aligned} U_\infty&=\frac{u_x}{2}\begin{pmatrix} 0& 1\\ -1& 0 \end{pmatrix},\end{aligned}$$

2.4b $$\begin{aligned} V_\infty&=-\frac{1}{4\textrm{i}k}\begin{pmatrix} \cos u-1& -\sin u\\ -\sin u& -\cos u+1 \end{pmatrix}. \end{aligned}$$

Define *Q*(*x*, *t*, *k*) by

2.5a $$\begin{aligned} Q(x,t,k):= \textrm{i}k p(x,t,k)\sigma _3, \end{aligned}$$

with

2.5b $$\begin{aligned} p(x,t,k):=x-\frac{t}{4 k^2}. \end{aligned}$$

Then, *p* satisfies

$$ p_x=1,\qquad p_t=-\frac{1}{4 k^2},\qquad p(0,0,k)=0, $$

and the Lax pair (2.3) can be rewritten in the form

2.6a $$\begin{aligned}&\tilde{\Phi }_{ x}+Q_x\tilde{\Phi } = U_\infty \tilde{\Phi },\end{aligned}$$

2.6b $$\begin{aligned}&\tilde{\Phi }_{ t} +Q_t\tilde{\Phi } = V_\infty \tilde{\Phi }. \end{aligned}$$

We will see that this form is convenient for controlling the behavior of the corresponding solutions as $k\rightarrow \infty$.

We construct the solutions $\tilde{\Phi }_{ j}$, j=1,2,3, of Eq. (2.6) via solutions $\Phi _{ j}$ of the associated Volterra-type integral equations. The functions $\Phi _{ j}$ are uniquely determined by their initial integration points and are related to $\tilde{\Phi }_{ j}$ through

$$ \tilde{\Phi }_{ j}(x,t,\lambda ) = \Phi _{ j}(x,t,\lambda )\, e^{-\textrm{i}kp(x,t,\lambda )\sigma _3}. $$

They satisfy the integral equation

**Fig. 1:**
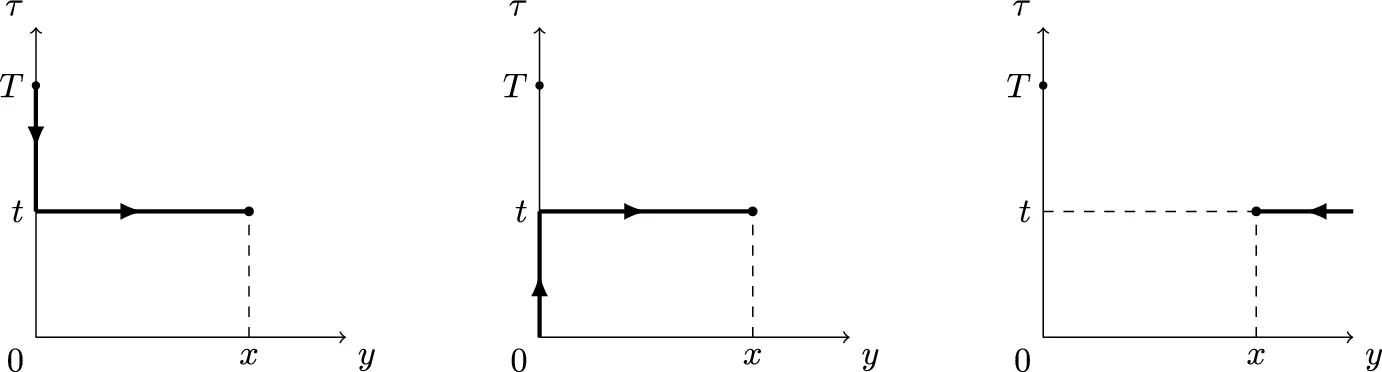
Paths of integration for $\Phi _{1}, \Phi _{2},$ and $\Phi _{3}$ ($\Phi _{01}, \Phi _{02},$ and $\Phi _{03}$ )

2.7 $$\begin{aligned} \Phi (x,t,k)=I+\int _{(x^*,t^*)}^{(x,t)} \textrm{e}^{\textrm{i}k(p(y,\tau ,k)-p(x,t,k))\hat{\sigma }_3}( U_\infty \Phi \textrm{d}y+V_\infty \Phi \textrm{d}\tau )(y,\tau ,k), \end{aligned}$$

where the initial point $(x^*,t^*)$ is chosen as (0, *T*), (0, 0), or (+∞,t) for j=1,2,3, respectively. The corresponding integration contours are shown in Fig. 1. Owing to the compatibility of the Lax pair, the value of the integral depends only on its endpoints.

More precisely,

2.8 $$\begin{aligned} \begin{aligned} \Phi _{ 1}(x,t,\lambda )=&I+ \int _{0}^{x} \textrm{e}^{-\textrm{i}k(x-y)\hat{\sigma }_3}( U_\infty \Phi _{1})(y,t,k) \textrm{d}y - \textrm{e}^{-\textrm{i}kx\hat{\sigma }_3} \\&\quad \int _{t}^{T} \textrm{e}^{ \frac{\tau -t}{4\textrm{i}k}\hat{\sigma }_3}( V_\infty \Phi _{1})(0,\tau ,k) \textrm{d}\tau , \end{aligned} \end{aligned}$$

2.9 $$\begin{aligned} \begin{aligned} \Phi _{ 2}(x,t,k)=&I+ \int _{0}^{x} \textrm{e}^{-\textrm{i}k(x-y)\hat{\sigma }_3}( U_\infty \Phi _{2})(y,t,k) \textrm{d}y + \textrm{e}^{-\textrm{i}kx\hat{\sigma }_3} \\&\quad \int _{0}^{t} \textrm{e}^{\frac{\tau -t}{4\textrm{i}k}\hat{\sigma }_3}( V_\infty \Phi _{2})(0,\tau ,k) \textrm{d}\tau , \end{aligned} \end{aligned}$$

2.10 $$\begin{aligned} \Phi _{ 3}(x,t,k)=I- \int _{x}^{+\infty } \textrm{e}^{\textrm{i}k(y-x)\hat{\sigma }_3}( U_\infty \Phi _{3})(y,t,k) \textrm{d}y . \end{aligned}$$

**Fig. 2:**
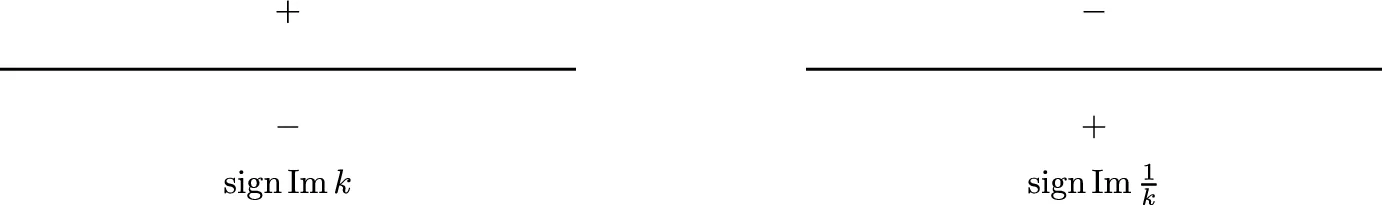
Signature tables for *p*(*x*, *t*, *k*)

The domains where the exponentials are bounded in the complex *k*-plane are separated by the real axis; see Fig. 2. This partition of the *k*-plane is determined by the signature table of $\operatorname {Im}k$ and $\operatorname {Im}\frac{1}{k}$.

Let $A^{(1)}$ and $A^{(2)}$ denote the first and second columns of a 2×2 matrix $A = \bigl ( A^{(1)}\ \ A^{(2)} \bigr )$ and notice that if $f\in \tilde{H}^{1,2}(0,\infty )$, then $f_x,~\sin f,~1-\cos f\in L^1(0,\infty )$. Since we assumed that $u(\cdot , t)\in \tilde{H}^{1,2}(0,\infty )$, it follows that the Neumann series expansions of (2.8)–(2.10) converge in the corresponding half-planes of *k* and yields the following properties of $\Phi _{i}^{(j)}(x,t,k)$:

1. $\Phi _{ 1}^{(1)}(x,t,k)$ is analytic in $\mathbb {C}\setminus \{0\}$ and $$\Phi _{1}^{(1)}(x,t,k)=\begin{pmatrix} 1\\ 0 \end{pmatrix}+O(\frac{1}{k})$$ as $k\rightarrow \infty$ in $\overline{\mathbb {C}^+}$. Moreover, $$\Phi _{1}^{(1)}(0,t,k)=\begin{pmatrix} 1\\ 0 \end{pmatrix}+O(\frac{1}{k})$$ as $k\rightarrow \infty$ in $\mathbb {C}$.
2. $\Phi _{ 1}^{(2)}(x,t,k)$ is analytic in $\mathbb {C}\setminus \{0\}$ and $$\Phi _{ 1}^{(2)}(x,t,k)=\begin{pmatrix} 0\\ 1 \end{pmatrix}+O(\frac{1}{k})$$ as $k\rightarrow \infty$ in $\overline{\mathbb {C}^-}$. Moreover, $$\Phi _{ 1}^{(2)}(0,t,k)=\begin{pmatrix} 0\\ 1 \end{pmatrix}+O(\frac{1}{k})$$ as $k\rightarrow \infty$ in $\mathbb {C}$.
3. $\Phi _{ 2}^{(1)}(x,t,k)$ is analytic in $\mathbb {C}\setminus \{0\}$ and $$\Phi _{ 2}^{(1)}(x,t,k)=\begin{pmatrix} 1\\ 0 \end{pmatrix}+O(\frac{1}{k})$$ as $k\rightarrow \infty$ in $\overline{\mathbb {C}^+}$.
4. $\Phi _{ 2}^{(2)}(x,t,k)$ is analytic in $\mathbb {C}\setminus \{0\}$ and $$\Phi _{ 2}^{(2)}(x,t,k)=\begin{pmatrix} 0\\ 1 \end{pmatrix}+O(\frac{1}{k})$$ as $k\rightarrow \infty$ in $\overline{\mathbb {C}^-}$.
5. $\Phi _{ 3}^{(1)}(x,t,k)$ is analytic in $\mathbb {C}^-$ and continuous up to real line. Moreover, $\Phi _{ 3}^{(1)}(x,t,k)$=$$\begin{pmatrix} 1\\ 0 \end{pmatrix}+O(\frac{1}{k})$$ as $k\rightarrow \infty$ in $\overline{\mathbb {C}^-}$.
6. $\Phi _{ 3}^{(2)}(x,t,k)$ is analytic in $\mathbb {C}^+$ and continuous up to real line. Moreover, $$\Phi _{3}^{(2)}(x,t,k)=\begin{pmatrix} 0\\ 1 \end{pmatrix}+O(\frac{1}{k})$$ as $k\rightarrow \infty$ in $\overline{\mathbb {C}^+}$.

Since the coefficient matrices in the Lax pair (2.3) are traceless, it follows that

2.11 $$\begin{aligned} \det \Phi _{ j} \equiv 1, \qquad j=1,2,3. \end{aligned}$$

Furthermore, the matrices $U_\infty (k)\equiv U_\infty (x,t,k)$ and $V_\infty (k)\equiv V_\infty (x,t,k)$ obey the symmetries

2.12a $$\begin{aligned} U_\infty (- k)&=\overline{U_\infty (\bar{k})},&\qquad&U_\infty (-k)=\sigma _2U_\infty (k)\sigma _2, \end{aligned}$$

2.12b $$\begin{aligned} V_\infty (- k)&=\overline{V_\infty (\bar{k})},  &   V_\infty (-k)=\sigma _2V_\infty (k)\sigma _2, \end{aligned}$$

while the function p(k)≡p(x,t,k) satisfies

2.13 $$\begin{aligned} p^*(k)=p(k)=p(-k). \end{aligned}$$

Consequently, the eigenfunctions $\Phi _{ j}$ inherit the same symmetries:

2.14 $$\begin{aligned} \Phi _{ j}(-k)=\overline{\Phi _{ j}(\bar{k})},\qquad \Phi _{ j}(-k)=\sigma _2\Phi _{ j}(k)\sigma _2. \end{aligned}$$

Since the functions $\tilde{\Phi }_{ j}$ solve both equations of (2.3), they are related (where defined) by matrices independent of *x* and *t*. Hence,

2.15a $$\begin{aligned}&\Phi _{ 3}(x,t,k)=\Phi _{ 2}(x,t,k)\textrm{e}^{-\textrm{i}k p(x,t,k)\sigma _3}s(k)\textrm{e}^{\textrm{i}k p(x,t,k)\sigma _3},\qquad k\in \mathbb {R}\setminus \{0\} \end{aligned}$$

2.15b $$\begin{aligned}&\Phi _{ 1}(x,t,k)=\Phi _{2}(x,t,k)\textrm{e}^{-\textrm{i}k p(x,t,k)\sigma _3}S(k)\textrm{e}^{\textrm{i}k p(x,t,k)\sigma _3}, \qquad k\in \mathbb {C}\setminus \{0\}. \end{aligned}$$

The symmetries (2.14) imply that the matrices *s*(*k*) and *S*(*k*) admit the forms

2.16 $$\begin{aligned} s(k)=\Phi _{\infty 3}(0,0,k)=\begin{pmatrix} a^*(k) &  b(k)\\ -b^*( k) &  a(k) \end{pmatrix} \end{aligned}$$

and

2.17 $$\begin{aligned} S(k)=\Phi _{\infty 1}(0,0,k)=\begin{pmatrix} A^*(k) &  B(k)\\ -B^*(k) &  A(k) \end{pmatrix}\end{aligned}$$

with some *a*(*k*), *b*(*k*), *A*(*k*), and *B*(*k*).

Relation (2.15a) constitutes the scattering relation for the *x*-equation (2.4a), (2.4a) (with *t* being a parameter). Likewise, relation (2.15b) constitutes the scattering relation for the *t*-equation (2.4b), (2.4b) (with *x* being a parameter).

#### Eigenfunctions near k=0

In order to have good control as k→0, we introduce

$$ \tilde{\Phi }_0(x,t,k):= P(x,t)\tilde{\Phi }(x,t,k), $$

where

$$ P(x,t):=\begin{pmatrix} \cos \frac{u}{2} &  -\sin \frac{u}{2} \\ \sin \frac{u}{2} &  \cos \frac{u}{2} \\ \end{pmatrix}. $$

This transforms (2.1) into

2.18a $$\begin{aligned}&\tilde{\Phi }_{0 x}+\textrm{i}k\sigma _3\tilde{\Phi }_0=U_0\tilde{\Phi }_0,\end{aligned}$$

2.18b $$\begin{aligned}&\tilde{\Phi }_{0 t}+\frac{1}{4\textrm{i}k}\sigma _3\tilde{\Phi }_0=V_0 \tilde{\Phi }_0, \end{aligned}$$

where

2.19a $$\begin{aligned} U_0&=-\textrm{i}k \begin{pmatrix} \cos u-1& \sin u\\ \sin u& -\cos u+1 \end{pmatrix},\end{aligned}$$

2.19b $$\begin{aligned} V_0&=\frac{ u_t}{2}\begin{pmatrix} 0& -1\\ 1& 0 \end{pmatrix}. \end{aligned}$$

We introduce the solutions $\tilde{\Phi }_{0j}(x,t,k)$, j=1,2,3, of (2.18) analogously to $\tilde{\Phi }_{ j}(x,t,k)$ by defining

$$ \Phi _{0}(x,t,k):=\tilde{\Phi }_{0}(x,t,k)\textrm{e}^{\textrm{i}k p(x,t,k)\sigma _3}, $$

where the functions $\Phi _{0j}(x,t,k)$ solve the associated integral equations

2.20 $$\begin{aligned} \Phi _{0}(x,t,k)=I+\int _{(x^*,t^*)}^{(x,t)} \textrm{e}^{\textrm{i}k(p(y,\tau ,k)-p(x,t,k))\hat{\sigma }_3}( U_0\Phi _0 \textrm{d}y+V_0\Phi _0 \textrm{d}\tau )(y,\tau ,k). \end{aligned}$$

More precisely,

2.21 $$\begin{aligned} \begin{aligned} \Phi _{0 1}(x,t,\lambda )=&I+ \int _{0}^{x} \textrm{e}^{-\textrm{i}k(x-y)\hat{\sigma }_3}( U_0\Phi _{01})(y,t,k) \textrm{d}y - \textrm{e}^{-\textrm{i}kx\hat{\sigma }_3} \\&\quad \int _{t}^{T} \textrm{e}^{ \frac{\tau -t}{4\textrm{i}k}\hat{\sigma }_3}( V_0\Phi _{01})(0,\tau ,k) \textrm{d}\tau , \end{aligned} \end{aligned}$$

2.22 $$\begin{aligned} \begin{aligned} \Phi _{ 02}(x,t,k)=&I+ \int _{0}^{x} \textrm{e}^{-\textrm{i}k(x-y)\hat{\sigma }_3}( U_0\Phi _{02})(y,t,k) \textrm{d}y + \textrm{e}^{-\textrm{i}kx\hat{\sigma }_3} \\&\quad \int _{0}^{t} \textrm{e}^{\frac{\tau -t}{4\textrm{i}k}\hat{\sigma }_3}( V_0\Phi _{02})(0,\tau ,k) \textrm{d}\tau , \end{aligned} \end{aligned}$$

2.23 $$\begin{aligned} \Phi _{0 3}(x,t,k)=I- \int _{x}^{+\infty } \textrm{e}^{\textrm{i}k(y-x)\hat{\sigma }_3}( U_0\Phi _{03})(y,t,k) \textrm{d}y . \end{aligned}$$

The following properties of $\Phi _{0 i}^{(j)}(x,t,k)$ follow from the Neumann series for (2.21)–(2.23):

1. $\Phi _{0 1}^{(1)}(x,t,k)$ is analytic in $\mathbb {C}\setminus \{0\}$ and $$\Phi _{01}^{(1)}(x,t,k)=\begin{pmatrix} 1\\ 0 \end{pmatrix}+O(k)$$ as k→0 in $\mathbb {C}^-$.
2. $\Phi _{0 1}^{(2)}(x,t,k)$ is analytic in $\mathbb {C}\setminus \{0\}$ and $$\Phi _{0 1}^{(2)}(x,t,k)=\begin{pmatrix} 0\\ 1 \end{pmatrix}+O(k)$$ as k→0 in $\mathbb {C}^+$.
3. $\Phi _{0 2}^{(1)}(x,t,k)$ is analytic in $\mathbb {C}\setminus \{0\}$ and $$\Phi _{0 2}^{(1)}(x,t,k)=\begin{pmatrix} 1\\ 0 \end{pmatrix}+O(k)$$ as k→0 in $\mathbb {C}^+$.
4. $\Phi _{0 2}^{(2)}(x,t,k)$ is analytic in $\mathbb {C}\setminus \{0\}$ and $$\Phi _{0 2}^{(2)}(x,t,k)=\begin{pmatrix} 0\\ 1 \end{pmatrix}+O(k)$$ as k→0 in $\mathbb {C}^-$.
5. $\Phi _{0 3}^{(1)}(x,t,k)$ is analytic in $\mathbb {C}^-$ and continuous up to the boundary. Moreover, $\Phi _{0 3}^{(1)}(x,t,k)$= $$\begin{pmatrix} 1\\ 0 \end{pmatrix}+O(k)$$ as k→0 in $\mathbb {C}^-$.
6. $\Phi _{0 3}^{(2)}(x,t,k)$ is analytic in $\mathbb {C}^+$ and continuous up to the boundary. Moreover, $$\Phi _{03}^{(2)}(x,t,k)=\begin{pmatrix} 0\\ 1 \end{pmatrix}+O(k)$$ as k→0 in $\mathbb {C}^+$.

Since the coefficient matrices in the Lax pair (2.18) are traceless, it follows that

$$ \det \Phi _{0 j} \equiv 1, \qquad j=1,2,3. $$

Moreover, because the matrices $$U_0(x,t,k)$$, $$ V_0(x,t,k)$$ satisfy the symmetries (2.12), the functions $$\Phi _{0 j}(x,t,k)$$ inherit the symmetries (2.14).

Since the functions $\tilde{\Phi }_{0 j}$satisfy both equations of the Lax pair (2.18), they are related (where defined) by matrices independent of *x* and *t*; hence

2.24a $$\begin{aligned}&\Phi _{0 3}(x,t,k)=\Phi _{0 2}(x,t,k)\textrm{e}^{-\textrm{i}kp(x,t,k)\sigma _3}\tilde{s}(k)\textrm{e}^{\textrm{i}k p(x,t,k)\sigma _3},\qquad k\in \mathbb {R}\setminus \{0\}\end{aligned}$$

2.24b $$\begin{aligned}&\Phi _{0 1}(x,t,k)=\Phi _{0 2}(x,t,k)\textrm{e}^{-\textrm{i}k p(x,t,k)\sigma _3}\tilde{S}(k)\textrm{e}^{\textrm{i}k p(x,t,k)\sigma _3},\qquad k\in \mathbb {C}\setminus \{0\}. \end{aligned}$$

Due to the symmetries of $\Phi _{0 j}$, $\tilde{s}(k)$ and $\tilde{S}(k)$ admit the forms

2.25 $$\begin{aligned} \tilde{s}(k)=\Phi _{0 3}(0,0,k)=\begin{pmatrix} \tilde{a}^*(k) & \quad \tilde{b}(k)\\ -\tilde{b}^*(k) &  \quad \tilde{a}(k) \end{pmatrix} \end{aligned}$$

and

2.26 $$\begin{aligned} \tilde{S}(k)=\Phi _{0 1}(0,0,k)=\begin{pmatrix} \tilde{A}^*(k) &  \quad \tilde{B}(k)\\ -\tilde{B}^*(k) & \quad \tilde{A}(k) \end{pmatrix}\end{aligned}$$

with some $\tilde{a}(k)$, $\tilde{b}(k)$, $\tilde{A}(k)$, and $\tilde{B}(k)$.

#### Relations between eigenfunctions of different Lax pairs

Since $\Phi _0$ and Φ arise from the same system (2.1), they are related as follows:

2.27a $$\begin{aligned} \Phi _{ j}(x,t,k)=P^{-1}(x,t)\Phi _{0 j}(x,t,k)\textrm{e}^{-\textrm{i}kp(x,t,k)\sigma _3}C_j(k)\textrm{e}^{\textrm{i}k p(x,t,k)\sigma _3} \end{aligned}$$

with $C_j(k)$ given by

2.27b $$\begin{aligned} C_1&=\textrm{e}^{-\frac{\textrm{i}T}{4 k}\sigma _3}P(0,T)\textrm{e}^{\frac{\textrm{i}T}{4 k}\sigma _3},\end{aligned}$$

2.27c $$\begin{aligned} C_2&=P(0,0),\end{aligned}$$

2.27d $$\begin{aligned} C_3&=I. \end{aligned}$$

Particularly,

2.28 $$\begin{aligned} \Phi _{ 3}(x,t,k) =P^{-1}(x,t)\Phi _{0 3}(x,t,k). \end{aligned}$$

Furthermore, setting (x,t)=(0,0) in (2.27) gives

2.29 $$\begin{aligned}&s(k)=P^{-1}(0,0)\tilde{s}(k),\end{aligned}$$

2.30 $$\begin{aligned}&S(k)=P^{-1}(0,0)\tilde{S}(k)C_1=P^{-1}(0,0)\tilde{S}(k)\textrm{e}^{-\frac{\textrm{i}T}{4 k}\sigma _3}P(0,T)\textrm{e}^{\frac{\textrm{i}T}{4 k}\sigma _3} \end{aligned}$$

##### Remark 2.1

In the case u(0,0)=4πk, we have $P^{-1}(0,0)=I$ and thus (2.27a) for j=2 reduces to

2.31 $$\begin{aligned} \Phi _{ 2}(x,t,k) =P^{-1}(x,t)\Phi _{0 2}(x,t,k). \end{aligned}$$

Consequently, in this case

$$ s(k)=\tilde{s}(k), $$

or

$$ a(k) = \tilde{a}(k),\quad b(k) = \tilde{b}(k), $$

which results in a significant simplification of the subsequent analysis and constructions.

In the general case, we set

$$ P^{-1}(0,0)= \begin{pmatrix} \kappa _1^0 &  \quad \kappa _2^0 \\ -\kappa _2^0 &  \quad \kappa _1^0 \end{pmatrix}, $$

where $\kappa _1^0$ and $\kappa _2^0$ are determined by *u*(0, 0) through

2.32 $$\begin{aligned} \kappa _1^0:=\cos \tfrac{u(0,0)}{2}, \qquad \kappa _2^0:=\sin \tfrac{u(0,0)}{2}. \end{aligned}$$

With this notation, (2.29) reads as

2.33 $$\begin{aligned} \begin{aligned}&a(k)=-\kappa _2^0\tilde{b}(k)+\kappa _1^0\tilde{a}(k),\\&b(k)=\kappa _1^0\tilde{b}(k)+\kappa _2^0\tilde{a}(k). \end{aligned} \end{aligned}$$

Similarly, introducing

$$ \kappa _1^T:=\cos \tfrac{u(0,T)}{2}, \qquad \kappa _2^T:=\sin \tfrac{u(0,T)}{2}, $$

(2.30) reads as

2.34 $$\begin{aligned} \begin{aligned}&\tilde{A}(k)=e^{-\frac{\textrm{i}T}{2k}} \,\kappa _2^T\big (\kappa _2^0 A^*(k)-\kappa _1^0 B^*(k)\big ) + \kappa _1^T\big (\kappa _2^0 B(k)+\kappa _1^0 A(k)\big ),\\&\tilde{B}(k)=e^{-\frac{\textrm{i}T}{2k}} \,\kappa _2^T\big (\kappa _1^0 A^*(k)+\kappa _2^0 B^*(k)\big ) + \kappa _1^T\big (\kappa _1^0 B(k)-\kappa _2^0 A(k)\big ), \end{aligned} \end{aligned}$$

or, in the reverse order,

2.35 $$\begin{aligned} \begin{aligned}&A(k) =\kappa _1^T\left( \kappa _1^0\tilde{A}(k) -\kappa _2^0\tilde{B}(k)\right) + e^{-\frac{iT}{2k}}\kappa _2^T \left( \kappa _1^0\tilde{B}^*(k)+\kappa _2^0\tilde{A}^*(k)\right) ,\\&B(k) =\kappa _1^T\left( \kappa _1^0\tilde{B}(k) +\kappa _2^0\tilde{A}(k)\right) + e^{\frac{iT}{2k}} \kappa _2^T \left( \kappa _2^0\tilde{B}^*(k)-\kappa _1^0\tilde{A}^*(k)\right) . \end{aligned} \end{aligned}$$

#### The direct *x*-spectral problem $\{ u_{0}(x) \} \mapsto \{a(k), b(k) \}$

Given a function $u_{0}(x)\in H^{1,1}((0,\infty ))+2\pi k$ (not necessarily arising as the initial value of a solution of the sG equation), we define the direct *x*-spectral mapping

$$ \{ u_{0x}(x) \} \longrightarrow \{ {a}(k), {b}(k) \} $$

by considering Eq. (2.10) for t=0:

2.36 $$\begin{aligned} \Phi _{ 3}(x,0,k)=I- \int _{x}^{+\infty } \textrm{e}^{\textrm{i}k (y-x)\hat{\sigma }_3}( U_\infty \Phi _{3})(y,0,k) \textrm{d}y, \end{aligned}$$

where $U_\infty$ is given by (2.4a) with $u_x(x,0)$ replaced by $u_{0x}(x)$.

Then, *a*(*k*) and *b*(*k*) are defined by (cf. (2.16))

$$ \begin{pmatrix} {b}(k) \\ {a}(k) \end{pmatrix} = \Phi _{\infty 3}^{(2)}(0,0,k) $$

and the analysis of the Volterra integral equation (2.36) yields the following properties:

1. *a*(*k*) and *b*(*k*) are analytic in $\mathbb {C}^+$ and continuous up to the boundary except at k=0. Moreover, $a(k)=1+O(\frac{1}{k})$ and $b(k)=\frac{u_{0x}(0)}{4\textrm{i}k}+O(\frac{1}{k^2})$ as $k\rightarrow \infty$ in $\overline{\mathbb {C}^{+}}$.
2. Determinant relation:
  2.37 $$\begin{aligned} a(k) a^*( k)+ b(k) b^*( k)=1, \qquad k\in \mathbb {R}. \end{aligned}$$
3. Symmetries:
  2.38 $$\begin{aligned} \overline{a(\bar{k})}=a(-k),\qquad \overline{b(\bar{k})}=b(-k). \end{aligned}$$

Similarly, we define the direct *x*-spectral mapping

$$ \{\sin u_{0}(x),\cos u_{0}(x) \} \longrightarrow \{\tilde{a}(k),\tilde{b}(k) \} $$

by considering Eq. (2.23) for t=0:

2.39 $$\begin{aligned} \Phi _{0 3}(x,0,k)=I- \int _{x}^{+\infty } \textrm{e}^{\textrm{i}k(y-x)\hat{\sigma }_3}( U_0\Phi _{03})(y,0,k) \textrm{d}y,\qquad x\ge 0 \end{aligned}$$

where $U_0$ is given by (2.19a) with sinu(x,0) and cosu(x,0) replaced by $\sin u_0(x)$ and $\cos u_0(x)$, respectively. Then, $\tilde{a}(k)$ and $\tilde{b}(k)$ defined by (cf. (2.25))

$$ \begin{pmatrix} \tilde{b}(k) \\ \tilde{a}(k) \end{pmatrix} = \Phi _{03}^{(2)}(0,0,k) $$

have the following properties:

1. $\tilde{a}(k)$ and $\tilde{b}(k)$ are analytic in $\mathbb {C}^+$ and continuous up to the boundary. Moreover, $\tilde{a}(k)=1+O(k)$ and $\tilde{b}(k)=O(k)$ as k→0 in $\mathbb {C}^+$.
2. Determinant relation:
  2.40 $$\begin{aligned} \tilde{a}(k)\tilde{a}^*( k)+ \tilde{b}(k)\tilde{b}^*( k)=1, \qquad k\in \mathbb {R}; \end{aligned}$$
3. Symmetries:
  2.41 $$\begin{aligned} \overline{\tilde{a}(\bar{k})}=\tilde{a}(-k),\qquad \overline{\tilde{b}(\bar{k})}=\tilde{b}(-k). \end{aligned}$$

Moreover, taking into account (2.33), it follows that we can specify the behavior of *a*(*k*) and *b*(*k*) as k→0

2.42 $$\begin{aligned} a(k)=\kappa _1^0+O(k), \qquad b(k)=\kappa _2^0+O(k), \qquad k\in \mathbb {C}^+. \end{aligned}$$

##### Remark 2.2

The spectral functions *a*(*k*) and *b*(*k*) are determined solely by $u_{0x}(x)$ assuming that $u_{0x}(x)\in L^1(0,+\infty )$, whereas the spectral functions $\tilde{a}(k)$ and $\tilde{b}(k)$ are determined by $\sin u_0(x)$ and $\cos u_0(x)$. In particular, it is sufficient to know $u_0(x)$ modulo 2π in order to define the direct *x*-spectral problems.

Notice that the requirement $u_0(x)\in H^{2,2}((0,+\infty ))+2\pi k$ in Problem I will provide additional properties of *a*(*k*), *b*(*k*), $\tilde{a}(k)$, and $\tilde{b}(k)$ needed for a particular regularity of the RH problem with respect to its parameters (see Sects. 6 and 7)

#### The direct *t*-spectral problem $\{ v_{0}(t) \} \mapsto \{ A(k), B(k) \}$

Given a function $v_0(t)\in L^1(0,T)$ we define the direct *t*-spectral mapping

$$ \{\sin v_0(t), \cos v_0(t) \} \longrightarrow \{ A(k), B(k) \} $$

by considering Eq. (2.8) for x=0

2.43 $$\begin{aligned} \Phi _{ 1}(0,t,k)=I-\int _{t}^{T} \textrm{e}^{ \frac{\tau -t}{4\textrm{i}k}\hat{\sigma }_3}( V_\infty \Phi _{1})(0,\tau ,k) \textrm{d}\tau , \end{aligned}$$

where $V_\infty$ is given by (2.4b) with sinu(0,t) and cosu(0,t) replaced by $\sin v_0(t)$ and $\cos v_0(t)$, respectively.

Then *A*(*k*) and *B*(*k*) are defined by (cf. (2.17))

$$ \begin{pmatrix} {A}(k) \\ B(k) \end{pmatrix} = \Phi _{\infty 1}^{(2)}(0,0,k) $$

and satisfy the following properties:

1. *A*(*k*) and *B*(*k*) are analytic in $\mathbb {C}{\setminus }\{0\}$. Moreover, $A(k)=1+O(\frac{1}{k})$ and $B(k)=O(\frac{1}{k})$ as $k\rightarrow \infty$.
2. Determinant relation:
  2.44 $$\begin{aligned} A(k) A^*( k)+ B(k) B^*(k)=1 \end{aligned}$$
3. Symmetries:
  2.45 $$\begin{aligned} \overline{A(\bar{k})}=A(-k),\qquad \overline{B(\bar{k})}=B(-k). \end{aligned}$$

Similarly, we define the direct *t*-spectral mapping

$$ \{ v_{0t}(t) \} \longrightarrow \{\tilde{A}(k),\tilde{B}(k) \} $$

by considering (2.21) for x=0:

2.46 $$\begin{aligned} \Phi _{0 1}(0,t,k)=I- \int _{t}^{T} \textrm{e}^{-\frac{\textrm{i}}{4k}(\tau -t)\hat{\sigma }_3}( V_0\Phi _{01})(0,\tau ,k ) \textrm{d}\tau , \end{aligned}$$

where $V_0$ is given by (2.19b) with $u_t(0,t)$ replaced by $v_{0t}(t)$. Then, $\tilde{A}(k)$ and $\tilde{B}(k)$ defined by (cf. (2.25))

$$ \begin{pmatrix} \tilde{B}(k) \\ \tilde{A}(k) \end{pmatrix} = \Phi _{01}^{(2)}(0,0,k) $$

have the following properties:

1. $\tilde{A}(k)$ and $\tilde{B}(k)$ are analytic in $\mathbb {C}{\setminus }\{0\}$. Moreover, $\tilde{A}(k)=1+O(k)+O\left( k\textrm{e}^{-\frac{\textrm{i}T}{2k}} \right)$ and $\tilde{B}(k)=O(k)+O\left( k\textrm{e}^{-\frac{\textrm{i}T}{2k}}\right)$ as k→0.
2. Determinant relation:
  2.47 $$\begin{aligned} \tilde{A}(k)\tilde{A}^*(k)+ \tilde{B}(k)\tilde{B}^*(k)=1 \end{aligned}$$
3. Symmetries:
  2.48 $$\begin{aligned} \overline{\tilde{A}(\bar{k})}=\tilde{A}(-k),\qquad \overline{\tilde{B}(\bar{k})}=\tilde{B}(-k). \end{aligned}$$
4. Behavior at ∞ (follows from (2.34)):
  2.49 $$\begin{aligned} \tilde{A}(k)=\kappa _1^T\kappa _1^0+\kappa _2^T\kappa _2^0+O\left( \frac{1}{k}\right) ,\quad \tilde{B}(k)=-\kappa _1^T\kappa _2^0+\kappa _2^T\kappa _1^0+O\left( \frac{1}{k}\right) ,\quad k\rightarrow \infty . \end{aligned}$$

Furthermore, (2.35) allows us to specify the behavior of *A*(*k*) and *B*(*k*) as k→0, namely

2.50 $$\begin{aligned} A(k)= &   \kappa _1^T\kappa _1^0 + \kappa _2^T\kappa _2^0 \textrm{e}^{-\frac{\textrm{i}T}{2k}}+O(k)+O\left( k\textrm{e}^{-\frac{\textrm{i}T}{2k}} \right) , \nonumber \\ B(k)= &   \kappa _1^T\kappa _2^0 + \kappa _2^T\kappa _1^0 \textrm{e}^{-\frac{\textrm{i}T}{2k}}+O(k)+O\left( k\textrm{e}^{-\frac{\textrm{i}T}{2k}} \right) . \end{aligned}$$

### Problem II

As in the case of Problem I, assuming that we are given a solution *u*(*x*, *t*) of Problem II (1.3) such that $u(\cdot , t)\in \tilde{H}^{1,2}(-\infty ,0)$ for all t∈[0,T], we study the analytic properties of the associated eigenfunctions.

**Fig. 3:**
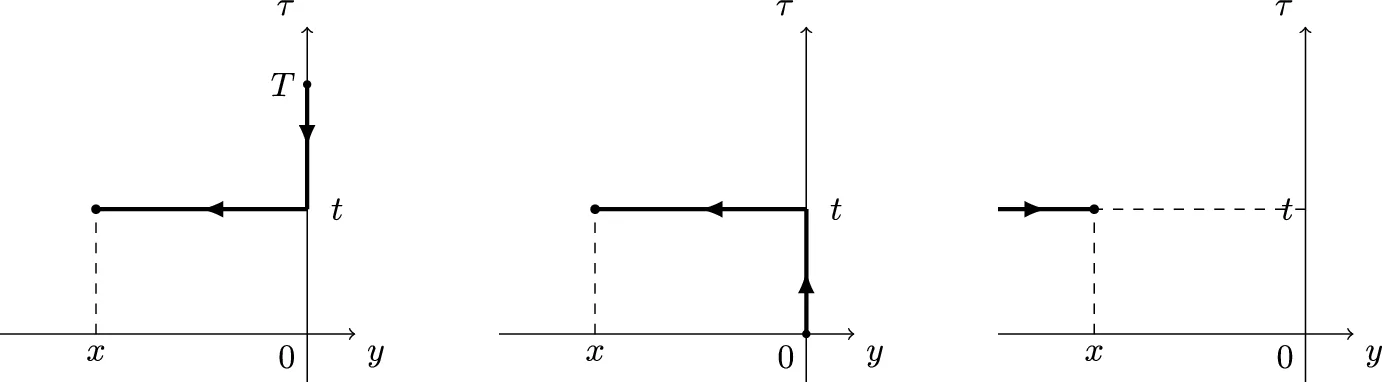
Paths of integration for $\Phi _{1}, \Phi _{2},$ and $\check{\Phi }_{3}$ ($\Phi _{01}, \Phi _{02},$ and $\check{\Phi }_{03}$ )

We define the eigenfunctions Φ as in (2.7). However, instead of $\Phi _{ 3}$ we introduce $\check{\Phi }_{3}$, for which the initial point $(x^*,t^*)$ is chosen to be (-∞,t). The integration paths are those shown in Fig. 3.

More precisely,

2.51 $$\begin{aligned} \begin{aligned} \Phi _{ 1}(x,t,\lambda )=I&- \int _{x}^{0} \textrm{e}^{\textrm{i}k(y-x)\hat{\sigma }_3}( U_\infty \Phi _{1})(y,t,k) \textrm{d}y -\textrm{e}^{-\textrm{i}kx\hat{\sigma }_3} \\&\quad \int _{t}^{T} \textrm{e}^{ \frac{\tau -t}{4\textrm{i}k }\hat{\sigma }_3}( V_\infty \Phi _{1})(0,\tau ,k) \textrm{d}\tau , \end{aligned} \end{aligned}$$

2.52 $$\begin{aligned} \begin{aligned} \Phi _{ 2}(x,t,k)=I&- \int _{x}^{0} \textrm{e}^{\textrm{i}k(y-x)\hat{\sigma }_3}( U_\infty \Phi _{2})(y,t,k) \textrm{d}y +\textrm{e}^{-\textrm{i}kx\hat{\sigma }_3} \\&\quad \int _{0}^{t} \textrm{e}^{ \frac{\tau -t}{4\textrm{i}k }\hat{\sigma }_3}( V_\infty \Phi _{2})(0,\tau ,k) \textrm{d}\tau , \end{aligned} \end{aligned}$$

2.53 $$\begin{aligned} \check{\Phi }_{3}(x,t,k)=I+ \int ^{x}_{-\infty } \textrm{e}^{-\textrm{i}k(x-y)\hat{\sigma }_3}( U_\infty \check{\Phi }_{3})(y,t,k) \textrm{d}y. \end{aligned}$$

They have the following properties:

1. $\Phi _{ 1}^{(1)}(x,t,k)$ is analytic in $\mathbb {C}\setminus \{0\}$ and $\Phi _{ 1}^{(1)}(x,t,k)$=$$\begin{pmatrix} 1\\ 0 \end{pmatrix}+O(\frac{1}{k})$$ as $k\rightarrow \infty$ in $\overline{\mathbb {C}^-}$. Moreover, $\Phi _{ 1}^{(1)}(0,t,k)$=$$\begin{pmatrix} 1\\ 0 \end{pmatrix}+O(\frac{1}{k})$$ as $k\rightarrow \infty$ in $\mathbb {C}$.
2. $\Phi _{ 1}^{(2)}(x,t,k)$ is analytic in $\mathbb {C}\setminus \{0\}$ and $\Phi _{ 1}^{(2)}(x,t,k)$=$$\begin{pmatrix} 0\\ 1 \end{pmatrix}+O(\frac{1}{k})$$ as $k\rightarrow \infty$ in $\overline{\mathbb {C}^+}$. Moreover, $\Phi _{ 1}^{(2)}(0,t,k)$=$$\begin{pmatrix} 0\\ 1 \end{pmatrix}+O(\frac{1}{k})$$ as $k\rightarrow \infty$ in $\mathbb {C}$.
3. $\Phi _{ 2}^{(1)}(x,t,k)$ is analytic in $\mathbb {C}\setminus \{0\}$ and $\Phi _{\infty 2}^{(1)}(x,t,k)$=$$\begin{pmatrix} 1\\ 0 \end{pmatrix}+O(\frac{1}{k})$$ as $k\rightarrow \infty$ in $\overline{\mathbb {C}^-}$.
4. $\Phi _{ 2}^{(2)}(x,t,k)$ is analytic in $\mathbb {C}\setminus \{0\}$ and $\Phi _{ 2}^{(2)}(x,t,k)$=$$\begin{pmatrix} 0\\ 1 \end{pmatrix}+O(\frac{1}{k})$$ as $k\rightarrow \infty$ in $\overline{\mathbb {C}^+}$.
5. $\check{\Phi }_{3}^{(1)}(x,t,k)$ is analytic in $\mathbb {C}^+$ and continuous up to real line. Moreover, $\check{\Phi }_{3}^{(1)}(x,t,k)$=$$\begin{pmatrix} 1\\ 0 \end{pmatrix}+O(\frac{1}{k})$$ as $k\rightarrow \infty$ in $\overline{\mathbb {C}^+}$.
6. $\check{\Phi }_{3}^{(2)}(x,t,k)$ is analytic in $\mathbb {C}^-$ and continuous up to real line. Moreover, $\check{\Phi }_{3}^{(2)}(x,t,k)$=$$\begin{pmatrix} 0\\ 1 \end{pmatrix}+O(\frac{1}{k})$$ as $k\rightarrow \infty$ in $\overline{\mathbb {C}^-}$.

Similarly, we define the eigenfunctions $\Phi _0$ as the solutions of the following integral equations:

2.54 $$\begin{aligned} \begin{aligned} \Phi _{0 1}(x,t,k)&=I- \int _{x}^{0} \textrm{e}^{-\textrm{i}k(x-y)\hat{\sigma }_3}( U_0\Phi _{01})(y,t,k) \textrm{d}y -\textrm{e}^{-\textrm{i}k x\hat{\sigma }_3} \\&\quad \int _{t}^{T} \textrm{e}^{\frac{i}{4 k}(t-\tau )\hat{\sigma }_3}( V_0\Phi _{01})(0,\tau ,k) \textrm{d}\tau , \end{aligned} \end{aligned}$$

2.55 $$\begin{aligned} \begin{aligned} \Phi _{02}(x,t,k)&=I- \int _{x}^{0} \textrm{e}^{-\textrm{i}k(x-y)\hat{\sigma }_3}( U_0\Phi _{02})(y,t,k) \textrm{d}y +\textrm{e}^{-\textrm{i}k x\hat{\sigma }_3} \\&\quad \int _{0}^{t} \textrm{e}^{\frac{i}{4 k}(t-\tau )\hat{\sigma }_3}( V_0\Phi _{02})(0,\tau ,k) \textrm{d}\tau , \end{aligned} \end{aligned}$$

2.56 $$\begin{aligned} \check{\Phi }_{03}(x,t,k)=I+ \int _{-\infty }^{x} \textrm{e}^{-\textrm{i}k (x-y)\hat{\sigma }_3}( U_0\check{\Phi }_{03})(y,t,k) \textrm{d}y, \end{aligned}$$

and using the Neumann series, we obtain the following properties of $\Phi _{0 i}^{(j)}(x,t,k)$:

1. $\Phi _{0 1}^{(1)}(x,t,k)$ is analytic in $\mathbb {C}\setminus \{0\}$ and $$\Phi _{01}^{(1)}(x,t,k)=\begin{pmatrix} 1\\ 0 \end{pmatrix}+O(k)$$ as k→0 in $\mathbb {C}^-$.
2. $\Phi _{0 1}^{(2)}(x,t,k)$ is analytic in $\mathbb {C}\setminus \{0\}$ and $$\Phi _{0 1}^{(2)}(x,t,k)=\begin{pmatrix} 0\\ 1 \end{pmatrix}+O(k)$$ as k→0 in $\mathbb {C}^+$.
3. $\Phi _{0 2}^{(1)}(x,t,k)$ is analytic in $\mathbb {C}\setminus \{0\}$ and $$\Phi _{0 2}^{(1)}(x,t,k)=\begin{pmatrix} 1\\ 0 \end{pmatrix}+O(k)$$ as k→0 in $\mathbb {C}^+$.
4. $\Phi _{0 2}^{(2)}(x,t,k)$ is analytic in $\mathbb {C}\setminus \{0\}$ and $$\Phi _{0 2}^{(2)}(x,t,k)=\begin{pmatrix} 0\\ 1 \end{pmatrix}+O(k)$$ as k→0 in $\mathbb {C}^-$.
5. $\check{\Phi }_{0 3}^{(1)}(x,t,k)$ is analytic in $\mathbb {C}^+$ and continuous up to the boundary. Moreover, $\check{\Phi }_{0 3}^{(1)}(x,t,k)$=$$\begin{pmatrix} 1\\ 0 \end{pmatrix}+O(k)$$ as k→0 in $\mathbb {C}^+$.
6. $\check{\Phi }_{0 3}^{(2)}(x,t,k)$ is analytic in $\mathbb {C}^-$ and continuous up to the boundary. Moreover, $$\check{\Phi }_{03}^{(2)}(x,t,k)=\begin{pmatrix} 0\\ 1 \end{pmatrix}+O(k)$$ as k→0 in $\mathbb {C}^-$.

The properties (2.11) and (2.14) hold as well for $\Phi _{j}(x,t,k)$, $\Phi _{0 j}(x,t,k)$ for j=1,2, $\check{\Phi }_{3}(x,t,k)$ and $\check{\Phi }_{0 3}(x,t,k)$.

We define the matrices *s*(*k*), *S*(*k*), $\tilde{s}(k)$, and $\tilde{S}(k)$ as in (2.16), (2.17), (2.25) and (2.26), respectively, with $\Phi _{\infty 3}$ ($\Phi _{0 3}$) replaced by $\check{\Phi }_{3}$ ($\check{\Phi }_{0 3}$).

For Problem II, the spectral functions *a*(*k*), *b*(*k*), $\tilde{a}(k)$, and $\tilde{b}(k)$ possess the following properties:

(i) $\tilde{a}(k)$ and $\tilde{b}(k)$ are analytic in $\mathbb {C}^-$. Moreover, $\tilde{a}(0)=1$ and $\tilde{b}(0)=0$.
(ii) *a*(*k*) and *b*(*k*) are analytic in $\mathbb {C}^-$. Moreover, $a(k)=1+O(\frac{1}{k})$ and $b(k)=O(\frac{1}{k})$ as $k\rightarrow \infty$. Moreover, $a(0)=\kappa _1^0$ and $b(0)=\kappa _2^0$.

The relations (2.37), (2.40), (2.38), and (2.41) continue to hold, and the properties of *A*(*k*) and *B*(*k*) remain unchanged.

## Compatibility of initial and boundary values

In this section, we discuss the relations among the spectral functions.

### Problem I

First, we express $\Phi _{3}(0,T,k)$ in terms of $\Phi _{1}(0,T,k)$ using (2.15) and recall that $\Phi _{1}(0,T,k)=I$:

3.1 $$\begin{aligned} \Phi _{3}(0,T,k)=\textrm{e}^{-\textrm{i}kp(0,T,k)\sigma _3}S^{-1}(k)s(k)\textrm{e}^{\textrm{i}kp(0,T,k)\sigma _3}. \end{aligned}$$

Taking into account that $\Phi _{3}(0,T,k)$ can be estimated from

$$\begin{aligned} \Phi _{3}(0,T,k)=I- \int _{0}^{+\infty } \textrm{e}^{\textrm{i}ky\hat{\sigma }_3}( U_\infty \Phi _{3})(y,T,k) \textrm{d}y, \end{aligned}$$

we conclude that $\Phi ^{(12)}_{\infty 3}(0,T,k)=(\frac{1}{k})$ as $k\rightarrow \infty$ in $\mathbb {C}^+$. Then, considering the (12) entry in (3.1), we arrive at the following asymptotic relationship among the spectral functions:

3.2 $$\begin{aligned} A(k)b(k)-B(k)a(k)=O(\frac{1}{k}),\quad k\rightarrow \infty , \quad k\in \mathbb {C}^+. \end{aligned}$$

Relationships of this type (known as “Global Relations”) arise when analyzing IBV problems by using the RH method, and often they are restrictions on the spectral functions that reflect the dependence between initial and boundary problems (see [8–10]). In this respect, we notice that the relation (3.2) provides no additional restriction on the behavior of the corresponding spectral functions, since it follows from the properties of *a*(*k*), *b*(*k*), *A*(*k*), and *B*(*k*) listed in Sect. 2.

Similarly, the relations (2.24) yield

3.3 $$\begin{aligned} \Phi _{03}(0,T,k)=\textrm{e}^{-\textrm{i}k p(0,T,k)\sigma _3}\tilde{S}^{-1}(k)\tilde{s}(k)\textrm{e}^{\textrm{i}kp(0,T,k)\sigma _3}. \end{aligned}$$

In particular, its (12) entry takes the form

$$\begin{aligned} \Phi ^{(12)}_{03}(0,T,k)=(\tilde{A}(k)\tilde{b}(k)-\tilde{B}(k)\tilde{a}(k))\textrm{e}^{\frac{\textrm{i}}{2k}T}, \end{aligned}$$

and, recalling that $\Phi ^{(12)}_{03}(0,T,k)=O(k)$ as k→0, we arrive at the following asymptotic relation:

3.4 $$\begin{aligned} (\tilde{A}(k)\tilde{b}(k)-\tilde{B}(k)\tilde{a}(k))\textrm{e}^{\frac{\textrm{i}}{2k}T}=O(k),\quad k\rightarrow 0 ,\quad k\in \mathbb {C}^+. \end{aligned}$$

In contrast with (3.2), the asymptotic formula (3.4) does present a restriction on the involved spectral functions, which reflects the compatibility of the initial and boundary data in the spectral terms.

### Problem II

By arguments analogous to those used for Problem I, we obtain

3.5 $$\begin{aligned} (A(k)b(k)-B(k)a(k))=O\bigg (\frac{1}{k}\bigg ),\quad k\rightarrow \infty , \quad k\in \mathbb {C}^- \end{aligned}$$

and

3.6 $$\begin{aligned} (\tilde{A}(k)\tilde{b}(k)-\tilde{B}(k)\tilde{a}(k))\textrm{e}^{\frac{\textrm{i}}{2k}T}=O(k),\quad k\rightarrow 0 ,\quad k\in \mathbb {C}^-. \end{aligned}$$

In this case, (3.5) and (3.6) present any additional restrictions on the spectral functions: They follow from the properties of *a*(*k*), *b*(*k*), $\tilde{a}(k)$, $\tilde{b}(k)$, *A*(*k*), *B*(*k*), $\tilde{A}(k)$, and $\tilde{B}(k)$ listed in Sect. 2.

## Inverse spectral mappings

In this section, the inverse spectral mappings are formulated through the solutions of associated Riemann–Hilbert problems, with jump matrices determined by the corresponding spectral functions.

Let ϵ>0 be sufficiently small so that all zeros of $\tilde{a}(k)$, $\tilde{a}^*(k)$, $\tilde{A}(k)$, and $\tilde{A}^*(k)$ appearing in the denominators below lie outside the disks $\{|k|\le \epsilon \}$.

### Problem I

#### The inverse *x*-spectral mapping

The inverse *x*-spectral mapping

$$\begin{aligned} \{ a(k),b(k) \}\longrightarrow \{ u_0(x) \} \end{aligned}$$

is based on solving a Riemann–Hilbert problem whose jump matrix is built from the given spectral functions *a* and *b*. The construction of this problem is suggested by the properties of $\Phi _{2}(x,0,k)$, $\Phi _{3}(x,0,k)$, $\Psi _{02}(x,0,k)$ and $\Psi _{03}(x,0,k)$ described in the direct spectral analysis.

first, notice that $\kappa _1^0$, $\kappa _2^0$ can be obtained by expanding *a*(*k*) and *b*(*k*) as k→0. Therefore, having *a*(*k*) and *b*(*k*), we can determine $\tilde{a}(k)$ and $\tilde{b}(k)$ via (2.29).

**Fig. 4:**
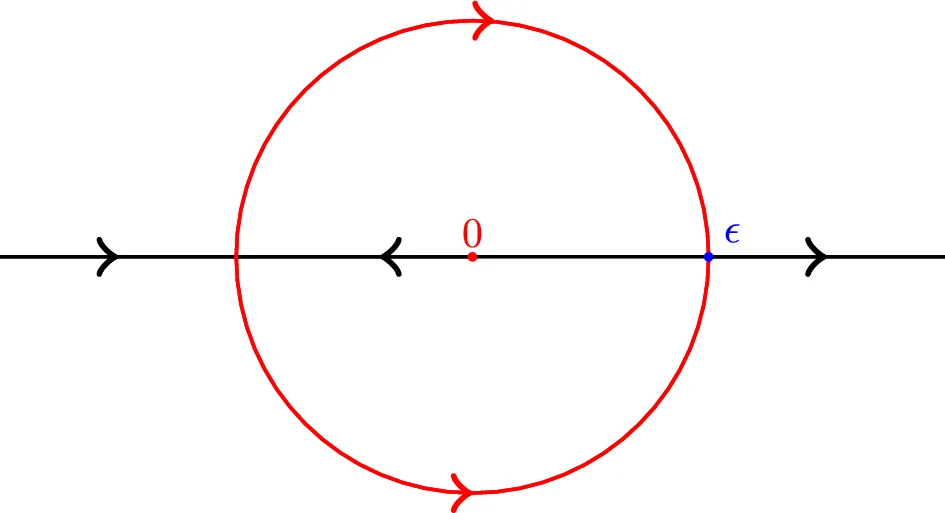
The oriented contour for the Riemann–Hilbert problems ${\textbf {RH}}^{(x)}$, ${\textbf {RH}}^{(t)}$, and ${\textbf {RH}}^{(xt)}$

Given $u_{0}(x)$, consider the matrix-valued function $M^{(x)}(x,k)$ defined in the domains separated by the contour shown in Fig. 4:

4.1 $$\begin{aligned} M^{(x)}(x,k)={\left\{ \begin{array}{ll} \left( \frac{\Phi _{ 2}^{(1)}(x,0,k)}{a(k)},\Phi _{ 3}^{(2)}(x,0,k)\right) ,\quad k\in \mathbb {C}^+\cap \{|k|>\epsilon \},\\ \left( \frac{\Psi _{0 2}^{(1)}(x,0,k)}{\tilde{a}(k)},\Psi _{0 3}^{(2)}(x,0,k)\right) ,\quad k\in \mathbb {C}^+\cap \{|k|<\epsilon \},\\ \left( \Phi _{ 3}^{(1)}(x,0,k),\frac{\Phi _{ 2}^{(2)}(x,0,k)}{a^*( k)}\right) ,\quad k\in \mathbb {C}^-\cap \{|k| >\epsilon \},\\ \left( \Psi _{0 3}^{(1)}(x,0,k),\frac{\Psi _{0 2}^{(2)}(x,0,k)}{\tilde{a}^*( k)}\right) ,\quad k\in \mathbb {C}^-\cap \{|k| <\epsilon \}, \end{array}\right. } \end{aligned}$$

where the functions $\Psi _{0j}$ are defined by

4.2 $$\begin{aligned} \Psi _{0j}(x,t,k):=P^{-1}(x,t)\Phi _{0j}(x,t,k). \end{aligned}$$

Here, the functions $\Phi _{2}(x,0,k)$, $\Phi _{3}(x,0,k)$, $\Phi _{02}(x,0,k)$, $\Phi _{03}(x,0,k)$ are to be understood as defined by (2.9), (2.10), (2.22), and (2.23), respectively, for t=0, where *u*(*x*, 0) in $U_\infty (x,0)$ and $U_0(x,0)$ is replaced by $u_0(x)$.

Notice the following properties of $M^{(x)}(x,k)$:

1. Jump relation across $\mathbb {R}\cup \{|k|=\epsilon \}$:
  4.3a $$\begin{aligned} M_-^{(x)}(x,k)=M_+^{(x)}(x,k)J(x,k),\quad k\in \mathbb {R}\cup \{|k|=\epsilon \}, \end{aligned}$$
  where
  4.3b $$\begin{aligned} J(x,k)=\textrm{e}^{-\textrm{i}k x\sigma _3} J_0(k)\textrm{e}^{\textrm{i}k x\sigma _3} \end{aligned}$$
  with
  4.3c $$\begin{aligned} J_0(k)={\left\{ \begin{array}{ll} \begin{pmatrix} 1& -r^*(k)\\ -r(k)& 1+r(k)r^*(k) \end{pmatrix},\quad k\in \mathbb {R}\cap \{|k| >\epsilon \},\\ \begin{pmatrix} 1+\tilde{r}(k)\tilde{r}^*(k)& \tilde{r}^*(k)\\ \tilde{r}(k)& 1 \end{pmatrix},\quad k\in \mathbb {R}\cap \{|k| <\epsilon \},\\ \begin{pmatrix} 1& 0\\ -\frac{\kappa _2^0}{a(k)\tilde{a}(k)}& 1 \end{pmatrix},\quad k\in \mathbb {C}^+\cap \{|k| =\epsilon \},\\ \begin{pmatrix} 1& -\frac{\kappa _2^0}{a^*(k)\tilde{a}^*(k)}\\ 0& 1 \end{pmatrix},\quad k\in \mathbb {C}^-\cap \{|k| =\epsilon \}, \end{array}\right. } \end{aligned}$$
  where $$r(k)=\frac{b^*(k)}{a(k)}$$ and $$\tilde{r}(k)=\frac{\tilde{b}^*(k)}{\tilde{a}(k)}$$.
2. Behavior at ∞:
  4.4a $$\begin{aligned} M^{(x)}(x,k)=I+\frac{1}{4\textrm{i}k}M^{\infty }(x)+O(\frac{1}{k^2}),\quad k\rightarrow \infty \end{aligned}$$
  with
  4.4b $$\begin{aligned} M^{\infty }(x)=\begin{pmatrix} -\frac{1}{2}\int _x^\infty u_{0x}^2(y)\textrm{d}y& u_{0x}(x)\\ u_{0x}(x)& \frac{1}{2}\int _x^\infty u_{0x}^2(y)\textrm{d}y\end{pmatrix}. \end{aligned}$$

##### Proof

First, consider $k\in \mathbb {C}^+$. Expanding $\Phi _{2}^{(1)}(x,0, k)$ as $k\rightarrow \infty$ via the Neumann series and using integration by parts yields

$$ \Phi _{2}^{(1)}(x,0, k)=\begin{pmatrix} 1\\ 0 \end{pmatrix}+\frac{1}{4\textrm{i}k}\begin{pmatrix} \frac{1}{2}\int _0^x u_{0x}^2(y)\textrm{d}y\\ u_{0x}(x) \end{pmatrix}+O(\frac{1}{k^2}). $$

Similarly, expanding $\Phi _{03}^{(2)}(x,0, k)$ as $k\rightarrow \infty$, we obtain

$$ \Phi _{3}^{(2)}(x,0, k)=\begin{pmatrix} 0\\ 1 \end{pmatrix}+\frac{1}{4\textrm{i}k}\begin{pmatrix}u_{0x}(x) \\ \frac{1}{2}\int _x^\infty u_{0x}^2(y)\textrm{d}y \end{pmatrix}+O(\frac{1}{k^2}), $$

and, in particular,

$$ a(k)=1+\frac{1}{8\textrm{i}k}\int _0^\infty u_{0x}^2(y)\textrm{d}y+O(\frac{1}{k^2}) $$

Therefore, (4.4) follows for $k\in \mathbb {C}^+$. For $k\in \mathbb {C}^-$, this follows expanding $\Phi _{03}^{(1)}(x,0, k)$ and $\Phi _{2}^{(2)}(x,0, k)$. □

3. $$\det M^{(x)}(x,k)\equiv 1$$.
4. Symmetry properties:
  4.5 $$\begin{aligned} M^{(x)}(- k)=\overline{M^{(x)}(\bar{k})},\qquad M^{(x)}(-k)=\sigma _2M^{(x)}(k)\sigma _2. \end{aligned}$$
5. Residue properties. Let $\{k_j\}$ be zeros of *a*(*k*) in $\mathbb {C}^+$. Assume that they are all simple. Then
  4.6a $$\begin{aligned} {{\,\textrm{Res}\,}}_{k_j}M^{(x)(1)}(x,k)&=\frac{e^{2\textrm{i}k_j x}}{\dot{a}(k_j)b(k_j)}M^{(x)(2)}(x,k_j),\end{aligned}$$
  4.6b $$\begin{aligned} {{\,\textrm{Res}\,}}_{- k_j}M^{(x)(2)}(x,k)&=\frac{e^{2\textrm{i}k_j x}}{\dot{a}( k_j)b( k_j)}M^{(x)(1)}(x,- k_j) \end{aligned}$$
  due to the symmetry (2.38).
6. Behavior at 0:
  4.7 $$\begin{aligned} M^{(x)}(x,k)=P_0^{-1}(x)+O(k), \quad k\rightarrow 0, \end{aligned}$$
  where
  $$ P_0(x)=\begin{pmatrix} \cos \frac{u_0}{2} & \sin \frac{u_0}{2} \\ -\sin \frac{u_0}{2} &  \cos \frac{u_0}{2} \\ \end{pmatrix}. $$

##### Remark 4.1

In the case $u_{0}(0)=4\pi k$, the definition of $M^{(x)}(x,k)$ and the corresponding jump conditions simplify, in view of (2.31), to the following:

$$\begin{aligned} M_-^{(x)}(x,k)=M_+^{(x)}(x,k)J(x,k),\quad k\in \mathbb {R} , \end{aligned}$$

where

$$ M^{(x)}(x,k)={\left\{ \begin{array}{ll} \left( \frac{\Phi _{\infty 2}^{(1)}(x,0,k)}{a(k)},\Phi _{\infty 3}^{(2)}(x,0,k)\right) ,\quad k\in \mathbb {C}^+,\\ \left( \Phi _{\infty 3}^{(1)}(x,0,k),\frac{\Phi _{\infty 2}^{(2)}(x,0,k)}{a^*( k)}\right) ,\quad k\in \mathbb {C}^-, \end{array}\right. } $$

$$\begin{aligned} J(x,t,k)=\textrm{e}^{-\textrm{i}kx\sigma _3} J_0(k)\textrm{e}^{\textrm{i}kx\sigma _3} \end{aligned}$$

with

$$\begin{aligned} J_0( k)= \begin{pmatrix} 1& -r^*(k)\\ -r(k)& 1+r(k)r^*(k) \end{pmatrix},\quad k\in \mathbb {R}. \end{aligned}$$

Here $\mathbb {R}$ is oriented from left to right.

Now we notice that the properties of $M^{(x)}$ stated above suggest that $M^{(x)}(x,k)$ can be uniquely characterized as a solution of the RH problem (parametrized by *x*).

${\textbf {The Riemann--Hilbert problem RH}}^{(x)}$ **for** $M^{(x)}(x, k)$: Given *a*(*k*), *b*(*k*) for $k\in \mathbb {C}^+$ and the set $\{k_j\}$ in $\mathbb {C}^+$, find a piece-wise meromorphic 2×2 matrix-valued function $M^{(x)}(x, k)$ that satisfies the following conditions:

1. Jump condition (4.3).
2. Behavior at ∞:
  4.8 $$\begin{aligned} M^{(x)}(x, k)=I+O\left( \frac{1}{ k}\right) ,\quad k\rightarrow \infty . \end{aligned}$$
3. Residue conditions (4.6).

##### Proposition 4.2

If a solution $M^{(x)}$ of ${\textbf {RH}}^{(x)}$ exists, then:

1. it is unique;
2. $$\det M^{(x)}\equiv 1$$;
3. 4.9 $$\begin{aligned} M^{(x)}( k)=\overline{ M^{(x)}(-\bar{k})},\qquad M^{(x)}(k)=\sigma _2 \overline{ M^{(x)}(\bar{k})}\sigma _2. \end{aligned}$$

##### Proof

(2) The conditions for $$ M^{(x)}$$ ensure that $$\det M^{(x)}$$ has neither a jump across $$\mathbb {R}\cup \{|k|=\epsilon \}$$ no singularities at $$k_j$$. As $$\det M^{(x)}$$ tends to 1 as $$k \rightarrow \infty $$, Liouville’s theorem implies that $$\det M^{(x)}\equiv 1$$.

(1) The uniqueness of the solution follows by applying the Liouville-type arguments to the ratio $$ M_1^{(x)}( M_2^{(x)})^{-1}$$ of two potential solutions, $$ M_1^{(x)}$$ and $$ M_2^{(x)}$$.

(3) The symmetries are inherited from the corresponding symmetries of the jump matrix and the uniqueness of the solution.

□

The uniqueness of the solution to the Riemann–Hilbert problem $${\textbf {RH}}^{(x)}$$, together with the properties of $$ M^{(x)}(x,k)$$, yields the following procedure for the inverse mapping

$$ \{a(k),\, b(k)\} \longrightarrow \{u_0(x)\} $$

for the *x*-problem:

Step 1. Given *a*(*k*) and *b*(*k*), compute $\kappa _1^0$ and $\kappa _2^0$ through (2.42), and determine $\tilde{a}(k)$ and $\tilde{b}(k)$ through (2.29). With these data, formulate the Riemann–Hilbert problem ${\textbf {RH}}^{(x)}$;
Step 2. Solve the RH problem constructed in the previous step and evaluate its solution $$ M^{(x)}(x,k)$$ at k=∞ (cf. (4.4)):
  $$\begin{aligned} M^{(x)}(x, k)=I+\frac{1}{4\textrm{i}k}\begin{pmatrix} -\eta (x) &  \xi (x)\\ \xi (x) &  \eta (x) \end{pmatrix}+O\left( \frac{1}{k^2}\right) . \end{aligned}$$
Step 3. Determine $u_{0x}(x)$ as follows:
  4.10 $$\begin{aligned} u_{0x}(x)=\xi (x). \end{aligned}$$
Step 4. Having $u_{0x}(x)$, reconstruct $u_0(x)$ by
  4.11 $$\begin{aligned} u_0(x) = -\int _x^\infty u_{0x}(y)\textrm{d}y +2\pi k \end{aligned}$$

##### Remark 4.3

Alternatively, we can use (4.7) in order to reconstruct $$u_0(x)$$. More precisely, evaluate the solution $$ M^{(x)}(x, k)$$ of this RH problem at k=0

4.12 $$\begin{aligned} M^{(x)}(x, k) =\begin{pmatrix} \alpha (x)&  \beta (x)\\ - \beta (x)&  \alpha (x) \end{pmatrix}+O(k), \end{aligned}$$

and define $\sin \tfrac{u_0(x)}{2}$ and $\cos \tfrac{u_0(x)}{2}$ by

$$ \sin \tfrac{u_0(x)}{2}=\beta (x),\qquad \cos \tfrac{u_0(x)}{2}=\alpha (x). $$

Then

$$ \sin u_0(x)=2\alpha (x)\beta (x),\qquad \cos u_0(x)=\alpha ^2(x)-\beta ^2(x) $$

and

$$ u_{0x}=\cos u_0 (\sin u_0)_x-\sin u_0(\cos u_0)_x=-2\alpha \beta (\alpha +\beta )(\alpha _x+\beta _x). $$

#### The inverse *t*-spectral mapping

The inverse *t*-spectral mapping

$$\begin{aligned} \{ A(k), B(k) \}\longrightarrow \{ v_0(t) \} \end{aligned}$$

can be expressed in terms of the solution of a Riemann–Hilbert problem with a jump matrix determined by *A* and *B*. This RH problem is constructed using the relations among the eigenfunctions $\Phi _{ 1}(0,t,k)$, $\Phi _{ 2}(0,t, k)$, $\Psi _{01}(0,t, k)$, and $\Psi _{02}(0,t, k)$ arising in the direct spectral analysis.

Notice that having *A*(*k*) and *B*(*k*), we can obtain $\kappa _i^0$, and $\kappa _i^T$, i=1,2, and determine $\tilde{A}( k)$ and $\tilde{B}(k)$ (see (2.50) and (2.34)).

We introduce the matrix-valued function $M^{(t)}(t,k)$ defined in the domains separated by the contour shown in Fig. 4:

4.13 $$\begin{aligned} M^{(t)}(t, k)={\left\{ \begin{array}{ll} \left( \Phi _{2}^{(1)}(0,t, k),\frac{\Phi _{ 1}^{(2)}(0,t, k)}{A( k)}\right) ,\quad k\in \mathbb {C}^+\cap \{| k|>\epsilon \},\\ \left( \Psi _{0 2}^{(1)}(0,t, k),\frac{\Psi _{0 1}^{(2)}(0,t, k)}{\tilde{A}( k)}\right) ,\quad k\in \mathbb {C}^+\cap \{| k|<\epsilon \},\\ \left( \frac{\Phi _{ 1}^{(1)}(0,t, k)}{A^*( k)},\Phi _{ 2}^{(2)}(0,t, k)\right) ,\quad k\in \mathbb {C}^-\cap \{| k| >\epsilon \}\\ \left( \frac{\Psi _{0 1}^{(1)}(0,t, k)}{\tilde{A}^*( k)},\Psi _{0 2}^{(2)}(0,t, k)\right) ,\quad k\in \mathbb {C}^-\cap \{| k| <\epsilon \}, \end{array}\right. } \end{aligned}$$

where the functions $\Psi _{0j}$ are defined by (4.2).

Then, the function $M^{(t)}(t, k)$ has the following properties:

1. Jump relation across $\mathbb {R}\cup \{|k|=\epsilon \}$
  4.14a $$\begin{aligned} M_-^{(t)}(t, k)=M_+^{(t)}(t, k)J^{(t)}(t, k),\quad k\in \mathbb {R}\cup \{|k|=\epsilon \} \end{aligned}$$
  where
  4.14b $$\begin{aligned} J^{(t)}(t, k)=\textrm{e}^{\tfrac{\textrm{i}t}{4 k}\sigma _3} J^{(t)}_0( k)\textrm{e}^{-\tfrac{\textrm{i}t}{4 k}\sigma _3} \end{aligned}$$
  with
  4.14c $$\begin{aligned} J^{(t)}_0( k)={\left\{ \begin{array}{ll} \begin{pmatrix} 1+R( k)R^*( k)& -R( k)\\ -R^*( k)& 1 \end{pmatrix},\quad k\in \mathbb {R}\cap \{| k|>\epsilon \},\\ \begin{pmatrix} 1& \tilde{R}( k)\\ \tilde{R}^*( k)& 1+\tilde{R}( k)\tilde{R}^*( k) \end{pmatrix},\quad k\in \mathbb {R}\cap \{| k|<\epsilon \},\\ \begin{pmatrix} \kappa _1^0+R(k)\kappa _2^0& \tilde{R}(k)\kappa _1^0+\tilde{R}(k)R(k)\kappa _2^0+\kappa _2^0-R(k)\kappa _1^0\\ -\kappa _2^0 &  \kappa _1^0-\tilde{R}(k)\kappa _2^0 \end{pmatrix},\quad k\in \mathbb {C}^+\cap \{| k|=\epsilon \},\\ \begin{pmatrix} \kappa _1^0+\tilde{R}^*(k)\kappa _2^0& -\kappa _2^0\\ \tilde{R}^*(k)\kappa _1^0+\tilde{R}^*(k)R^*(k)\kappa _2^0+\kappa _2^0-R^*(k)\kappa _1^0& \kappa _1^0-R^*(k)\kappa _2^0 \end{pmatrix},\quad k\in \mathbb {C}^-\cap \{| k|=\epsilon \} \end{array}\right. } \end{aligned}$$
  and $$R( k):=\frac{B( k)}{A( k)}$$ and $$\tilde{R}( k):=\frac{\tilde{B}( k)}{\tilde{A}( k)}$$.
2. Behavior at ∞:
  4.15 $$\begin{aligned} M^{(t)}(t, k)=I+O\left( \frac{1}{ k}\right) ,\quad k\rightarrow \infty . \end{aligned}$$
3. $$\det M^{(t)}(t, k)\equiv 1$$
4. Symmetry properties:
  4.16 $$\begin{aligned} M^{(t)}( k)=\overline{M^{(t)}(-\bar{k})},\qquad M^{(t)}( k)=\sigma _2M^{(t)}( -k)\sigma _2 \end{aligned}$$
5. Residue properties: Let $\{\mu _j\}$ be zeros of *A*(*k*) in $\mathbb {C}^+$. Assume that they are all simple. Then, using the symmetries (2.45), it follows that
  4.17a $$\begin{aligned} {{\,\textrm{Res}\,}}_{\mu _j}M^{(t)(2)}(t, k)&=\frac{B(\mu _j)}{\dot{A}(\mu _j)}e^{\frac{\textrm{i}t}{2\mu _j}}M^{(t)(1)}(t,\mu _j),\end{aligned}$$
  4.17b $$\begin{aligned} {{\,\textrm{Res}\,}}_{-\mu _j}M^{(t)(1)}(t, k)&=\frac{B(\mu _j)}{\dot{A}(\mu _j)}e^{\frac{\textrm{i}t}{2\mu _j}}M^{(t)(2)}(t,-\mu _j). \end{aligned}$$
6. Assume that $v_0(t):=u(0,\cdot )\in W^{2,1}(0,T)$. Then expanding $\Phi _{02}^{(1)}(0,t, k)$ and $\Phi _{01}^{(2)}(0,t, k)$ at k=0 in $\mathbb {C}^+$ via the Neumann series using integration by parts yields
  $$ \Phi _{02}^{(1)}(0,t, k)=\begin{pmatrix} 1\\ 0 \end{pmatrix}+\textrm{i}k\begin{pmatrix} \frac{1}{2}\int _0^t u_{t}^2(0,\tau )\textrm{d}\tau \\ -u_t(0,t) \end{pmatrix}+O(k^2), $$
  and
  $$ \Phi _{01}^{(2)}(0,t, k)=\begin{pmatrix} 0\\ 1 \end{pmatrix}+\textrm{i}k\begin{pmatrix} -u_t(0,t) \\ \frac{1}{2}\int _t^T u_{t}^2(0,\tau )\textrm{d}\tau \end{pmatrix}+O(k^2). $$
  In particular,
  $$ A(k)=1+\frac{\textrm{i}k}{2}\int _0^T u_{t}^2(0,\tau )\textrm{d}\tau +O(k^2). $$
  Therefore,
  4.18a $$\begin{aligned} M^{(t)}(t,k)=P^{-1}(0,t)\left( I+\textrm{i}kM^{0}(t)+O(k^2)\right) ,\quad k\rightarrow 0, \quad k\in \mathbb {C}^+ \end{aligned}$$
  with
  4.18b $$\begin{aligned} M^{0}(t)=\begin{pmatrix} \frac{1}{2}\int _0^t u_{t}^2(0,\tau )\textrm{d}\tau & -u_t(0,t)\\ -u_t(0,t)& -\frac{1}{2}\int _0^t u_{t}^2(0,\tau )\textrm{d}\tau \end{pmatrix}. \end{aligned}$$
  Similarly, expanding $\Phi _{01}^{(1)}(0,t, k)$ and $\Phi _{02}^{(2)}(0,t, k)$ at k=0 in $\mathbb {C}^-$ yields (4.18a)–(4.18b) in $\mathbb {C}^-$.

##### Remark 4.4

In the case $u_{0}(0)=4\pi k$, the jump matrix simplifies to

4.19 $$\begin{aligned} J^{(t)}_0( k)={\left\{ \begin{array}{ll} \begin{pmatrix} 1+R( k)R^*( k)& -R( k)\\ -R^*( k)& 1 \end{pmatrix},\quad k\in \mathbb {R}\cap \{| k|>\epsilon \},\\ \begin{pmatrix} 1& \tilde{R}( k)\\ \tilde{R}^*( k)& 1+\tilde{R}( k)\tilde{R}^*( k) \end{pmatrix},\quad k\in \mathbb {R}\cap \{| k|<\epsilon \},\\ \begin{pmatrix} 1& \tilde{R}(k)-R(k)\\ 0 &  1 \end{pmatrix},\quad k\in \mathbb {C}^+\cap \{| k|=\epsilon \},\\ \begin{pmatrix} 1& 0\\ \tilde{R}^*(k)-R^*(k)& 1 \end{pmatrix},\quad k\in \mathbb {C}^-\cap \{| k|=\epsilon \} \end{array}\right. } \end{aligned}$$

Now we notice that the properties of $M^{(t)}$ stated above suggest that $M^{(t)}(t,k)$ can be uniquely characterized as a solution of the RH problem (parametrized by *t*).

${\textbf {The Riemann--Hilbert problem RH}} ^{(t)}$ **for** $M^{(t)}(t, k)$: Given *A*(*k*), *B*(*k*) for $k\in \mathbb {C}\setminus \{0\}$ and the set $\{\mu _j\}$ in $\mathbb {C}^+$, find a piece-wise meromorphic 2×2 matrix-valued function $M^{(t)}(t, k)$ that satisfies the following conditions:

1. Jump condition (4.14).
2. Behavior at ∞:
  4.20 $$\begin{aligned} M^{(t)}(t, k)=I+O\left( \frac{1}{ k}\right) ,\quad k\rightarrow \infty . \end{aligned}$$
3. Residue conditions (4.17).

Similarly to the *x*-problem, the following proposition holds:

##### Proposition 4.5

If a solution $$ M^{(t)}$$ of $${\textbf {RH}}^{(t)}$$ exists, then

1. it is unique.
2. $$\det M^{(t)}\equiv 1$$.
3. 4.21 $$\begin{aligned} M^{(t)}( k)=\overline{ M^{(t)}(-\bar{k})},\qquad M^{(t)}(k)=\sigma _2\overline{ M^{(t)}(\bar{k})}\sigma _2. \end{aligned}$$

The uniqueness of the solution of the Riemann–Hilbert problem ${\textbf {RH}}^{(t)}$ justifies the following procedure for the inverse mapping

$$ \{ A(k),\, B(k) \} \longrightarrow \{v_0(t)\} $$

for the *t*-problem:

Step 1. Given *A*(*k*) and *B*(*k*), compute $\kappa _i^0$, $\kappa _i^T$, i=1,2, and determine $\tilde{A}(k)$ and $\tilde{B}(k)$ via (2.30). Having these data, formulate the Riemann–Hilbert problem ${\textbf {RH}}^{(t)}$;
Step 2. Solve the RH problem constructed in the previous step and evaluate its solution $M^{(t)}(t, k)$ at k=0:
  4.22 $$\begin{aligned} M^{(t)}(t, k)=\begin{pmatrix} \alpha (t)&  \beta (t)\\ - \beta (t)&  \alpha (t) \end{pmatrix}\left( I+\textrm{i}k\begin{pmatrix} f_1(t)&  f_2(t)\\ f_2(t)& -f_1(t) \end{pmatrix}+O(k^2)\right) . \end{aligned}$$
Step 3. Define $$ v_{0t}(t)$$ from this expansions in the following way (cf. (4.18)):
  4.23 $$\begin{aligned} v_{0t}(t)=- f_2(t). \end{aligned}$$
Step 4. Reconstruct $v_0(t)$ by
  4.24 $$\begin{aligned} v_0(t) = v_0(0)+ \int _0^t v_{0t}(\tau )\textrm{d}\tau . \end{aligned}$$

##### Remark 4.6

We could also define $\sin \tfrac{v_0(t)}{2}$ and $\cos \tfrac{v_0(t)}{2}$ by

$$ \sin \tfrac{v_0(t)}{2}=\beta (t),\qquad \cos \tfrac{v_0(t)}{2}=\alpha (t). $$

Then

$$ \sin v_0(t)=2\alpha (t)\beta (t),\qquad \cos v_0(t)=\alpha ^2(t)-\beta ^2(t). $$

and

$$ v_{0t}=\cos v_0 (\sin v_0)_t-\sin v_0(\cos v_0)_t=-2\alpha \beta (\alpha +\beta )(\alpha _t+\beta _t). $$

### Problem II

#### The inverse *x*-spectral mapping

Taking into account the analytic properties of the eigenfunctions and the scattering coefficients, we introduce the matrix-valued function $\check{M}^{(x)}(x,k)$ defined in the domains separated by the contour shown in Fig. 4:

4.25 $$\begin{aligned} \check{M}^{(x)}(x,k)={\left\{ \begin{array}{ll} \left( \check{\Phi }_{3}^{(1)}(x,0,k), \frac{\Phi _{ 2}^{(2)}(x,0,k)}{a^*(k)}\right) ,\quad k\in \mathbb {C}^+\cap \{| k|>\epsilon \},\\ \left( \check{\Psi }_{0 3}^{(1)}(x,0,k), \frac{\Psi _{0 2}^{(2)}(x,0,k)}{\tilde{a}^*(k)}\right) ,\quad k\in \mathbb {C}^+\cap \{| k|<\epsilon \},\\ \left( \frac{\Psi _{0 2}^{(1)}(x,0,k)}{\tilde{a}( k)},\check{\Psi }_{0 3}^{(2)}(x,0,k)\right) ,\quad k\in \mathbb {C}^-\cap \{| k| <\epsilon \},\\ \left( \frac{\Phi _{ 2}^{(1)}(x,0,k)}{a( k)},\check{\Phi }_{3}^{(2)}(x,0,k)\right) ,\quad k\in \mathbb {C}^-\cap \{| k| >\epsilon \}. \end{array}\right. } \end{aligned}$$

Then, the function $\check{M}^{(x)}(x,k)$ has the following properties:

1. Jump relation across $\mathbb {R}\cup \{|k|=\epsilon \}$:
  4.26a $$\begin{aligned} \check{M}_-^{(x)}(x,k)=\check{M}_+^{(x)}(x,k)\check{J}(x,k),\quad k\in \mathbb {R}\cup \{|k|=\epsilon \} \end{aligned}$$
  where
  4.26b $$\begin{aligned} \check{J}(x,k)=\textrm{e}^{-\textrm{i}kx\sigma _3} \check{J}_0(k)\textrm{e}^{\textrm{i}kx\sigma _3} \end{aligned}$$
  with
  4.26c $$\begin{aligned} \check{J}_0(k)={\left\{ \begin{array}{ll} \begin{pmatrix} 1+r(k)r^*(k)& r^*(k)\\ r(k)& 1 \end{pmatrix},\quad k\in \mathbb {R}\cap \{|k| >\epsilon \},\\ \begin{pmatrix} 1 & -\tilde{r}^*(k)\\ -\tilde{r}(k)&  1+\tilde{r}(k)\tilde{r}^*(k) \end{pmatrix},\quad k\in \mathbb {R}\cap \{|k| <\epsilon \},\\ \begin{pmatrix} 1& \frac{\kappa _2^0}{a^*(k)\tilde{a}^*(k)}\\ 0& 1 \end{pmatrix} ,\quad k\in \mathbb {C}^+\cap \{|k| =\epsilon \},\\ \begin{pmatrix} 1& 0\\ \frac{\kappa _2^0 }{a(k)\tilde{a}(k)}& 1 \end{pmatrix},\quad k\in \mathbb {C}^-\cap \{|k| =\epsilon \}, \end{array}\right. } \end{aligned}$$
  where $$r(k)=\frac{b^*(k)}{a(k)}$$ and $$\tilde{r}(k)=\frac{\tilde{b}^*(k)}{\tilde{a}(k)}$$.
2. Behavior at ∞:
  4.27a $$\begin{aligned} \check{M}^{(x)}(x,k)=I+\frac{1}{4\textrm{i}k}\check{M}^{\infty }(x)+O(\frac{1}{k^2}),\quad k\rightarrow \infty \end{aligned}$$
  with
  4.27b $$\begin{aligned} \check{M}^{\infty }(x)=\begin{pmatrix} \frac{1}{2}\int _{-\infty }^x u_{0x}^2(y)\textrm{d}y&  \quad u_{0x}(x)\\ u_{0x}(x)&  \quad - \frac{1}{2}\int _{-\infty }^x u_{0x}^2(y)\textrm{d}y\end{pmatrix}. \end{aligned}$$
3. $$\det \check{M}^{(x)}(x,k)\equiv 1$$
4. Symmetry properties:
  4.28 $$\begin{aligned} \check{M}^{(x)}(- k)=\overline{\check{M}^{(x)}(\bar{k})},\qquad \check{M}^{(x)}(-k)=\sigma _2\check{M}^{(x)}(k)\sigma _2 \end{aligned}$$
5. Residue properties. Let $\{k_j\}$ be zeros of *a*(*k*) in $\mathbb {C}^-$. Assume that they are all simple. Then
  4.29a $$\begin{aligned} {{\,\textrm{Res}\,}}_{k_j}\check{M}^{(x)(1)}(x,k)&=\frac{e^{2\textrm{i}k_jx}}{\dot{a}(k_j)b(k_j)}\check{M}^{(x)(2)}(x,k_j),\end{aligned}$$
  4.29b $$\begin{aligned} {{\,\textrm{Res}\,}}_{- k_j}\check{M}^{(x)(2)}(x,k)&=\frac{e^{2\textrm{i}k_j x}}{\dot{a}( k_j)b( k_j)}\check{M}^{(x)(1)}(x,- k_j). \end{aligned}$$
6. Behavior at 0:
  4.30 $$\begin{aligned} \check{M}^{(x)}(x,k)=\check{P}_0(x)+O(k) , \quad k\rightarrow 0, \end{aligned}$$
  where
  $$ \check{P}_0(x)=\begin{pmatrix} \cos \frac{u_0}{2} &  \sin \frac{u_0}{2} \\ -\sin \frac{u_0}{2} &  \cos \frac{u_0}{2} \\ \end{pmatrix}. $$

##### Remark 4.7

In the case $u_{0}(0)=4\pi k$, the definition of $M^{(x)}(x,k)$ and the corresponding jump conditions simplify, in view of (2.31), to the following:

$$\begin{aligned} \check{M}_-^{(x)}(x,k)=\check{M}_+^{(x)}(x,k)\check{J}(x,k),\quad k\in \mathbb {R} \end{aligned}$$

where

$$ \check{M}^{(x)}(x,k)={\left\{ \begin{array}{ll} \left( \check{\Phi }_{3}^{(1)}(x,0,k), \frac{\Phi _{ 2}^{(2)}(x,0,k)}{a^*(k)}\right) ,\quad k\in \mathbb {C}^+,\\ \left( \frac{\Phi _{ 2}^{(1)}(x,0,k)}{a( k)},\check{\Phi }_{3}^{(2)}(x,0,k),\right) ,\quad k\in \mathbb {C}^-. \end{array}\right. } $$

$$\begin{aligned} \check{J}(x,k)=\textrm{e}^{-\textrm{i}kx\sigma _3} J_0^{(x)}(k)\textrm{e}^{\textrm{i}kx\sigma _3} \end{aligned}$$

with

4.31 $$\begin{aligned} \check{J}^{(x)}_0( k)= \begin{pmatrix} 1+r(k)r^*(k)& r^*(k)\\ r(k)& 1 \end{pmatrix},\quad k\in \mathbb {R}. \end{aligned}$$

Here $\mathbb {R}$ is oriented from left to right.

Now we notice that the properties of $\check{M}^{(x)}$ stated above suggest that $\check{M}^{(x)}(x,k)$ can be uniquely characterized as a solution of the RH problem (parametrized by *x*).

**The Riemann–Hilbert problem**  **for** $\check{M}^{(x)}(x, k)$: Given *a*(*k*), *b*(*k*) for $k\in \mathbb {C}^+$ and the set $\{k_j\}$ in $\mathbb {C}^+$, find a piece-wise meromorphic 2×2 matrix-valued function $\check{M}^{(x)}(x, k)$ that satisfies the following conditions:

1. Jump condition (4.26).
2. Behavior at ∞:
  4.32 $$\begin{aligned} \check{M}^{(x)}(x, k)=I+O\left( \frac{1}{ k}\right) ,\quad k\rightarrow \infty . \end{aligned}$$
3. Residue conditions (4.29).

Similarly to Problem I, the proposition similar to Proposition 4.2 holds for , which justifies the following procedure of the inverse mapping:

Step 1. Given *a*(*k*) and *b*(*k*), compute $\kappa _1^0$ and $\kappa _2^0$ through (2.42), and determine $\tilde{a}(k)$ and $\tilde{b}(k)$ through (2.29). With these data, formulate the Riemann–Hilbert problem ;
Step 2. Solve the RH problem constructed in the previous step and evaluate its solution $\check{M}^{(x)}(x,k)$ at k=∞ (c.f. (4.27)):
  $$\begin{aligned} \check{M}^{(x)}(x, k)=I+\frac{1}{4\textrm{i}k}\begin{pmatrix} -\eta (x) &  \xi (x)\\ \xi (x) &  \eta (x) \end{pmatrix}+O\left( \frac{1}{k^2}\right) . \end{aligned}$$
Step 3. Determine $u_{0x}(x)$ as follows:
  4.33 $$\begin{aligned} u_{0x}(x)=\xi (x). \end{aligned}$$
Step 4. Having $u_{0x}(x)$, reconstruct $u_0(x)$ by
  4.34 $$\begin{aligned} u_0(x) = \int _{-\infty }^x u_{0x}(y)\textrm{d}y +2\pi k. \end{aligned}$$

For an alternative way to reconstruct $u_0(x)$, see Remark 4.3.

#### The inverse *t*-spectral mapping

Naturally, Problems I and II share the same *t*-spectral mapping.

## The master Riemann–Hilbert problems

### Problem I

Assume that Problem I (1.2) admits a solution *u*(*x*, *t*). Using suitable solutions of the Lax pair equations, we define the piece-wise (with respect to the contour shown in Fig. 4) meromorphic matrix-valued function *M*(*x*, *t*, *k*), depending on (*x*, *t*) as parameters:

5.1 $$\begin{aligned} M^{(xt)}(x,t,k)={\left\{ \begin{array}{ll} \left( \frac{\Phi _{ 2}^{(1)}(x,t,k)}{a(k)},\Phi _{ 3}^{(2)}(x,t,k)\right) ,\quad k\in \mathbb {C}^+\cap \{|k|>\epsilon \},\\ \left( \frac{\Psi _{0 2}^{(1)}(x,t,k)}{\tilde{a}(k)},\Psi _{0 3 }^{(2)}(x,t,k)\right) ,\quad k\in \mathbb {C}^+\cap \{|k|<\epsilon \},\\ \left( \Phi _{ 3}^{(1)}(x,t,k),\frac{\Phi _{ 2}^{(2)}(x,t,k)}{a^*( k)}\right) ,\quad k\in \mathbb {C}^-\cap \{|k| >\epsilon \},\\ \left( \Psi _{0 3}^{(1)}(x,t,k),\frac{\Psi _{0 2}^{(2)}(x,t,k)}{\tilde{a}^*( k)}\right) ,\quad k\in \mathbb {C}^-\cap \{|k| <\epsilon \}, \end{array}\right. } \end{aligned}$$

where the functions $\Psi _{0j}$ are defined by (4.2).

The function $M^{(xt)}(x,t,k)$ possesses the following properties:

1. Jump relation across $\mathbb {R}\cup \{|k|=\epsilon \}$
  5.2a $$\begin{aligned} M_-^{(xt)}(x,t,k)=M_+^{(xt)}(x,t,k)J^{(xt)}(x,t,k),\quad k\in \mathbb {R}\cup \{|k|=\epsilon \} \end{aligned}$$
  where
  5.2b $$\begin{aligned} J^{(xt)}(x,t,k)=\textrm{e}^{-\textrm{i}kp(x,t,k)\sigma _3} J^{(xt)}_0(k)\textrm{e}^{\textrm{i}kp(x,t,k)\sigma _3} \end{aligned}$$
  with $$p(x,t,k)=x-\frac{t}{4 k^2}$$ and
  5.2c $$\begin{aligned} J^{(xt)}_0(k)={\left\{ \begin{array}{ll} \begin{pmatrix} 1& -r^*(k)\\ -r(k)& 1+r(k)r^*(k) \end{pmatrix},\quad k\in \mathbb {R}\cap \{|k| >\epsilon \},\\ \begin{pmatrix} 1+\tilde{r}(k)\tilde{r}^*(k)& \tilde{r}^*(k)\\ \tilde{r}(k)& 1 \end{pmatrix},\quad k\in \mathbb {R}\cap \{|k| <\epsilon \},\\ \begin{pmatrix} 1& 0\\ -\frac{\kappa _2^0}{a(k)\tilde{a}(k)}& 1 \end{pmatrix},\quad k\in \mathbb {C}^+\cap \{|k| =\epsilon \},\\ \begin{pmatrix} 1& -\frac{\kappa _2^0}{a^*(k)\tilde{a}^*(k)}\\ 0& 1 \end{pmatrix},\quad k\in \mathbb {C}^-\cap \{|k| =\epsilon \} \end{array}\right. } \end{aligned}$$
  and $r(k)=\frac{b^*(k)}{a(k)}$, $\tilde{r}(k)=\frac{\tilde{b}^*(k)}{\tilde{a}(k)}$. Notice that (2.29) implies
  $$ \tilde{r}(k)=r(k)-\frac{\kappa _2 }{a(k)\tilde{a}(k)}, \quad k\in \mathbb {R}.$$
2. Behavior at ∞:
  5.3 $$\begin{aligned} M^{(xt)}(x,t,k)=I+\frac{1}{4\textrm{i}k}\begin{pmatrix} *& u_{x}(x,t)\\ u_{x}(x,t)& *\end{pmatrix}+O\bigg (\frac{1}{k^2}\bigg ),\quad k\rightarrow \infty . \end{aligned}$$
3. $$\det M^{(xt)}(x,t,k)\equiv 1$$
4. Symmetry properties:
  5.4 $$\begin{aligned} M^{(xt)}( k)=\overline{M^{(xt)}(-\bar{k})},\qquad M^{(xt)}(k)=\sigma _2 \overline{M^{(xt)}(\bar{k})}\sigma _2 \end{aligned}$$
5. Residue properties. Let $\{k_j\}$ be zeros of *a*(*k*) in $\mathbb {C}^+$. Assume that they are all simple. Then
  5.5a $$\begin{aligned} {{\,\textrm{Res}\,}}_{k_j}M^{(xt)(1)}(x,t,k)&=\frac{e^{2\textrm{i}k_jp(x,t,k_j)}}{\dot{a}(k_j)b(k_j)}M^{(xt)(2)}(x,t,k_j),\end{aligned}$$
  5.5b $$\begin{aligned} {{\,\textrm{Res}\,}}_{- k_j}M^{(xt)(2)}(x,t,k)&=\frac{e^{2\textrm{i}k_jp(x,t, k_j)}}{\dot{a}( k_j)b( k_j)}M^{(xt)(1)}(x,t,- k_j). \end{aligned}$$
6. Behavior at 0:
  5.6 $$\begin{aligned} M^{(xt)}(x,t,k)=P^{-1}(x,t)\left( I-\textrm{i}k\begin{pmatrix} *& u_t(x,t)\\ u_t(x,t)&  * \end{pmatrix}+O(k^2)\right) .\end{aligned}$$

The properties of $M^{(xt)}$ stated above (that follow from the analysis of the direct problem) suggest formulating a Riemann–Hilbert factorization problem parametrized by (*x*, *t*).

${\textbf {The Riemann--Hilbert problem RH}}^{(xt)}$ **for** $M^{(xt)}(x,t,k)$: Given *a*(*k*), *b*(*k*) for $k\in \overline{\mathbb {C}^+}$, and the set $\{k_j\}_1^N\subset \mathbb {C}^+$, find a piece-wise meromorphic 2×2 matrix-valued function $M^{(xt)}(x,t,k)$ that satisfies the following conditions:

1. Jump condition (5.2) across $\mathbb {R}\cup |k|=\epsilon$.
2. Behavior at ∞:
  5.7 $$\begin{aligned} M^{(xt)}(x, t,k)=I+O\left( \frac{1}{ k}\right) ,\quad k\rightarrow \infty . \end{aligned}$$
3. Residue conditions (5.5).

#### Proposition 5.1

There exists a unique solution $M^{(xt)}(x,t,k)$ of the RH problem ${\textbf {RH}}^{(xt)}$ for all *x* and *t*. Moreover, $\det M^{(xt)}\equiv 1$ and

5.8 $$\begin{aligned} M^{(xt)}( k)=\overline{ M^{(xt)}(-\bar{k})},\qquad M^{(xt)}(k)=\sigma _2\overline{ M^{(xt)}(\bar{k})}\sigma _2 \end{aligned}$$

Concerning the proof, notice that the jump matrix $$ J^{(xt)}(x,t, k)$$ defined in (5.2b)–(5.2c) is positively defined on $$\mathbb {R}$$ and satisfies the symmetry

$$\begin{aligned} J^{(xt)}(x, t,k)=\overline{ J^{(xt)T}(x,t,\bar{k})}, \qquad k\in \{|k|=\epsilon \}. \end{aligned}$$

These properties allow us to invoke Zhou’s vanishing lemma (see [21]), which ensures the solvability of the Riemann–Hilbert problem ${\textbf {RH}}^{(xt)}$.

#### Remark 5.2

The construction of the master RH problem ${\textbf {RH}}^{(xt)}$ uses the same data as ${\textbf {RH}}^{(x)}$, i.e., the scattering functions associated with the initial condition alone.

#### Remark 5.3

In the case $u_{0}(0)=4\pi k$, ${\textbf {RH}}^{(xt)}$ simplifies to the following:

1. Jump condition
  $$\begin{aligned} M_-^{(xt)}(x,t,k)=M_+^{(xt)}(x,t,k)J(x,t,k),\quad k\in \mathbb {R} \end{aligned}$$
  where
  $$\begin{aligned} J(x,t,k)=\textrm{e}^{-\textrm{i}kp(x,t,k)\sigma _3} J_0(k)\textrm{e}^{\textrm{i}kp(x,t,k)\sigma _3} \end{aligned}$$
  with
  $$\begin{aligned} J_0( k)= \begin{pmatrix} 1& -r^*(k)\\ -r(k)& 1+r(k)r^*(k) \end{pmatrix},\quad k\in \mathbb {R}. \end{aligned}$$
  Here $\mathbb {R}$ is oriented from left to right.
2. Behavior at ∞:
  $$\begin{aligned} M^{(xt)}(x, t,k)=I+O\left( \frac{1}{ k}\right) ,\quad k\rightarrow \infty . \end{aligned}$$
3. Residue conditions (5.5).

### Problem II

Assume that Problem II (1.3) admits a solution $u\in C^1([0,T],\tilde{H}^{1,2}((-\infty ,0)))$.

Introduce

$$\begin{aligned} d(k)=a(k)A^*(k)+b(k)B^*(k), \quad \tilde{d}(k)=\tilde{a}(k)\tilde{A}^*(k)+\tilde{b}(k)\tilde{B}^*(k), \end{aligned}$$

and define the piece-wise meromorphic matrix-valued function *M*(*x*, *t*, *k*), depending on (*x*, *t*) as parameters, in the domains separated by the contour shown in Fig. 4:

5.9 $$\begin{aligned} \check{M}^{(xt)}(x,t,k)={\left\{ \begin{array}{ll} \left( \check{\Phi }_{3}^{(1)}(x,t,k),\frac{\Phi _{ 1}^{(2)}(x,t,k)}{d^*(k)}\right) ,\quad k\in \mathbb {C}^+\cap \{|k|>\epsilon \},\\ \left( \check{\Psi }_{0 3}^{(1)}(x,t,k),\frac{\Psi _{0 1}^{(2)}(x,t,k)}{\tilde{d}^*(k)}\right) ,\quad k\in \mathbb {C}^+\cap \{|k|<\epsilon \},\\ \left( \frac{\Phi _{ 1}^{(1)}(x,t,k)}{d(k)},\check{\Phi }_{3}^{(2)}(x,t,k)\right) ,\quad k\in \mathbb {C}^-\cap \{|k| >\epsilon \},\\ \left( \frac{\Psi _{0 1}^{(1)}(x,t,k)}{\tilde{d}(k)},\check{\Psi }_{0 3}^{(2)}(x,t,k)\right) ,\quad k\in \mathbb {C}^-\cap \{|k| <\epsilon \}, \end{array}\right. } \end{aligned}$$

where the functions $\Psi _{0j}$ are defined by (4.2).

The function $\check{M}^{(xt)}(x,t,k)$ possesses the following properties:

1. Jump relation across $\mathbb {R}\cup \{|k|=\epsilon \}$:
  5.10a $$\begin{aligned} \check{M}_-^{(xt)}(x,t,k)=\check{M}_+^{(xt)}(x,t,k)\check{J}^{(xt)}(x,t,k),\quad k\in \Sigma , \end{aligned}$$
  where
  5.10b $$\begin{aligned} \check{J}^{(xt)}(x,t,k)=\textrm{e}^{-\textrm{i}kp(x,t,k)\sigma _3} \check{J}^{(xt)}_0(k)\textrm{e}^{\textrm{i}kp(x,t,k)\sigma _3} \end{aligned}$$
  with
  5.10c $$\begin{aligned} \check{J}^{(xt)}_0(k)={\left\{ \begin{array}{ll} \begin{pmatrix} 1+\rho (k)\rho ^*(k)& -\rho (k)\\ -\rho ^*(k)& 1 \end{pmatrix},\quad k\in \mathbb {R}\setminus \{|k|<\epsilon \},\\ \begin{pmatrix} 1& \tilde{\rho }(k)\\ \tilde{\rho }^*(k)&  1+\tilde{\rho }(k)\tilde{\rho }^*(k) \end{pmatrix},\quad k\in \mathbb {R}\cap \{|k|<\epsilon \},\\ \begin{pmatrix} {1}& \Xi (k)\\ 0& {1} \end{pmatrix},\quad k\in \mathbb {C}^+\cap \{|k|=\epsilon \},\\ \begin{pmatrix} {1}& 0\\ \Xi ^*(k)& {1} \end{pmatrix},\quad k\in \mathbb {C}^-\cap \{|k|=\epsilon \}, \end{array}\right. } \end{aligned}$$
  where
  5.11 $$\begin{aligned}&\rho (k):= \frac{a(k)B(k)-b(k)A(k)}{d^*(k)},\end{aligned}$$
  5.12 $$\begin{aligned}&\tilde{\rho }(k):= \frac{\tilde{a}(k)\tilde{B}(k)-\tilde{b}(k)\tilde{A}(k)}{\tilde{d}^*(k)}, \end{aligned}$$
  5.13 $$\begin{aligned}&\Xi (k):=\frac{\kappa _1^0(A(k)\tilde{B}(k)-B(k)\tilde{A}(k))+\kappa _2^0(B(k) \tilde{B}(k) +A(k) \tilde{A}(k))}{\tilde{d}^*(k) d^*(k)}. \end{aligned}$$
  Notice that
  $$ \rho (k)-\tilde{\rho }(k)+\Xi (k)=0. $$
2. Behavior at ∞:
  5.14 $$\begin{aligned} \check{M}^{(xt)}(x,t,k)=I+\frac{1}{4\textrm{i}k}\begin{pmatrix} *& u_{x}(x,t)\\ u_{x}(x,t)& *\end{pmatrix}+O\bigg (\frac{1}{k^2}\bigg ),\quad k\rightarrow \infty . \end{aligned}$$
3. $$\det \check{M}^{(xt)}(x,t,k)\equiv 1$$
4. Symmetry properties:
  5.15 $$\begin{aligned} \check{M}^{(xt)}(- k)=\overline{\check{M}^{(xt)}(\bar{k})},\qquad \check{M}^{(xt)}(k)=\sigma _2\overline{\check{M}^{(xt)}(\bar{k})}\sigma _2 \end{aligned}$$
5. Residue properties. Let $\{\eta _j\}$ be zeros of *d*(*k*) in $\mathbb {C}^-$. Assume that they are all simple. Then
  5.16a $$\begin{aligned} {{\,\textrm{Res}\,}}_{\eta _j}\check{M}^{(xt)(1)}(x,t,k)&=-\frac{e^{2\textrm{i}\eta _jp(x,t,\eta _j)}B^*(\eta _j)}{\dot{d}(\eta _j)a(\eta _j)}\check{M}^{(xt)(2)}(x,t,\eta _j), \end{aligned}$$
  5.16b $$\begin{aligned} {{\,\textrm{Res}\,}}_{- \eta _j}\check{M}^{(xt)(2)}(x,t,k)&=\frac{e^{2\textrm{i}\eta _jp(x,t, \eta _j)}B^*(\eta _j)}{\dot{d}( \eta _j)a( \eta _j)}\check{M}^{(xt)(1)}(x,t,- \eta _j). \end{aligned}$$
6. Behavior at 0:
  5.17 $$\begin{aligned} \check{M}^{(xt)}(x,t,k)=P^{-1}(x,t)\left( I-\textrm{i}k\begin{pmatrix} *& u_t(x,t)\\ u_t(x,t)&  * \end{pmatrix}+O(k^2)\right) .\end{aligned}$$

#### Remark 5.4

In the case u(0,0)=4πk, the matrix $\check{J}_0^{(xt)}(k)$ simplifies to the following:

5.18 $$\begin{aligned} \check{J}^{(xt)}_0(k)={\left\{ \begin{array}{ll} \begin{pmatrix} \frac{1}{dd^*}& -\frac{aB-bA}{d^*}\\ -\frac{a^*B^*-b^*A^*}{d}& 1 \end{pmatrix},\quad k\in \mathbb {R}\setminus \{|k|<\epsilon \},\\ \begin{pmatrix} 1& \frac{\tilde{a}\tilde{B}-\tilde{b}\tilde{A}}{\tilde{d}^*}\\ \frac{\tilde{a}^*\tilde{B}^*-\tilde{b}^*\tilde{A}^*}{\tilde{d}}& \frac{1}{\tilde{d}\tilde{d}^*} \end{pmatrix},\quad k\in \mathbb {R}\cap \{|k|<\epsilon \},\\ \begin{pmatrix} {1}& \frac{A\tilde{B}-B\tilde{A}}{\tilde{d}^* d^*}\\ 0& {1} \end{pmatrix},\quad k\in \mathbb {C}^+\cap \{|k|=\epsilon \},\\ \begin{pmatrix} {1}& 0\\ \frac{A^*\tilde{B}^*-B^*\tilde{A}^*}{\tilde{d} d}& {1} \end{pmatrix},\quad k\in \mathbb {C}^-\cap \{|k|=\epsilon \}. \end{array}\right. } \end{aligned}$$

The properties of $\check{M}^{(xt)}$ stated above give rise to a family of Riemann–Hilbert factorization problems parametrized by (*x*, *t*).

**The Riemann–Hilbert problem**  **for** $\check{M}^{(xt)}(x,t,k)$: Given *a*(*k*), *b*(*k*), *A*(*k*), *B*(*k*) for $k\in \overline{\mathbb {C}^+}$, and the set $\{\eta _j\}_1^N\subset \mathbb {C}^+$, find a piece-wise meromorphic 2×2 matrix-valued function $M^{(xt)}(x,t,k)$ that satisfies the following conditions:

1. Jump condition (5.10) across $\mathbb {R}\cup |k|=\epsilon$.
2. Behavior at ∞:
  5.19 $$\begin{aligned} \check{M}^{(xt)}(x, t,k)=I+O\left( \frac{1}{ k}\right) ,\quad k\rightarrow \infty . \end{aligned}$$
3. Residue conditions (5.16).

Similarly to Problem I, the following proposition holds:

#### Proposition 5.5

There exists a unique solution $\check{M}^{(xt)}(x,t,k)$ of the RH problem  for all *x* and *t*. Moreover, $\det \check{M}^{(xt)}\equiv 1$ and

5.20 $$\begin{aligned} \check{M}^{(xt)}( k)=\overline{\check{M}^{(xt)}(-\bar{k})},\qquad \check{M}^{(xt)}(k)=\sigma _2\overline{\check{M}^{(xt)}(\bar{k})}\sigma _2 \end{aligned}$$

## Recovering *u*(*x*, *t*) from the solution of the RH problem

In this subsection, we describe the derivation of a (local) solution to the sG equation (together with the derivation of the corresponding Lax pair equations), starting from a RH problem parametrized by *x* and *t*, motivated by the considerations in Sect. 5.

Let Γ denote an oriented contour in the complex plane, invariant under the symmetries k↦-k and $k\mapsto \bar{k}$. Suppose that ${\mathbb {C}}\setminus \Gamma = D_1\cup D_2$ so that Γ is the counterclockwise boundary of $D_1$ (the clockwise boundary of $D_2$).

Consider the following **RH problem parametrized by** *x* **and** *t*: Given the 2×2-matrix-valued function $J_0(k)\in L^2(\Gamma )\cap L^{\infty }(\Gamma )$ for $k\in \Gamma$ such that

6.1a $$\begin{aligned}&\det J_0(k)\equiv 1,\end{aligned}$$

6.1b $$\begin{aligned}&J_0(k)=I+O\bigg (\frac{1}{k}\bigg ),\quad k\rightarrow \infty ,\end{aligned}$$

6.1c $$\begin{aligned}&J_0(k)=I+O(k),\quad k\rightarrow 0, \end{aligned}$$

(whenever the corresponding points lie on Γ) and the sets $\{k_j,c_j\}_1^N$ with $k_j\in D_1$, and $\{\eta _j,d_j\}_1^M$ with $\eta _j\in D_2$, find a piece-wise meromorphic (w.r.t. Γ) 2×2-matrix-valued function $\hat{M}(x,t,k)$ satisfying:

- *Jump* condition
  6.2 $$\begin{aligned} M_+(x,t,k)=M_-(x,t,\lambda ) J(x,t,k),\qquad k\in \Sigma , \end{aligned}$$
  where $$J(x,t,k)=\textrm{e}^{-ik(x-\frac{t}{4k^2})\sigma _3}J_0(k)\textrm{e}^{ik(x-\frac{t}{4k^2})\sigma _3}$$.
- *Normalization* condition:
  6.3 $$\begin{aligned} \hat{M}(x,t,k)=I+\textrm{O}\bigg (\frac{1}{k}\bigg ), \quad k\rightarrow \infty . \end{aligned}$$
- *Residue* conditions:
  6.4 $$\begin{aligned} {{\,\textrm{Res}\,}}_{ k_j} M^{(1)}(x,t,k)&= c_j M^{(2)}(x,t, k_j) \textrm{e}^{-2\textrm{i}k_j (x-\frac{t}{4k_j^2})}, ~ k_j\in D_1, \end{aligned}$$
  6.5 $$\begin{aligned} {{\,\textrm{Res}\,}}_{\eta _j} M^{(2)}(x,t,k)&=d_j M^{(1)}(x,t,\eta _j)\textrm{e}^{2\textrm{i}\eta _j (x-\frac{t}{4\eta _j^2})} , ~ \eta _j\in D_2. \end{aligned}$$

The following Proposition holds:

### Proposition 6.1

1. If *M* is a solution of RH problem (6.2)–(6.5), then detM=1.
2. If a solution of the RH problem (6.2)–(6.5) exists, it is unique.

Now, assume that the RH problem (6.2)–(6.5) has a solution *M*(*x*, *t*, *k*) that satisfies the *symmetries*

6.6 $$\begin{aligned} M(k)=\overline{ M(-\bar{k})} \end{aligned}$$

and

6.7 $$\begin{aligned} M(k)=\begin{pmatrix} 0& -\textrm{i}\\ \textrm{i}& 0 \end{pmatrix}\overline{ M(\bar{k})}\begin{pmatrix} 0& -\textrm{i}\\ \textrm{i}& 0 \end{pmatrix} \end{aligned}$$

and is differentiable w.r.t. *x* and *t*.

Now, our goal is to show that, by evaluating the solution *M*(*x*, *t*, *k*) of the RH problem (6.2)–(6.5) at appropriate points of the extended complex plane $\overline{\mathbb {C}}$, one can obtain a solution of the sG equation.

We proceed as follows:

- Starting from *M*(*x*, *t*, *k*), define 2×2-matrix-valued functions
  6.8 $$\begin{aligned} \Psi (x,t,k):= M(x,t,k)\textrm{e}^{-ik(x-\frac{t}{4k^2})\sigma _3} \end{aligned}$$
  and show that Ψ(x,t,k) satisfies the system of differential equations:
  6.9 $$\begin{aligned} \begin{aligned} \Psi _x&=\hat{U}\Psi , \\ \Psi _t&=\hat{V}\Psi , \end{aligned} \end{aligned}$$
  where $\hat{U}$ and $\hat{V}$ have the same (rational) dependence on *k* as in (2.2), with coefficients given in terms of *M*(*x*, *t*, *k*) evaluated at appropriate values of *k*.
- Show that the compatibility condition for (6.9), i.e., the equality $\hat{U}_t - \hat{V}_x + [\hat{U},\hat{V}]=0$, reduces to (1.1).

### Proposition 6.2

Let *M*(*x*, *t*, *k*) be the solution of the RH problem (6.2)–(6.5) that satisfies symmetries (6.6) and (6.7). Then Ψ(x,t,k) defined by (6.8) satisfies the differential equation

6.10 $$\begin{aligned} \hat{\Psi }_x=\hat{U}\hat{\Psi } \end{aligned}$$

with

6.11 $$\begin{aligned} \hat{U}(x,t,k)=-\textrm{i}k \sigma _3 + \frac{\xi (x,t)}{2}{}\begin{pmatrix} 0&  \quad 1\\ -1&  \quad 0 \end{pmatrix}, \end{aligned}$$

where $\xi (x,t)\in \mathbb {R}$ can be obtained from the large *k* expansion of *M*(*x*, *t*, *k*):

6.12 $$\begin{aligned} M(x,t,k)=I+\frac{1}{4\textrm{i}k}\begin{pmatrix} -\eta (x,t) &  \xi (x,t)\\ \xi (x,t)&  \eta (x,t) \end{pmatrix}+O\bigg (\frac{1}{k^2}\bigg ),\qquad k\rightarrow \infty . \end{aligned}$$

### Proof

First, notice that Ψ(x,t,k) satisfies the jump condition

$$ \Psi ^+(x,t,k)=\Psi ^-(x,t,k)J_0(k) $$

with the jump matrix $J_0(k)$ independent of *x*. Hence, $\Psi _x(x,t,k)$ satisfies the same jump condition.

As for the residue conditions, we notice that the symmetry assumptions (6.6) and (6.7) imply that if $$ k_j$$ is pole of $$\hat{M}^{(1)}$$ then $$-\bar{k}_j$$ is also pole of $$\hat{M}^{(1)}$$ with $$c_{ k_j}=-\overline{c_{-\bar{k}_j}}$$. Analogously, if $$\eta _j$$ is pole of $$\hat{M}^{(2)}$$ then $$-\bar{\eta }_j$$ is also pole of $$\hat{M}^{(2)}$$ with $$d_{\eta _j}=-\overline{d_{-\bar{\eta }_j}}$$. Moreover,

6.13 $$\begin{aligned} \eta _j=- k_j, \quad d_j=c_j. \end{aligned}$$

Since $\{c_j\}_1^N$ are independent of *x*, $\Psi _x(x,t,k)$ satisfies the same residue conditions as Ψ(x,t,k) does:

$$\begin{aligned} {{\,\textrm{Res}\,}}_{ k_j}\Psi ^{(1)}&= c_j \Psi ^{(2)}( k_j) , ~ k_j\in D_1, \\ {{\,\textrm{Res}\,}}_{- k_j} \Psi ^{(2)}&= c_j\Psi ^{(1)}(- k_j) , ~ - k_j\in D_2. \end{aligned}$$

Therefore, $$ \Psi _x \Psi ^{-1}= M_x M^{-1}-\textrm{i}k M \sigma _3 M ^{-1}$$ has neither jump nor singularities at $$ k_j$$, and thus, it is a meromorphic function, with a possible singularity at k=∞.

Let us analyze the behavior of $\Psi _x \Psi ^{-1}$ as $k\rightarrow \infty$. First of all, notice that the symmetry (6.6) implies

6.14 $$\begin{aligned} M = I+\frac{\textrm{i}}{k}\begin{pmatrix} m_{11}^\infty &  \quad m_{12}^\infty \\ m_{21}^\infty &  \quad m_{22}^\infty \end{pmatrix}+O\bigg (\frac{1}{k^2}\bigg ), \quad k\in \mathbb {C}_+ \end{aligned}$$

with $\hat{m}_{+ij}^\infty \in \mathbb {R}$. Moreover, the symmetry (6.7) implies

6.15 $$\begin{aligned} M = I-\frac{\textrm{i}}{k}\begin{pmatrix} m_{22}^\infty &  - m_{21}^\infty \\ - m_{12}^\infty &  m_{11}^\infty \end{pmatrix}+O\bigg (\frac{1}{k^2}\bigg ), \quad k\in \mathbb {C}_-. \end{aligned}$$

Therefore, $M_x=O(\frac{1}{k})$ and thus $M_x M^{-1}=O(\frac{1}{k})$, which leads to the following expansion for $\Psi _x\Psi ^{-1}$:

6.16 $$\begin{aligned} \Psi _x \Psi ^{-1}=-\textrm{i}k \sigma _3 + \begin{pmatrix} m_{11}^\infty + m_{22}^\infty & -2 m_{12}^\infty \\ 2 m_{21}^\infty & - m_{11}^\infty - m_{22}^\infty \end{pmatrix}+O\bigg (\frac{1}{k}\bigg ), \quad k\rightarrow \infty , \quad k\in \mathbb {C}_+, \end{aligned}$$

6.17 $$\begin{aligned} \Psi _x \Psi ^{-1}=-\textrm{i}k \sigma _3 - \begin{pmatrix} m_{11}^\infty + m_{22}^\infty & 2 m_{21}^\infty \\ -2 m_{12}^\infty & - m_{11}^\infty - m_{22}^\infty \end{pmatrix}+O\bigg (\frac{1}{k}\bigg ), \quad k\rightarrow \infty , \quad k\in \mathbb {C}_-. \end{aligned}$$

Since $\Psi _x \Psi ^{-1}$ is meromorphic in $\mathbb {C}$ these expansions should coincide, and thus, $m_{11}^\infty =- m_{22}^\infty$ and $m_{12}^\infty = m_{21}^\infty$.

Denoting $\eta =4 m_{11}$ and $\xi =-4m_{12}$, we get (6.12). Therefore, Liouville’s theorem yields (6.10)–(6.11). □

### Proposition 6.3

Ψ(x,t,k) defined in (6.8) satisfies the differential equation

6.18 $$\begin{aligned} \Psi _t=\hat{V}\Psi \end{aligned}$$

with

6.19 $$\begin{aligned} \hat{V}=-\frac{1}{4\textrm{i}k} \begin{pmatrix} \alpha ^2-\beta ^2& -2\alpha \beta \\ -2 \alpha \beta & -\alpha ^2+\beta ^2 \end{pmatrix}, \end{aligned}$$

where $\alpha (x,t)\in \mathbb {R}$ and $\beta (x,t)\in \mathbb {R}$ can be obtained from the from the expansion of *M*(*x*, *t*, *k*) as k→0:

6.20 $$\begin{aligned} M(x,t,k)=\begin{pmatrix} \alpha (x,t) &  \beta (x,t)\\ -\beta (x,t)&  \alpha (x,t) \end{pmatrix}+ O(k),\quad k\rightarrow 0 \end{aligned}$$

with

6.21 $$\begin{aligned} \alpha ^2(x,t)+\beta ^2(x,t)=1. \end{aligned}$$

### Proof

Similarly to Proposition 6.2, we notice that $$\Psi _t \Psi ^{-1}=M_t M^{-1}+\frac{\textrm{i}}{4k} M\sigma _3M^{-1}$$ has neither jump nor poles at $$ k_j$$, and thus, it is a meromorphic function, with possible singularities at k=∞ and k=0:

Evaluating $\Psi _t\Psi ^{-1}$ near these points, we have the following.

(i) As $k\rightarrow \infty$, we have $M M_t^{-1}=O(\frac{1}{k})$, $p_t(k)=O(\frac{1}{k})$ and thus
  6.22 $$\begin{aligned} \hat{\Psi }_t\hat{\Psi }^{-1}(k)=\textrm{O}(k^{-1}),\qquad k\rightarrow \infty . \end{aligned}$$
(ii) As k→0, first notice that (6.1c) together with the symmetries (6.6) and (6.7) implies (6.20), and the Proposition 6.1 yields (6.21). Therefore,
  6.23 $$\begin{aligned} \hat{\Psi }_t \hat{\Psi }^{-1}=-\frac{1}{4\textrm{i}k} \begin{pmatrix} \hat{\alpha }^2- \hat{\beta }^2& -2 \hat{\alpha } \hat{\beta }\\ -2 \hat{\alpha } \hat{\beta }& -\hat{\alpha }^2+\hat{\beta }^2 \end{pmatrix}+O(1), \quad k\rightarrow 0. \end{aligned}$$
  Combining (6.22) and (6.23) with the Liouville’s theorem yields (6.18)–(6.20).

□

We now show that the compatibility condition

6.24 $$\begin{aligned} \hat{U}_t - \hat{V}_x + [\hat{U},\hat{V}]=0 \end{aligned}$$

yields the sG equation. For this purpose, substituting into (6.24) the expressions for $\hat{U}$ and $\hat{V}$ from (6.11) and (6.19), respectively, and equating to 0 the coefficients at various expressions involving *k*, we get algebraic and differential equations among α, β, and ξ:

### Proposition 6.4

Let α(x,t), β(x,t), and ξ(x,t) be the functions determined in terms of *M*(*x*, *t*, *k*) as in Propositions 6.2 and 6.3. Then, they satisfy the following equations:

6.25a $$\begin{aligned}&\xi _t- 2\alpha \beta =0,\end{aligned}$$

6.25b $$\begin{aligned}&\alpha \alpha _x-\beta \beta _x+ \xi \beta \alpha =0,\end{aligned}$$

6.25c $$\begin{aligned}&2\alpha _x \beta +2\alpha \beta _x- \xi ( \alpha ^2- \beta ^2)=0. \end{aligned}$$

### Proposition 6.5

Introducing *u*(*x*, *t*) up to 2πk such that

6.26 $$\begin{aligned} \sin \frac{u}{2} = \beta , \qquad \cos \frac{u}{2} =\alpha \end{aligned}$$

or

6.27 $$\begin{aligned} \sin \frac{u}{2} = -\beta , \qquad \cos \frac{u}{2} =-\alpha \end{aligned}$$

equations (6.25a)– (6.25c) reduce to (1.1).

### Proof

First notice that (6.26) implies

$$ \sin u=2\alpha \beta ,\qquad \cos u=\alpha ^2-\beta ^2. $$

Moreover, since $\alpha ^2+\beta ^2=1$, α and β cannot be simultaneously 0.

Combining these facts with (6.25c) (in case $\alpha ^2-\beta ^2\ne 0$) or (6.25b) (in case $2\alpha \beta \ne 0$), we conclude that

$$ \xi =u_x. $$

Consequently, (6.25a) implies (1.1). □

## Differentiability of solution of master RH problems

### Basic facts about $L^2$-RH problems

For our purposes, we will need several results concerning the equivalence between $L^2$ Riemann–Hilbert problems (whose jump matrices may be discontinuous at intersection points of the contour) and singular integral equations; see, for example, [22, 23]. We collect the necessary definitions and results below.

Throughout this section, Γ denotes an oriented contour consisting of a finite union of piece-wise $C^1$ arcs. The arcs may intersect only at their endpoints, and all intersections are assumed to be transversal. In particular, this class of contours includes configurations such as the union of the real line with finitely many circles.

The orientation of Γ is chosen so that

$$ \Gamma =\partial D_+=-\partial D_-, $$

where $$\mathbb {C}\setminus \Gamma =D_+\cup D_-$$.

For a bounded component *D* of $\mathbb {C}\setminus \Gamma$, denote by $E^2(D)$ the class of analytic functions *f* in *D* for which there exists a sequence of rectifiable Jordan curves $\{C_n\}\subset D$ such that for every compact set $D_c\subset D$ there exists *N* so that $C_n$ surrounds $D_c$ for all n>N and

$$ \sup _{n\ge 1}\int _{C_n}|f(z)|^2|dz|<\infty . $$

If *D* is unbounded, define $f\in E^2(D)$ if $f\circ \phi ^{-1}\in E^2(\phi (D))$, where

$$ \phi (z)=\frac{1}{z-z_0} $$

for some $z_0\in \mathbb {C}\setminus \overline{D}$. We also write

$$ \dot{E}^2(D)=\{f\in E^2(D):zf(z)\in E^2(D)\}, $$

that is, f(z)→0 as $z\rightarrow \infty$.

Denote by $\mathcal {B}(L^2(\Gamma ))$ the space of bounded linear operators on $L^2(\Gamma )$ and by $\mathcal {F}(L^2(\Gamma ))$ the set of Fredholm operators on $L^2(\Gamma )$. The index map

$$ \textrm{Ind}:\mathcal {F}(L^2(\Gamma ))\rightarrow \mathbb {Z} $$

is constant on the connected components of $\mathcal {F}(L^2(\Gamma ))$.

For $h\in L^2(\Gamma )$, the Cauchy transform is defined by

7.1 $$\begin{aligned} (Ch)(\lambda )=\frac{1}{2\pi i}\int _\Gamma \frac{h(z)}{z-\lambda }\,dz . \end{aligned}$$

The non-tangential limits of *Ch* as λ approaches Γ from the left and right exist and will be denoted by $C_+h$ and $C_-h$, respectively. The operators $C_\pm$ are bounded on $L^2(\Gamma )$ and satisfy

$$ C_+-C_-=1\!\!1, \quad (C_+)^2=C_+,\quad (C_-)^2=-C_-, \quad C_+C_-=C_-C_+=0. $$

For functions $w^\pm \in L^2(\Gamma )\cap L^\infty (\Gamma )$ define the operator $C_w:L^2(\Gamma )+L^\infty (\Gamma )\rightarrow L^2(\Gamma )$ by

7.2 $$\begin{aligned} C_w(f)=C_+(fw^-)+C_-(fw^+). \end{aligned}$$

Moreover,

7.3a $$\begin{aligned} ||C_w||_{\mathcal {B}(L^2(\Gamma ))}&\le C \max \{||w^+||_{L^\infty }(\Gamma ),||w^-||_{L^\infty }(\Gamma )\},\end{aligned}$$

7.3b $$\begin{aligned} ||C_w h||_{L^2(\Gamma )}&\le C ||h||_{L^\infty (\Gamma )} \max \{||w^+||_{L^2(\Gamma )},||w^-||_{L^2(\Gamma )}\} \end{aligned}$$

with $$C=2\max \{||C_+||_{\mathcal {B}(L^2(\Gamma ))},||C_-||_{\mathcal {B}(L^2(\Gamma ))}\}$$. where

$$ C=2\max \{\Vert C_+\Vert _{\mathcal {B}(L^2(\Gamma ))},\Vert C_-\Vert _{\mathcal {B}(L^2(\Gamma ))}\}. $$

The following lemma ( [22]) establishes the equivalence between the $L^2$ Riemann–Hilbert problem determined by (Γ,v) and the singular integral equation for $\mu \in I+L^2(\Gamma )$

7.4 $$\begin{aligned} \mu -I=C_w(\mu ). \end{aligned}$$

#### Lemma 7.1

Given $v^\pm :\Gamma \rightarrow GL(n,\mathbb {C})$. Let $v=(v^-)^{-1}v^+$, $w^+=v^+-I$, $w^-=I-v^-$. Suppose $v^\pm$, $(v^\pm )^{-1}\in I + L^2(\Gamma )\cap L^\infty (\Gamma )$. If $m\in I + \dot{E}^2(D)$ satisfies $L^2-$RH problem determined by $(\Gamma ,v(\lambda ))$, then $\mu = m_+(v^+)^{-1}= m_-(v^-)^{-1}\in I+L^2(\Gamma )$ satisfies (7.4). Conversely, if $\mu \in I+L^2(\Gamma )$ satisfies (7.4), then $m=I+C(\mu (w^+ + w^-))\in I + \dot{E}^2(D)$ satisfies $L^2-$RH problem determined by $(\Gamma ,v(\lambda ))$.

The following lemma ( [22]) shows that operator $1\!\!1-C_w$ is Fredholm.

#### Lemma 7.2

Given $v^\pm :\Gamma \rightarrow GL(n,\mathbb {C})$. Let $v=(v^-)^{-1}v^+$, $w^+=v^+-I$, $w^-=I-v^-$. Suppose $v^\pm$, $(v^\pm )^{-1}\in I + L^2(\Gamma )\cap L^\infty (\Gamma )$, and $v^\pm$ is piece-wise continuous on Γ. Then

1. The operator $1\!\!1-C_w:L^2(\Gamma )\rightarrow L^2(\Gamma )$ is Fredholm.
2. If $w^\pm$ are nilpotent, then $1\!\!1-C_w$ has Fredholm index 0; in this case TFAE:
  - The map $1\!\!1-C_w:L^2(\Gamma )\rightarrow L^2(\Gamma )$ is bijective.
  - The $L^2-$RH problem determined by $(\Gamma ,v(\lambda ))$ has a unique solution.
  - The homogeneous $L^2-$RH problem determined by $(\Gamma ,v(\lambda ))$ has only the zero solution.
  - The map $1\!\!1-C_w:L^2(\Gamma )\rightarrow L^2(\Gamma )$ is injective.

### Problem I

We assume that *a*(*k*) has no zeros in $\overline{\mathbb {C}^+}$. Zeros in the open upper half-plane can be eliminated by introducing small circular contours and converting the residue conditions into jump conditions; zeros on $\mathbb {R}$ could be eliminated by introducing small half-circular contours.

First, notice that the jump matrix (5.2b) can be written in the form suitable for Lemma 7.1 and 7.2. Indeed, $[J^{(xt)}]^{(-1)}(x,t,k)=(v^-)^{-1}v^+$ with

7.5 $$\begin{aligned} v^-(x,t,k)={\left\{ \begin{array}{ll} \begin{pmatrix} 1& -r^*(k)\textrm{e}^{-2\textrm{i}kp(x,t,k)}\\ 0& 1 \end{pmatrix},\quad k\in \mathbb {R}\cap \{|k| >\epsilon \},\\ \begin{pmatrix} 1& 0\\ \tilde{r}(k)\textrm{e}^{2\textrm{i}kp(x,t,k)}& 1 \end{pmatrix},\quad k\in \mathbb {R}\cap \{|k| <\epsilon \},\\ I,\quad k\in \mathbb {C}^+\cap \{|k| =\epsilon \},\\ \begin{pmatrix} 1& -\frac{\kappa _2^0\textrm{e}^{-2\textrm{i}kp(x,t,k)}}{a^*(k)\tilde{a}^*(k)}\\ 0& 1 \end{pmatrix},\quad k\in \mathbb {C}^-\cap \{|k| =\epsilon \} \end{array}\right. } \end{aligned}$$

and

7.6 $$\begin{aligned} v^+(x,t,k)={\left\{ \begin{array}{ll} \begin{pmatrix} 1& 0\\ r(k)\textrm{e}^{2\textrm{i}kp(x,t,k)}& 1 \end{pmatrix},\quad k\in \mathbb {R}\cap \{|k| >\epsilon \},\\ \begin{pmatrix} 1& -\tilde{r}^*(k)\textrm{e}^{-2\textrm{i}kp(x,t,k)}\\ 0& 1 \end{pmatrix},\quad k\in \mathbb {R}\cap \{|k| <\epsilon \},\\ \begin{pmatrix} 1& 0\\ \frac{\kappa _2^0\textrm{e}^{2\textrm{i}kp(x,t,k)}}{a(k)\tilde{a}(k)}& 1 \end{pmatrix},\quad k\in \mathbb {C}^+\cap \{|k| =\epsilon \},\\ I,\quad k\in \mathbb {C}^-\cap \{|k| =\epsilon \}. \end{array}\right. } \end{aligned}$$

Moreover, from the considerations in Sect. 2.1.1, it follows that *r*(*k*), $\tilde{r}(k)$ and $\frac{1}{a(k)\tilde{a}(k)}$ satisfy the following properties:

7.7 $$\begin{aligned}&r(k)=O\left( \frac{1}{k}\right) ,\quad k\in \mathbb {R},~ k\rightarrow \infty ;\quad r(k)\in C(\mathbb {R}), \end{aligned}$$

7.8 $$\begin{aligned}&\tilde{r}(k)=O(k) ,\quad k\in \mathbb {R},~ k\rightarrow 0;\quad r(k)\in C(\mathbb {R}),\end{aligned}$$

7.9 $$\begin{aligned}&\frac{1}{a(k)\tilde{a}(k)}\in C(\{|k|=\epsilon \}),\quad k\in \mathbb {C}^+. \end{aligned}$$

Therefore, using $\det v^\pm =1$, we conclude that

$$ v^\pm ,v_\pm ^{-1}\in I + L^2(\Sigma )\cap L^\infty (\Sigma ). $$

Hence, Lemma 7.1 applies and yields the equivalence between the RH problem ${\textbf {RH}}^{(xt)}$ and the associated singular integral equation.

Accordingly, $w^\pm$ are given by

7.10 $$\begin{aligned} w^-(x,t,k)={\left\{ \begin{array}{ll} \begin{pmatrix} 0& -r^*(k)\textrm{e}^{-2\textrm{i}kp(x,t,k)}\\ 0& 0 \end{pmatrix},\quad k\in \mathbb {R}\cap \{|k| >\epsilon \},\\ \begin{pmatrix} 0& 0\\ \tilde{r}(k)\textrm{e}^{2\textrm{i}kp(x,t,k)}& 0 \end{pmatrix},\quad k\in \mathbb {R}\cap \{|k| <\epsilon \},\\ 0,\quad k\in \mathbb {C}^+\cap \{|k| =\epsilon \},\\ \begin{pmatrix} 0& -\frac{\kappa _2^0\textrm{e}^{-2\textrm{i}kp(x,t,k)}}{a^*(k)\tilde{a}^*(k)}\\ 0& 0 \end{pmatrix},\quad k\in \mathbb {C}^-\cap \{|k| =\epsilon \} \end{array}\right. } \end{aligned}$$

and

7.11 $$\begin{aligned} w^+(x,t,k)={\left\{ \begin{array}{ll} \begin{pmatrix} 0& 0\\ r(k)\textrm{e}^{2\textrm{i}kp(x,t,k)}& 0 \end{pmatrix},\quad k\in \mathbb {R}\cap \{|k| >\epsilon \},\\ \begin{pmatrix} 0& -\tilde{r}^*(k)\textrm{e}^{2\textrm{i}kp(x,t,k)}\\ 0& 0 \end{pmatrix},\quad k\in \mathbb {R}\cap \{|k| <\epsilon \},\\ \begin{pmatrix} 0& 0\\ \frac{\kappa _2^0\textrm{e}^{2\textrm{i}kp(x,t,k)}}{a(k)\tilde{a}(k)}& 0 \end{pmatrix},\quad k\in \mathbb {C}^+\cap \{|k| =\epsilon \},\\ 0,\quad k\in \mathbb {C}^-\cap \{|k| =\epsilon \}, \end{array}\right. } \end{aligned}$$

and since $w^\pm$ are nilpotent, we can apply Lemma 7.2, which together with Zhou’s vanishing lemma ensures the solvability of the RH problem ${\textbf {RH}}^{(xt)}$. In particular,

7.12 $$\begin{aligned} \mu ^{(xt)}(x,t,k)=I+(1\!\!1-C_w)^{-1}(x,t)C_w(x,t)I \end{aligned}$$

and the solution $M^{(xt)}(x,t,k)$ of the Riemann–Hilbert problem ${\textbf {RH}}^{(xt)}$ can be written in terms of $\mu ^{(xt)}(x,t,k)$ as

7.13 $$\begin{aligned} {M^{(xt)}}(x,t,k)=I+\frac{1}{2\pi \textrm{i}}\int _\Sigma \frac{\mu ^{(xt)}(x,t,z)(w^+ + w^-)(x,t,z)}{z-k}dz. \end{aligned}$$

Notice that

7.14 $$\begin{aligned} C_w(x,t)= C_{w(x,t)} \end{aligned}$$

and $C_w$ is linear in *w*.

To study the differentiability with respect to *x* and *t*, it is convenient to modify the RH problem so that its jump matrix approaches the identity sufficiently fast as $k\rightarrow \infty$, at least as $O\left( \frac{1}{k^2}\right)$. The jump matrix of the original problem decays only as $r(k)=O\left( \frac{1}{k}\right)$, and is of order *O*(*k*) as k→0. To improve this behavior, we introduce two functions (c.f. [24])

$$ h(k):=\frac{r_1}{4\textrm{i}k},\quad \tilde{h}(k)=\textrm{i}\tilde{r}_1 k $$

where $r_1$ and $\tilde{r}_1$ are determined by the expansions $r(k)=\frac{r_1}{4\textrm{i}k}+O\left( \frac{1}{k^2}\right)$ and $\tilde{r}(k)=\textrm{i}\tilde{r}_1 k+O(k^2)$. Notice that

7.15 $$\begin{aligned} \overline{h(-\bar{k})}=h(k),\quad h(k)\in C(\{|k|=\epsilon \}), \quad h(k)=O\left( \frac{1}{k}\right) ,\quad k\rightarrow \infty . \end{aligned}$$

7.16 $$\begin{aligned} \overline{\tilde{h}(-\bar{k})}=\tilde{h}(k),\quad \tilde{h}(k)\in C(\{|k|=\epsilon \}), \quad \tilde{h}(k)=O\left( k\right) ,\quad k\rightarrow 0. \end{aligned}$$

Moreover, by the definition of *h*(*k*), we have

7.17 $$\begin{aligned} h(k)-r(k)=O\left( \frac{1}{k^2}\right) , \quad k\rightarrow \infty ;\quad \tilde{h}(k)-\tilde{r}(k)=O\left( k^2\right) , \quad k\rightarrow 0. \end{aligned}$$

We then introduce a matrix-valued function $\hat{M}(x,t,k)$ related to *M*(*x*, *t*, *k*) by

7.18 $$\begin{aligned} \hat{M}(x,t,k)={\left\{ \begin{array}{ll} M(x,t,k)\textrm{e}^{-\textrm{i}k p(x,t,k)\sigma _3}\begin{pmatrix} 1& 0\\ -h(k)& 1 \end{pmatrix}\textrm{e}^{\textrm{i}k p(x,t,k)\sigma _3},\quad \{|k|>\epsilon \}\cap \mathbb {C}_+,\\ M(x,t,k)\textrm{e}^{-\textrm{i}k p(x,t,k)\sigma _3}\begin{pmatrix} 1& h^*(k)\\ 0& 1 \end{pmatrix}\textrm{e}^{\textrm{i}k p(x,t,k)\sigma _3},\quad \{|k|>\epsilon \}\cap \mathbb {C}_-,\\ M(x,t,k)\textrm{e}^{-\textrm{i}k p(x,t,k)\sigma _3}\begin{pmatrix} 1& 0\\ -\tilde{h}(k)& 1 \end{pmatrix}\textrm{e}^{\textrm{i}k p(x,t,k)\sigma _3},\quad \{|k|<\epsilon \}\cap \mathbb {C}_+,\\ M(x,t,k)\textrm{e}^{-\textrm{i}k p(x,t,k)\sigma _3}\begin{pmatrix} 1& \tilde{h}^*(k)\\ 0& 1 \end{pmatrix}\textrm{e}^{\textrm{i}k p(x,t,k)\sigma _3},\quad \{|k|<\epsilon \}\cap \mathbb {C}_- \end{array}\right. } \end{aligned}$$

and conclude that it satisfies the following RH problem:

1. Jump condition across $\mathbb {R}\cup \{|k|=\epsilon \}$
  7.19a $$\begin{aligned} \hat{M}_-^{(xt)}(x,t,k)=\hat{M}_+^{(xt)}(x,t,k)\hat{J}^{(xt)}(x,t,k),\quad k\in \mathbb {R}\cup \{|k|=\epsilon \} \end{aligned}$$
  where
  7.19b $$\begin{aligned} \hat{J}^{(xt)}(x,t,k)=\textrm{e}^{-\textrm{i}kp(x,t,k)\sigma _3} \hat{J}^{(xt)}_0(k)\textrm{e}^{\textrm{i}kp(x,t,k)\sigma _3} \end{aligned}$$
  with $$p(x,t,k)=x-\frac{t}{4 k^2}$$ and
  7.19c $$\begin{aligned} \hat{J}^{(xt)}_0(k)={\left\{ \begin{array}{ll} \begin{pmatrix} 1& 0\\ h(k)-r(k)& 1 \end{pmatrix} \begin{pmatrix} 1& h^*(k)-r^*(k)\\ 0& 1 \end{pmatrix},\quad k\in \mathbb {R}\cap \{|k| >\epsilon \},\\ \begin{pmatrix} 1& \tilde{r}^*(k)-\tilde{h}^*(k)\\ 0& 1 \end{pmatrix}\begin{pmatrix} 1& 0\\ \tilde{r}(k)-\tilde{h}(k)& 1 \end{pmatrix},\quad k\in \mathbb {R}\cap \{|k| <\epsilon \},\\ \begin{pmatrix} 1& 0\\ h(k)-\tilde{h}(k)-\frac{\kappa _2^0}{a(k)\tilde{a}(k)}& 1 \end{pmatrix},\quad k\in \mathbb {C}^+\cap \{|k| =\epsilon \},\\ \begin{pmatrix} 1& h^*(k)-\tilde{h}^*(k)-\frac{\kappa _2^0}{a^*(k)\tilde{a}^*(k)}\\ 0& 1 \end{pmatrix},\quad k\in \mathbb {C}^-\cap \{|k| =\epsilon \} \end{array}\right. } \end{aligned}$$
  and $$r(k)=\frac{b^*(k)}{a(k)}$$, $$\tilde{r}(k)=\frac{\tilde{b}^*(k)}{\tilde{a}(k)}$$.
2. Behavior at ∞:
  7.20 $$\begin{aligned} \hat{M}^{(xt)}(x, t,k)=I+O\left( \frac{1}{ k}\right) ,\quad k\rightarrow \infty . \end{aligned}$$

All the considerations above apply to this RH problem as well. In particular,

7.21 $$\begin{aligned} \hat{\mu }^{(xt)}(x,t,k)=I+(1\!\!1-C_{\hat{w}})^{-1}(x,t)C_{\hat{w}}(x,t)I \end{aligned}$$

and

7.22 $$\begin{aligned} {\hat{M}^{(xt)}}(x,t,k)=I+\frac{1}{2\pi \textrm{i}}\int _\Sigma \frac{\hat{\mu }^{(xt)}(x,t,z)(\hat{w}^+ + \hat{w}^-)(x,t,z)}{z-k}dz. \end{aligned}$$

Here $\hat{w}_\pm$ are defined as in (7.11) and 7.10, with *r*(*k*), $\tilde{r}(k)$, and $\frac{\kappa _2^0}{a(k)\tilde{a}(k)}$ replaced by r(k)-h(k), $\tilde{r}(k)-\tilde{h}(k)$, and $\frac{\kappa _2^0}{a(k)\tilde{a}(k)}-h(k)+\tilde{h}(k)$, respectively.

The differentiability of $M^{(xt)}(x,t,k)$ then follows from the differentiability of $\hat{M}^{(xt)}(x,t,k)$ established in

#### Proposition 7.3

The function $\mu ^{(xt)}(x,t,k)$ is $C^1$ with respect to *x* and *t*.

#### Proof

Notice that $\hat{\mu }^{(xt)}(x,t,k)$ is a composition of the following maps:

$$\begin{aligned}&(x,t)\mapsto C_{\hat{w}(x,t)}I: (\mathbb {R}^+,[0,T))\rightarrow L^2(\Sigma );\\&(x,t)\mapsto (1\!\!1-C_{\hat{w}(x,t)})^{-1}:(\mathbb {R}^+,[0,T))\rightarrow \mathcal {B}(L^2(\Sigma )). \end{aligned}$$

In turn, $(x,t)\mapsto C_{\hat{w}(x,t)}I: (\mathbb {R},[0,\infty )\rightarrow L^2(\Sigma )$ is a composition of

$$\begin{aligned}&(x,t)\mapsto \hat{w}^\pm (x,t):(\mathbb {R}^+,[0,T))\rightarrow L^2(\Sigma );\\&Q:(\hat{w}^+,\hat{w}^-)\mapsto C_{\hat{w} }I:L^2(\Sigma )\times L^2(\Sigma ) \rightarrow L^2(\Sigma ) \end{aligned}$$

and $(x,t)\mapsto (1\!\!1-C_{\hat{w}(x,t)})^{-1}:(\mathbb {R}^+,[0,T))\rightarrow \mathcal {B}(L^2(\Sigma ))$ is a composition of

$$\begin{aligned}&(x,t)\mapsto \hat{w}^\pm (x,t):(\mathbb {R}^+,[0,T))\rightarrow L^\infty (\Sigma );\\&F:(\hat{w}^+,\hat{w}^-)\mapsto (1\!\!1-C_{\hat{w}})^{-1}:L^\infty (\Sigma )\times L^\infty (\Sigma )\rightarrow \mathcal {B}(L^2(\Sigma )). \end{aligned}$$

Differentiating $\textrm{e}^{\textrm{i}kp(x,t,k)}$ we get

$$ \frac{d}{dx} (\textrm{e}^{2 \textrm{i}kp(x,t,k)})=2\textrm{i}k\textrm{e}^{2 \textrm{i}kp(x,t,k)}, \quad \frac{d}{dt} (\textrm{e}^{2 \textrm{i}kp(x,t,k)})=\frac{1}{2\textrm{i}k}\textrm{e}^{2 \textrm{i}kp(x,t,k)}. $$

Combining (7.7)–(7.9) with (7.15)–(7.17), we conclude that map $(x,t)\mapsto \hat{w}^\pm (x,t):(\mathbb {R}^+,[0,T))\rightarrow L^\infty (\Sigma )$ is differentiable w.r.t. both *x* and *t*. On the other hand, these properties together with the dominated convergence theorem yield the differentiability of $(x,t)\mapsto \hat{w}^\pm (x,t):(\mathbb {R}^+,[0,T))\rightarrow L^2(\Sigma )$.

The maps *F* and *Q* are linear, and estimates (7.3a) and (7.3b) imply their differentiability. Moreover, $dF:(u^+,u^-)\mapsto -C_u:L^\infty (\Sigma )\times L^\infty (\Sigma )\rightarrow \mathcal {B}(L^2(\Sigma ))$ and $dQ:(u^+,u^-)\mapsto C_u I:L^2(\Sigma )\times L^2(\Sigma )\rightarrow L^2(\Sigma )$. Hence, by the chain rule (see [25]), the map $(x,t)\mapsto (1\!\!1-C_{\hat{w}(x,t)})^{-1}:(\mathbb {R}^+,[0,T))\rightarrow \mathcal {B}(L^2(\Sigma ))$ is differentiable together with its inverse. The chain rule again implies differentiability of the map $(x,t)\mapsto C_{\hat{w}(x,t)}I: (\mathbb {R}^+,[0,T))\rightarrow L^2(\Sigma )$. Consequently, $\hat{\mu }^{(xt)}(x,t,k)$ is differentiable as the composition of differentiable maps. □

Notice that properties (7.15) and (7.16) imply that the first term in the expansion of $\hat{M}(x,t,k)$ as k→0, as well as the second term in the expansion as $k \rightarrow \infty$, coincide with those of *M*(*x*, *t*, *k*). Thus, Proposition 7.3 together with the dominated convergence theorem implies the following corollary:

#### Corollary 7.4

The functions α(x,t), β(x,t) and ξ(x,t) are $C^1$ with respect to *x* and *t*.

### Problem II

The idea of the proof that $\check{M}^{(xt)}(x,t,k)$ is differentiable with respect to *x* and *t* is essentially the same as in the case of Problem I. Therefore, we only outline the necessary modifications.

In the case of Problem II, we assume that $d^*(k)$ has no zeros in $\overline{\mathbb {C}^+}$: Zeros in the open upper half-plane can be eliminated by introducing small circular contours and converting the residue conditions into jump conditions, zeros on $\mathbb {R}$ can be eliminated by introducing small half-circular contours.

We then introduce the matrices $v_\pm$ and rewrite the jump matrix in a form suitable for Lemmas 7.1 and 7.2. Namely, $[\check{J}^{(xt)}]^{(-1)}(x,t,k)=(v^-)^{-1}v^+$ with

7.23 $$\begin{aligned} v^-(x,t,k)={\left\{ \begin{array}{ll} \begin{pmatrix} 1& 0\\ -\rho ^*(k)\textrm{e}^{2\textrm{i}kp(x,t,k)}& 1 \end{pmatrix},\quad k\in \mathbb {R}\cap \{|k| >\epsilon \},\\ \begin{pmatrix} 1& \tilde{\rho }(k)\textrm{e}^{-2\textrm{i}kp(x,t,k)}\\ 0& 1 \end{pmatrix},\quad k\in \mathbb {R}\cap \{|k| <\epsilon \},\\ I,\quad k\in \mathbb {C}^+\cap \{|k| =\epsilon \},\\ \begin{pmatrix} {1}& 0\\ \Xi ^*(k)\textrm{e}^{2\textrm{i}kp(x,t,k)}& {1} \end{pmatrix},\quad k\in \mathbb {C}^-\cap \{|k| =\epsilon \} \end{array}\right. } \end{aligned}$$

and

7.24 $$\begin{aligned} v^+(x,t,k)={\left\{ \begin{array}{ll} \begin{pmatrix} 1& \rho (k)\textrm{e}^{-2\textrm{i}kp(x,t,k)}\\ 0& 1 \end{pmatrix},\quad k\in \mathbb {R}\cap \{|k| >\epsilon \},\\ \begin{pmatrix} 1& 0\\ -\rho ^*(k)\textrm{e}^{2\textrm{i}kp(x,t,k)}& 1 \end{pmatrix},\quad k\in \mathbb {R}\cap \{|k| <\epsilon \},\\ \begin{pmatrix} {1}& \Xi (k)\textrm{e}^{-2\textrm{i}kp(x,t,k)}\\ 0& {1} \end{pmatrix},\quad k\in \mathbb {C}^+\cap \{|k| =\epsilon \},\\ I,\quad k\in \mathbb {C}^-\cap \{|k| =\epsilon \}. \end{array}\right. } \end{aligned}$$

Moreover, from the considerations in Sect. 2.1.2, it follows that

7.25 $$\begin{aligned}&\rho (k)=O\left( \frac{1}{k}\right) ,\quad k\in \mathbb {R},~ k\rightarrow \infty ;\quad \rho (k)\in C(\mathbb {R}),\end{aligned}$$

7.26 $$\begin{aligned}&\tilde{\rho }(k)=O(k),\quad k\in \mathbb {R},~ k\rightarrow 0;\quad \tilde{\rho }(k)\in C(\mathbb {R}),\end{aligned}$$

7.27 $$\begin{aligned}&\Xi (k)\in C(\{|k|=\epsilon \}), \quad k\in \mathbb {C}^+. \end{aligned}$$

Further, we proceed in the same way as in the case of Problem I.

## The uniqueness and existence results for Problem I

### Uniqueness result for Problem I

In this section, assuming the existence of a solution to Problem I (i.e., with an appropriate behavior as $x \rightarrow +\infty$), we address its uniqueness.

#### Theorem 8.1

Let *u*(*x*, *t*) be a solution of the sG equation in the domain x>0, 0<t<T such that $u\in C^1([0,T],\tilde{H}^{1,2}((0,\infty )))$. Then, *u*(*x*, *t*) is uniquely determined by *u*(*x*, 0), $x\in [0,\infty )$, through a unique solution of the Riemann–Hilbert problem, for which the data (jump matrix and residue condition) are given in terms of the spectral functions associated with *u*(*x*, 0).

#### Proof

The uniqueness of the solution to the RH problem implies, using (5.6), the following procedure for representing *u*(*x*, *t*) in terms of $$ M^{(xt)}(x,t,k)$$:

Step 1. Given *u*(*x*, 0) construct *a*(*k*), *b*(*k*) (see Sect. 2.1.4).
Step 2. Having *a*(*k*) and *b*(*k*), compute $\kappa _1^0$ and $\kappa _2^0$, and determine $\tilde{a}(k)$ and $\tilde{b}(k)$ via (2.29). With these data, formulate the Riemann–Hilbert problem ${\textbf {RH}}^{(xt)}$, see Sect. 5.1.
Step 3. Solve this RH problem for all x≥0 and t∈[0,T] and evaluate its solution $$ M^{(xt)}(x,t,k)$$ at k=∞:
  8.1 $$\begin{aligned} M^{(xt)}(x,t,k)= I+\frac{1}{4\textrm{i}k}\begin{pmatrix} -\eta (x,t)& \xi (x,t)\\ \xi (x,t)& \eta (x,t) \end{pmatrix}+O\bigg (\frac{1}{k^2}\bigg ). \end{aligned}$$
Step 4. Define $$ u_x(x,t)$$, x≥0 and t∈[0,T], from this expansion as follows (cf. (5.3)):
  8.2 $$\begin{aligned} u_x(x,t)=\xi (x,t). \end{aligned}$$
Step 5. Having $u_{x}(x,t)$, construct *u*(*x*, *t*) by
  8.3 $$\begin{aligned} u(x,t) = -\int _x^{+\infty } u_{x}(y,t)\textrm{d}y+2\pi k, \end{aligned}$$
  where *k* is defined by the behavior of *u*(*x*, 0) as $x\rightarrow +\infty$.

□

### Well-posedness of Problem I: mapping of RH problems

The fact that the initial data on a half-line $x\in [l,+\infty )$ determine uniquely the solution of the sG equation in the corresponding quarter-plane x≥l, t≥0 suggests that the RH problems associated with two initial data, $u_1(x,0)$ for $x\in [l_1,+\infty )$ and $u_2(x,0)$ for $x\in [l_2,+\infty )$ with $l_2<l_1$ such that $u_2(x,0)=u_1(x,0)$ for all $x\in [l_1,+\infty )$, have to be related for $x\in [l_1,+\infty )$, t≥0.

First, assume that $u_1(x,t)$ is a solution of the sG equation on $x\in [l_1,+\infty )$ and $u_2(x,t)$ is a solution of the sG equation on $x\in [l_2,+\infty )$, and establish a useful relation among the spectral functions of the problems, in the framework of the direct problems. In analogy with (2.5b), introduce

8.4 $$\begin{aligned} p_j(x,t,k):=x-l_j-\frac{t}{4 k^2},\qquad j=1,2 \end{aligned}$$

and notice that

8.5 $$\begin{aligned} p_2(x,t,k)-p_1(x,t,k) = l_2-l_1=:\mu >0. \end{aligned}$$

Introduce the Jost solutions $\Phi _{ 2}$, $\Phi _{ 3}$, $\hat{\Phi }_{ 2}$ and $\hat{\Phi }_{ 3}$ as follows: $\Phi _{ 3}$ and $\hat{\Phi }_{ 3}$ are introduced by (2.10), where $U_\infty$ is given by (2.4a) with $u_x$ replaced by $u_{1x}$ and $u_{2x}$, respectively, whereas let $\Phi _{ 2}$ and $\check{\Phi }_{2}$ be the analogs of $\Phi _{ 2}$ introduced by (2.9), taking into account the left ends of the spatial intervals:

8.6a $$\begin{aligned} \Phi _{ 2}(x,t,k)=I&+ \int _{l_1}^{x} \textrm{e}^{-\textrm{i}k(x-y)\hat{\sigma }_3}( U_\infty \Phi _{2})(y,t,k) \textrm{d}y \nonumber \\&+\textrm{e}^{-\textrm{i}k(x-l_1)\hat{\sigma }_3} \int _{0}^{t} \textrm{e}^{\frac{\tau -t}{4 \textrm{i}k}\hat{\sigma }_3}( V_\infty \Phi _{2})(l_1,\tau ,k) \textrm{d}\tau ,\end{aligned}$$

8.6b $$\begin{aligned} \hat{\Phi }_{ 2}(x,t,k)=I&+ \int _{l_2}^{x} \textrm{e}^{-\textrm{i}k(x-y)\hat{\sigma }_3}( U_\infty \hat{\Phi }_{2})(y,t,k) \textrm{d}y \nonumber \\&+\textrm{e}^{-\textrm{i}k(x-l_2)\hat{\sigma }_3} \int _{0}^{t} \textrm{e}^{\frac{\tau -t}{4 \textrm{i}k}\hat{\sigma }_3}( V_\infty \hat{\Phi }_{2})(l_2,\tau ,k) \textrm{d}\tau , \end{aligned}$$

where $U_\infty$ and $V_\infty$ are given by (2.4a) and (2.4b) with *u* replaced by $u_{1}$ and $u_{2}$, respectively.

Introduce the scattering matrices $s^{(j)}(k)$, j=1,2 (with the corresponding entries $a^{(j)}(k)$ and $b^{(j)}(k)$) associated with the initial data $\{u_j(x,0), x\in [l_j,+\infty )\}$, which are determined by the scattering relations similar to (2.15a):

8.7a $$\begin{aligned}&\Phi _{3}(x,t,k)=\Phi _{2}(x,t,k) \textrm{e}^{-\textrm{i}k p_1(x,t,k)\sigma _3}s^{(1)}(k)\textrm{e}^{\textrm{i}k p_1(x,t,k)\sigma _3},\end{aligned}$$

8.7b $$\begin{aligned}&\hat{\Phi }_{ 3}(x,t,k)=\hat{\Phi }_{ 2}(x,t,k)\textrm{e}^{-\textrm{i}k p_2(x,t,k)\sigma _3}s^{(2)}(k)\textrm{e}^{\textrm{i}k p_2(x,t,k)\sigma _3}, \end{aligned}$$

where

8.8a $$\begin{aligned} s^{(1)}(k)=\Phi _{ 3}(l_1,0,k)=:\begin{pmatrix} a^{(1)*}(k) &  b^{(1)}(k)\\ -b^{(1)*}( k) &  a^{(1)}(k) \end{pmatrix} \end{aligned}$$

and

8.8b $$\begin{aligned} s^{(2)}(k)=\hat{\Phi }_{ 3}(l_2,0,k)=:\begin{pmatrix} a^{(2)*}(k) &  b^{(2)}(k)\\ -b^{(2)*}( k) &  a^{(2)}(k) \end{pmatrix}. \end{aligned}$$

It follows that for t=0 and $x\ge l_1$, $\Phi _{ 2}$, and $\hat{\Phi }_{ 2}$ can be related (taking into account (8.5)) by

8.9 $$\begin{aligned} \hat{\Phi }_{ 2}(x,0,k) = \Phi _{2}(x,0,k) \textrm{e}^{-\textrm{i}k p_1(x,0,k)\sigma _3}s^{(1)}(k) \textrm{e}^{-\textrm{i}k \mu \sigma _3}[s^{(2)}]^{-1}(k) \textrm{e}^{\textrm{i}k p_2(x,0,k)\sigma _3}. \end{aligned}$$

Now, we notice that by construction, the first column of $\hat{\Phi }_{\infty 2}(x,t,k)$ is analytic in $\mathbb {C}^+$ and, moreover, its (21) entry is *O*(1/*k*) as $k\rightarrow \infty$. On the other hand, the (21) entry of the r.h.s. of (8.9) evaluated at $x=l_1$ reads in terms $a^{(j)}$ and $b^{(j)}$ as $-b^{(1)*}(k)a^{(2)}(k) + b^{(2)*}(k)a^{(1)}(k)\textrm{e}^{2\textrm{i}k \mu }$. Consequently, we have

8.10 $$\begin{aligned} -b^{(1)*}(k)a^{(2)}(k) + b^{(2)*}(k)a^{(1)}(k)\textrm{e}^{2\textrm{i}k\mu } = O\left( \frac{1}{k}\right) , \qquad k\rightarrow \infty , \quad k\in {\mathbb {C}}^+. \end{aligned}$$

#### Remark 8.2

In general, the spectral functions $b^{(j)*}(k)$ are determined for $k\in {\mathbb {C}}^-$, but their combination as in the l.h.s. of (8.10) turns out to be analytically continued into ${\mathbb {C}}^+$.

#### Theorem 8.3

Let $M^{(2)}(x,t,k)$, $x\in [l_2, +\infty )$, t≥0 satisfy the RH problem ${\textbf {RH}}^{(xt)}$ with *p*(*x*, *t*, *k*) replaced by $p_2(x,t,k)$ and the spectral functions $a^{(2)}(k)$, $b^{(2)}(k)$ associated with the initial data *u*(*x*, 0), $x\in [l_2, +\infty )$. For $l_1>l_2$, define $\tilde{M}^{(1)}(x,t,k)$ for $x\in [l_1, +\infty )$, t≥0 as follows:

8.11 $$\begin{aligned} \tilde{M}^{(1)}(x,t,k):= &   M^{(2)}(x,t,k) \nonumber \\  &   {\left\{ \begin{array}{ll} \begin{pmatrix} 1 &  0 \\ X^{(1)}(k)\textrm{e}^{2\textrm{i}k p_1(x,t,k)} - X^{(2)}(k)\textrm{e}^{2\textrm{i}k p_2(x,t,k)} &  1 \end{pmatrix}, &  k\in {\mathbb {C}}^+, \\ \begin{pmatrix} 1 &  X^{(2)*}(k)\textrm{e}^{-2\textrm{i}k p_2(x,t,k)} - X^{(1)*}(k)\textrm{e}^{-2\textrm{i}k p_1(x,t,k)} \\ 0 &  1 \end{pmatrix}, &  k\in {\mathbb {C}}^-, \\ \end{array}\right. } \end{aligned}$$

where

8.12 $$\begin{aligned} X^{(j)}(k) = {\left\{ \begin{array}{ll} r^{(j)}(k), &  k\in {\mathbb {C}}^+\cap \{|k|>\epsilon \} \\ r^{(j)}(k) - \frac{\kappa _2^{(0j)}}{a^{(j)}(k) \tilde{a}^{(j)}(k)}, &  k\in {\mathbb {C}}^+\cap \{|k|<\epsilon \} \end{array}\right. } \end{aligned}$$

with $$r^{j}(k)=\frac{b^{(j)*}(k)}{a^{(j)}(k)}$$ and $$\kappa _2^{(0j)}=b^{(j)}(0)$$.

Then for $x\in [l_1, +\infty )$ and t≥0, $\tilde{M}^{(1)}(x,t,k) = M^{(1)}(x,t,k)$, where $M^{(1)}(x,t,k)$ satisfies the RH problem ${\textbf {RH}}^{(xt)}$ associated with the initial data *u*(*x*, 0) restricted to $x\in [l_1, +\infty )$.

#### Proof

In view of the construction of RH problems and the way of extracting the solution of the sG equation from the solution of the associated RH problem, it is sufficient to show that for $k\in {\mathbb {C}}^+$ and for all $x\ge l_1$, t≥0,

(i) $$X^{(1)}(k)\textrm{e}^{2\textrm{i}k p_1(x,t,k)} - X^{(2)}(k)\textrm{e}^{2\textrm{i}k p_2(x,t,k)} = O(1/k) $$ as $$k\rightarrow \infty $$;
(ii) $$X^{(1)}(k)\textrm{e}^{2\textrm{i}k p_1(x,t,k)} - X^{(2)}(k)\textrm{e}^{2\textrm{i}k p_2(x,t,k)} = O(k) $$ as k→0.

Regarding item (i), notice that for $k\in {\mathbb {C}}^+\cap \{|k|>\epsilon \}$,

$$\begin{aligned}  &   X^{(1)}(k)\textrm{e}^{2\textrm{i}k p_1(x,t,k)} - X^{(2)}(k)\textrm{e}^{2\textrm{i}k p_2(x,t,k)} = \frac{e^{2\textrm{i}k p_1(x,t,k)}}{a^{(1)}(k)a^{(2)}(k)} \\  &   \quad \left( b^{(1)*}(k)a^{(2)}(k) - b^{(2)*}(k)a^{(1)}(k)\textrm{e}^{2\textrm{i}k\mu }\right) \end{aligned}$$

and thus, taking into account (8.10), the analyticity of $a^{(j)}(k)$ in ${\mathbb {C}}^+$ with $a^{(j)}(k)=1+O\left( \frac{1}{k}\right)$, and that $\operatorname {Im}p_1(x,t,k)\ge 0$ for all $x\ge l_1$, t≥0, the statement of (i) follows.

Regarding item (ii), we notice that the estimate $X^{(1)}(k)\textrm{e}^{2\textrm{i}k p_1(x,t,k)} - X^{(2)}(k)\textrm{e}^{2\textrm{i}k p_2(x,t,k)}=O(k)$ follow from (2.42), the estimates $\tilde{a}^{(j)}(k) = 1 + O(k)$ as k→0, and the fact that $X^{(1)}(k)\textrm{e}^{2\textrm{i}k p_1(x,t,k)} - X^{(2)}(k)\textrm{e}^{2\textrm{i}k p_2(x,t,k)}$ is analytic in ${\mathbb {C}}^+$ including near k=0 (see Remark 8.2).

□

### Behavior as $x\rightarrow +\infty$

In Sects. 8.1 and 8.2, we assumed the existence of a solution to Problem I for the sine-Gordon equation (with prescribed behavior as $x \rightarrow +\infty$) and constructed the associated Riemann–Hilbert problem from the corresponding Jost solutions. In the present and the following section, we drop this assumption and instead analyze the Riemann–Hilbert problem constructed from initial data belonging to a suitable class. The differentiability of the ingredients extracted from this Riemann–Hilbert problem, which are used in the reconstruction of the solution to the sine-Gordon equation, was established in Sect. 7.2. In this section, we focus on the behavior as $x \rightarrow +\infty$. To fix ideas, we assume that *a*(*k*) has no zeros in $\overline{\mathbb {C}^+}$..

First, notice that the considerations in Sect. 8.2 imply that extending the initial data to the left does not affect the solution of the problem on the common half-line. In particular, one may extend the domain (0,+∞) to $(-\infty ,+\infty )$, thus reducing Problem I to a problem on the whole real line. Consequently, without loss of generality, we can assume that $\kappa _2 = 0$, $r(k)=O\left( \frac{1}{k^2}\right)$ as $k\rightarrow \infty$ (i.e., $r(k), ~kr(k) \in L^{2}((0,\infty ))$), and $r(k) = O(k^3)$ as k→0. To ensure the last condition, we extend the initial function $u_0(x)$ from (0,+∞) to $(-\infty ,+\infty )$ in such a way that $\int _{-\infty }^{\infty } \sin u_0(y)\textrm{d}y=0$ and $\int _{-\infty }^{\infty } y\sin u_0(y)\textrm{d}y=0$.

The dominated convergence theorem, together with the properties of *r*(*k*), implies that we can pass to the (non-tangential) limits $k\rightarrow \infty$ and k→0 in (7.13). In particular, we have

8.13 $$\begin{aligned} \begin{aligned} \xi (x,t)=&\frac{2}{\pi }\int _{\mathbb {R}} \mu _{11}^{(xt)}(x,t,z) r^*(z)\textrm{e}^{-2\textrm{i}z x+\frac{\textrm{i}t}{2z}} \textrm{d}z, \end{aligned} \end{aligned}$$

8.14 $$\begin{aligned} \begin{aligned} \beta (x,t)=&\frac{1}{2\pi \textrm{i}}\int _{\mathbb {R}} \mu _{11}^{(xt)}(x,t,z) \frac{r^*(z)}{z}\textrm{e}^{-2\textrm{i}z x+\frac{\textrm{i}t}{2z}} \textrm{d}z. \end{aligned} \end{aligned}$$

Notice that the following analogs of Lemma 2.3 and Theorem 2.1 from [26] hold:

#### Lemma 8.4

Assume that $r(k)\in H^n(\mathbb {R})$ and *r*(*k*) vanishes to order at least 2*n* at k=0. Then

8.15 $$\begin{aligned} \Vert C_- w^+\Vert _{L^2}, ~\Vert C_+ w^-\Vert _{L^2}\le \frac{\Vert r\Vert _{H^n}}{(1+x^2)^{\frac{n}{2}}}. \end{aligned}$$

#### Proof

We prove the estimate for $C_- w^+$; the argument for $C_+ w^-$ is analogous.

First, notice that under the assumptions on *r*(*k*), it follows that

$$\begin{aligned} g(k):=r(k)\textrm{e}^{-\frac{\textrm{i}t}{2 k}}\in H^n(\mathbb {R}) \end{aligned}$$

Observe that for a sufficiently nice function *f* (i.e., $f\in L^2(\mathbb {R})$), we have

$$ (C_- f)(k)=\lim _{\epsilon \downarrow 0} \frac{1}{2\pi \textrm{i}}\int _{\mathbb {R}} \frac{f(s)}{s-(k-\textrm{i}\epsilon )}\textrm{d}s. $$

Furthermore, for $f\in L^2(\mathbb {R})$, the following holds

$$ f(k)=\frac{1}{\sqrt{2\pi }}\int _{\mathbb {R}} \textrm{e}^{\textrm{i}k\xi }\hat{f}(\xi )\textrm{d}\xi ,\quad \hat{f}(\xi )=\frac{1}{\sqrt{2\pi }}\int _{\mathbb {R}} \textrm{e}^{-\textrm{i}k\xi } f(k)\textrm{d}k $$

and

$$ (C f)(z)=\frac{1}{2\pi \textrm{i}}\int _{\mathbb {R}} \frac{f(s)}{s-z}\textrm{d}s=\frac{1}{\sqrt{2\pi }}\int _{\mathbb {R}}\left( \frac{1}{2\pi \textrm{i}}\int _{\mathbb {R}} \frac{\textrm{e}^{\textrm{i}s\xi }}{s-z}\textrm{d}s\right) \hat{f}(\xi )\textrm{d}\xi . $$

For $z\in \mathbb {C}^-$, the Jordan’s lemma implies

$$ \frac{1}{2\pi \textrm{i}}\int _{\mathbb {R}} \frac{\textrm{e}^{\textrm{i}s\xi }}{s-z}\textrm{d}s={\left\{ \begin{array}{ll} \textrm{e}^{\textrm{i}z\xi },\quad \xi <0,\\ 0,\quad \xi >0, \end{array}\right. } $$

Thus,

$$ (C f)(z)=\frac{1}{\sqrt{2\pi }}\int _{-\infty }^0 \textrm{e}^{\textrm{i}z\xi }\hat{f}(\xi )\textrm{d}\xi , $$

and

8.16 $$\begin{aligned} (C_- f)(k)=\frac{1}{\sqrt{2\pi }}\int _{-\infty }^0 \textrm{e}^{\textrm{i}k\xi }\hat{f}(\xi )\textrm{d}\xi ,\quad \widehat{(C_- f)}(\xi )=\chi _{(-\infty ,0)}\hat{f}(\xi ). \end{aligned}$$

Applying (8.16) to $f(k):=g(k)\textrm{e}^{2\textrm{i}k x}$ and using $\hat{f}(\xi )=\hat{g}(\xi -2x)$, we obtain

$$ (C_- f)(k)=\frac{1}{\sqrt{2\pi }}\int _{-\infty }^0 \textrm{e}^{\textrm{i}k\xi }\hat{r}(\xi -2x)\textrm{d}\xi =\frac{\textrm{e}^{2\textrm{i}k x}}{\sqrt{2\pi }}\int _{-\infty }^{-2x} \textrm{e}^{\textrm{i}k\eta }\hat{r}(\eta )\textrm{d}\eta , $$

and, using Plancherel’s theorem and (8.16), we obtain

8.17 $$\begin{aligned} \begin{aligned}&\Vert C_- f\Vert _{L^2}^2= \Vert \widehat{C_- f}\Vert _{L^2}^2=\int _{-\infty }^0 |\hat{g}(\xi -2x)|^2\textrm{d}\xi =\int _{-\infty }^{-2x}|\hat{g}(\eta )|^2\textrm{d}\eta \\&=\int _{-\infty }^{-2x} \frac{1}{(1+\eta ^2)^n}(1+\eta ^2)^n|\hat{g}(\eta )|^2\textrm{d}\eta \le \frac{\Vert r\Vert _{H^n}}{(1+ x^2)^n} \end{aligned} \end{aligned}$$

□

#### Lemma 8.5

Assume that $r(k)\in H^n(\mathbb {R})$ and *r*(*k*) vanishes to order at least 2*n* at k=0. Then, for all fixed *t*

8.18 $$\begin{aligned} \xi (x,t)\in L^2((1+x^{2n})\textrm{d}x,(0,+\infty )). \end{aligned}$$

Assume additionally that *r*(*k*) vanishes to order at least 2n+1 at k=0. then

8.19 $$\begin{aligned} \beta (x,t)\in L^2((1+x^{2n})\textrm{d}x,(0,+\infty )). \end{aligned}$$

#### Proof

First, consider (8.13).

Notice that

$$ \int _{\mathbb {R}} \mu _{11}^{(xt)}(x,t,z) r^*(z)\textrm{e}^{-2\textrm{i}z x+\frac{\textrm{i}t}{2z}} \textrm{d}z=\int _{\mathbb {R}} \left( (1\!\!1-C_{w(x,t,z)})^{-1}I\right) _{11} r^*(z)\textrm{e}^{-2\textrm{i}z x+\frac{\textrm{i}t}{2z}} \textrm{d}z, $$

and

$$ (1\!\!1-C_{w})^{-1}I=I+C_{w}I+C_{w}(1\!\!1-C_{w})^{-1}C_{w}I,\quad C_{w}(1\!\!1-C_{w})^{-1}C_{w}I=C_{w}(\mu -I). $$

Thus, we can split the integral into three parts:

$$ \int _{\mathbb {R}} \left( (1\!\!1-C_{w(x,t,z)})^{-1}I\right) _{11} r^*(z)\textrm{e}^{-2\textrm{i}z x+\frac{\textrm{i}t}{2z}} \textrm{d}z=I_1+I_2+I_3 $$

with

$$\begin{aligned}&I_1=\int _{\mathbb {R}} r^*(z)\textrm{e}^{-2\textrm{i}z x+\frac{\textrm{i}t}{2z}} \textrm{d}z,\\&I_2=\int _{\mathbb {R}} \left( C_{w}I\right) _{11}r^*(z)\textrm{e}^{-2\textrm{i}z x+\frac{\textrm{i}t}{2z}} \textrm{d}z,\\&I_3=\int _{\mathbb {R}} \left( C_{w}(\mu -I)\right) _{11}r^*(z)\textrm{e}^{-2\textrm{i}z x+\frac{\textrm{i}t}{2z}} \textrm{d}z \end{aligned}$$

Consider $I_1$. Notice that under the assumptions on *r*(*k*), it follows that

$$\begin{aligned} g(k):=r(k)\textrm{e}^{-\frac{\textrm{i}t}{2 k}}\in H^n(\mathbb {R}), \end{aligned}$$

and thus,

$$ I_1\in L^2((1+x^{2n})\textrm{d}x,(0,+\infty )) $$

by the properties of the Fourier transform.

Consider $I_2$. First, notice that the fact that $w_\pm$ are nilpotent implies

$$ C_{w}I(w^++w^-)=(C_+w^-)w^++(C_-w^+)w^-. $$

Furthermore, $$C_+-C_-=1\!\!1$$ yields

$$\begin{aligned}  &   (C_+w^-)w^++(C_-w^+)w^-=-(C_+w^-)(C_-w^+)+(C_-w^+)(C_+w^-)\\  &   \quad +(C_+w^-)(C_+w^+)-(C_-w^+)(C_-w^-). \end{aligned}$$

Since $(C_+w^-)(C_+w^+)$ is analytic in the upper half-plane, while $(C_-w^-)(C_-w^+)$ is analytic in the lower half-plane, Cauchy’s theorem implies that

$$\int _{\mathbb {R}}(C_+w^-)(C_+w^+) \textrm{d}z=0,\quad \int _{\mathbb {R}}(C_-w^+)(C_-w^-)\textrm{d}z=0. $$

Now, applying Lemma 8.4 together with Cauchy–Schwarz’s inequality, we get

$$ |I_2|\le \int _{\mathbb {R}} |((C_+w^-)(C_-w^+))_{11}|+|((C_-w^+)(C_+w^-))_{11}|\textrm{d}z\le \frac{C}{1+x^{2n}} $$

Therefore,

$$ \xi (x,t)\in L^2((1+x^{2n})\textrm{d}x,(0,+\infty )). $$

Notice that under the assumptions on *r*(*k*), it follows that

$$\begin{aligned} \frac{r(k)}{k}\textrm{e}^{-\frac{\textrm{i}t}{2 k}}\in H^n(\mathbb {R}), \end{aligned}$$

thus (8.19) follows.

□

Applying Lemma 8.4 and Lemma 8.5 in the case n=0, we obtain the following

#### Corollary 8.6

Assume that $u_{0}(x)\in H^{1,2}((0,\infty ))+2\pi k$. Then for all fixed t≥0

8.20 $$\begin{aligned} \xi (x,t),~\beta (x,t)\in L^2((0,+\infty )) \end{aligned}$$

Moreover, assuming $u_{0x}(x)\in H^{1,2}((0,+\infty ))$ and proceeding as in [24], we can prove that $\frac{\textrm{d}}{\textrm{d}k}r(k)\in L^2(\mathbb {R})$. Therefore, applying Lemma 8.4 and Lemma 8.5 in the case n=1, we have

#### Corollary 8.7

Assume that $u_{0}(x)\in H^{2,2}((0,+\infty ))+2\pi k$. Then for all fixed t≥0

8.21 $$\begin{aligned} \xi (x,t),~\beta (x,t)\in L^2((1+x^{2})\textrm{d}x,(0,+\infty )). \end{aligned}$$

### Existence result for Problem I

#### Theorem 8.8

Let $u_0(x)\in H^{2,2}((0,+\infty ))+2\pi k$ with some $k\in \mathbb {Z}$ and let *a*(*k*) be the spectral function associated with $u_0$ through the direct *x*-spectral problem. Assume that *a*(*k*) has no zeros in $\overline{\mathbb {C}^+}$.

Then the problem

8.22a $$\begin{aligned}&u_{xt} = \sin u,\quad \quad x \ge 0,\quad 0\le t< T<\infty ;\end{aligned}$$

8.22b $$\begin{aligned}&u(x,0) = u_0(x), \quad x \ge 0;\end{aligned}$$

8.22c $$\begin{aligned}&u(\cdot ,t)\in \tilde{H}^{1,2}((0,+\infty )), \quad 0\le t< T<\infty \end{aligned}$$

has a unique solution, *u*(*x*, *t*), which can be represented in terms of the solution of the associated RH problem ${\textbf {RH}}^{(xt)}$ via (8.2)–(8.3).

#### Proof

As it was shown above, the existence of a solution of the RH problem ${\textbf {RH}}^{(xt)}$ follows from Zhou’s vanishing lemma. Next, we construct *u*(*x*, *t*) by following Steps 2–4 of Proposition 8.1. As shown in Sect. 6, the function *u*(*x*, *t*) constructed in this way indeed satisfies the sG equation. Moreover, the considerations of Sect. 7.2 imply that *u*(*x*, *t*) is $C^1$ with respect to both variables *x* and *t*, whereas Sect. 8.3 establishes that $u(x,t)\in \tilde{H}^{1,2}((0,+\infty ))$.

Therefore, it remains to prove that $u(x,0)=u_0(x)$ for all x≥0. Notice that ${ M}^{(xt)}(x,0,k)$ satisfies the RH problem ${\textbf {RH}}^{(x)}$, see Sect. 4.1.1. Thus by uniqueness of solution of RH problem ${\textbf {RH}}^{(x)}$, we conclude that $\hat{ M}^{(xt)}(y,0,k)=\hat{ M}^{(x)}(y,k)$. In particular,

$$ {u_x}(x,0)={u}_{0x}(x). $$

From the solution of the Riemann–Hilbert problem, we extract the quantities sinu(x,t), cosu(x,t), and $u_x(x,t)$. Hence, the function *u*(*x*, *t*) can be recovered up to an additive constant of the form 2πk, $k \in \mathbb {Z}$. The constant is then uniquely determined by imposing the initial condition, together with continuity of the reconstructed solution. □

#### Remark 8.9

The assumption that *a*(*k*) has no zeros in $\overline{\mathbb {C}^+}$ is made in order to focus on the main ideas and avoid additional technical details. Zeros in the open upper half-plane can be handled by introducing small circular contours and converting the corresponding residue conditions into jump conditions, while zeros on $\mathbb {R}$ can be treated by introducing small half-circular contours.

## The uniqueness and existence results for Problem II

### Uniqueness result for Problem II

Similarly to Problem I, in this section, assuming the existence of a solution to Problem II (with prescribed behavior as $x \rightarrow +\infty$), we address its uniqueness.

#### Theorem 9.1

Let *u*(*x*, *t*) be a solution of the sG equation in the domain x<0, 0<t<T such that $u\in C^1([0,T],\tilde{H}^{1,2}((-\infty ,0)))$. Then, *u*(*x*, *t*) is uniquely determined by *u*(*x*, 0), $x\in (-\infty ,0]$, and *u*(0, *t*), 0<t<T, through a unique solution of the Riemann–Hilbert problem, for which the data (jump matrix and residue condition) are given in terms of spectral functions associated with *u*(*x*, 0) and *u*(0, *t*).

#### Proof

The uniqueness of the solution to the RH problem implies, using (5.17), the following procedure for representing *u*(*x*, *t*) in terms of $\check{M}^{(xt)}(x,t,k)$:

Step 1. Given *u*(*x*, 0) construct *a*(*k*), *b*(*k*) (see Sect. 2.1.4); given *u*(0, *t*) construct *A*(*k*) and *B*(*k*) (see Sect. 2.1.5).
Step 2. Having *a*(*k*), *b*(*k*) compute $\kappa _1^0$, $\kappa _2^0$, and determine $\tilde{a}(k)$ and $\tilde{b}(k)$ via (2.29); having *A*(*k*) and *B*(*k*) compute $\kappa _i^0$, $\kappa _i^T$, i=1,2, and determine $\tilde{A}(k)$ and $\tilde{B}(k)$ via (2.30). With these data, formulate the Riemann–Hilbert problem , see Sect. 5.2.;
Step 3. Solve this RH problem for all x≤0 and t∈[0,T] and evaluate its solution $\check{M}^{(xt)}(x,t,k)$ at k=∞:
  9.1 $$\begin{aligned} \check{M}^{(xt)}(x,t,k)= I+\frac{1}{4\textrm{i}k}\begin{pmatrix} -\eta (x,t)& \xi (x,t)\\ \xi (x,t)& \eta (x,t) \end{pmatrix}+O(\frac{1}{k^2}). \end{aligned}$$
Step 4. Define $$ u_x(x,t)$$, x≤0 and t∈[0,T], from this expansion as follows (cf. (5.14)):
  9.2 $$\begin{aligned} u_x(x,t)=\xi (x,t). \end{aligned}$$
Step 5. Having $u_{x}(x,t)$, construct *u*(*x*, *t*) by
  9.3 $$\begin{aligned} u(x,t) = \int ^x_{ -\infty } u_{x}(y,t)\textrm{d}y+2\pi k=u(0,t)+ \int _0^x u_{x}(y,t)\textrm{d}y, \end{aligned}$$
  where *k* is defined by the behavior of *u*(*x*, 0) as $x\rightarrow -\infty$.

□

### Behavior as $x\rightarrow -\infty$

In Sect. 9.1, the Riemann–Hilbert problem was derived under the assumption that a solution to Problem II for the sine-Gordon equation exists and satisfies prescribed asymptotic conditions as $x \rightarrow -\infty$. We now reverse this perspective: Instead of starting from a solution of the sine-Gordon equation, we take initial data from an appropriate class and study the corresponding Riemann–Hilbert problem directly. The regularity properties of the quantities involved in the reconstruction procedure were established in Sect. 7.3. The aim of the present section is to analyze the behavior of the reconstructed solution as $x \rightarrow -\infty$. To fix ideas, we assume that $d^*(k)$ has no zeros in $\overline{\mathbb {C}^+}$.

First, we construct *h* and $\tilde{h}$ to improve the behavior at k=∞ and k=0, and to achieve the matching near k=ϵ (c.f. [24]).

Let

$$ h_0(k)=\frac{\rho _1}{\textrm{i}k}, \qquad \tilde{h}_0(k)=\textrm{i}\tilde{\rho }_1 k-\tilde{\rho }_2k^2, $$

where

$$ \rho (k)=\frac{\rho _1}{\textrm{i}k}+O\left( \frac{1}{k^2}\right) , \qquad \tilde{\rho }(k)=\textrm{i}\tilde{\rho }_1 k-\tilde{\rho }_2k^2+O(k^3). $$

Define

$$ F(k)=\Xi (k)+h_0(k)-\tilde{h}_0(k), $$

which is analytic in $\mathbb {C}^+\cap \{|k-\epsilon |<\epsilon /2\}$. For n≥1, set

$$ G(k)=(k+\textrm{i})^{n+2}F(k), \qquad T_nG(k)=\sum _{j=0}^n \frac{G^{(j)}(\epsilon )}{j!}(k-\epsilon )^j, $$

and

$$ H_0(k)=\frac{T_nG(k)}{(k+\textrm{i})^{n+2}}, \qquad H(k)=\tfrac{1}{2}\big (H_0(k)+\overline{H_0(-\bar{k})}\big ). $$

Then

$$ h(k)=\frac{\rho _1}{\textrm{i}k}+H(k), \qquad \tilde{h}(k)=\textrm{i}\tilde{\rho }_1 k-\tilde{\rho }_2k^2 $$

satisfy $f(k)=\overline{f(-\bar{k})}$, are continuous on |k|=ϵ. Moreover,

$$ h(k)=O\left( \frac{1}{k}\right) ,\ k\rightarrow \infty , \qquad \tilde{h}(k)=O(k),\ k\rightarrow 0, $$

$$ h(k)-\rho (k)=O\left( \frac{1}{k^2}\right) ,\ k\rightarrow \infty , \qquad \tilde{h}(k)-\tilde{\rho }(k)=O(k^3),\ k\rightarrow 0, $$

and

$$ h(k)-\tilde{h}(k)+\Xi (k) =O\big ((k-\epsilon )^n\big ), \quad k\rightarrow \epsilon . $$

Furthermore, since $\rho (k)-\tilde{\rho }(k)+ \Xi (k)=0$ for $k\in \mathbb {R}$, we also have

$$ h(k)-\rho (k)=-\tilde{\rho }(k) + \tilde{h}(k)+O\big ((k-\epsilon )^n\big ), \quad k\rightarrow \epsilon . $$

Introduce a matrix-valued function $\check{\check{M}}(x,t,k)$ related to $\check{M}(x,t,k)$ by

9.4 $$\begin{aligned} \check{\check{M}}(x,t,k)={\left\{ \begin{array}{ll} \check{M}(x,t,k)\textrm{e}^{-\textrm{i}k p(x,t,k)\sigma _3}\begin{pmatrix} 1& -h(k)\\ 0& 1 \end{pmatrix}\textrm{e}^{\textrm{i}k p(x,t,k)\sigma _3},\quad \{|k|>\epsilon \}\cap \mathbb {C}_+,\\ \check{M}(x,t,k)\textrm{e}^{-\textrm{i}k p(x,t,k)\sigma _3}\begin{pmatrix} 1& 0\\ h^*(k)& 1 \end{pmatrix}\textrm{e}^{\textrm{i}k p(x,t,k)\sigma _3},\quad \{|k|>\epsilon \}\cap \mathbb {C}_-,\\ \check{M}(x,t,k)\textrm{e}^{-\textrm{i}k p(x,t,k)\sigma _3}\begin{pmatrix} 1& -\tilde{h}(k)\\ 0 & 1 \end{pmatrix}\textrm{e}^{\textrm{i}k p(x,t,k)\sigma _3},\quad \{|k|<\epsilon \}\cap \mathbb {C}_+,\\ \check{M}(x,t,k)\textrm{e}^{-\textrm{i}k p(x,t,k)\sigma _3}\begin{pmatrix} 1& 0\\ \tilde{h}^*(k)& 1 \end{pmatrix}\textrm{e}^{\textrm{i}k p(x,t,k)\sigma _3},\quad \{|k|<\epsilon \}\cap \mathbb {C}_- \end{array}\right. } \end{aligned}$$

and conclude that it satisfies the following RH problem:

1. Jump condition across $\mathbb {R}\cup \{|k|=\epsilon \}$
  9.5a $$\begin{aligned} \check{\check{M}}_-^{(xt)}(x,t,k)= \check{\check{M}}_+^{(xt)}(x,t,k)\check{\check{J}}^{(xt)}(x,t,k),\quad k\in \mathbb {R}\cup \{|k|=\epsilon \} \end{aligned}$$
  where
  9.5b $$\begin{aligned} \check{\check{J}}^{(xt)}(x,t,k)=\textrm{e}^{-\textrm{i}kp(x,t,k)\sigma _3} \check{\check{J}}^{(xt)}_0(k)\textrm{e}^{\textrm{i}kp(x,t,k)\sigma _3} \end{aligned}$$
  with $$p(x,t,k)=x-\frac{t}{4 k^2}$$ and
  9.5c $$\begin{aligned} \check{\check{J}}^{(xt)}_0(k)={\left\{ \begin{array}{ll} \begin{pmatrix} 1& h(k)-\rho (k)\\ 0& 1 \end{pmatrix} \begin{pmatrix} 1& 0\\ h^*(k)-\rho ^*(k)& 1 \end{pmatrix},\quad k\in \mathbb {R}\cap \{|k| >\epsilon \},\\ \begin{pmatrix} 1& 0\\ \tilde{\rho }^*(k)-\tilde{h}^*(k)& 1 \end{pmatrix}\begin{pmatrix} 1& \tilde{\rho }(k)-\tilde{h}(k)\\ 0& 1 \end{pmatrix},\quad k\in \mathbb {R}\cap \{|k| <\epsilon \},\\ \begin{pmatrix} 1& h(k)-\tilde{h}(k)+\Xi (k)\\ 0& 1 \end{pmatrix},\quad k\in \mathbb {C}^+\cap \{|k| =\epsilon \},\\ \begin{pmatrix} 1& h^*(k)-\tilde{h}^*(k)+\Xi ^*(k)\\ 0& 1 \end{pmatrix},\quad k\in \mathbb {C}^-\cap \{|k| =\epsilon \}. \end{array}\right. } \end{aligned}$$
  Recall that
  $$ \rho (k)-\tilde{\rho }(k)+ \Xi (k)=0,\quad k\in \mathbb {R}. $$
2. Behavior at ∞:
  9.6 $$\begin{aligned} \check{\check{M}}^{(xt)}(x, t,k)=I+O\left( \frac{1}{ k}\right) ,\quad k\rightarrow \infty . \end{aligned}$$

Notice that

9.7 $$\begin{aligned} \check{\check{M}}(x,t,k)=I+\frac{1}{2\pi \textrm{i}}\int _\Sigma \frac{\mu (x,t,z)(\check{w}^+ + \check{w}^-)(x,t,z)}{z-k}dz. \end{aligned}$$

In particular, the properties of *h* and $\tilde{h}$ together with the dominated convergence theorem (limits are not tangential) imply that

9.8 $$\begin{aligned} \begin{aligned} \xi (x,t)=&\frac{2}{\pi }\int _{\mathbb {R}\setminus (-\epsilon ,\epsilon )} \mu _{11}(x,t,z) (h(z)-\rho (z))\textrm{e}^{-2\textrm{i}z x+\frac{\textrm{i}t}{2z}} \textrm{d}z-\\&\frac{2}{\pi }\int _{(-\epsilon ,\epsilon )} \mu _{11}(x,t,z) (\tilde{\rho }(z)-\tilde{h}(z))\textrm{e}^{-2\textrm{i}z x+\frac{\textrm{i}t}{2z}} \textrm{d}z -\\&\frac{2}{\pi }\int _{|z|=\epsilon , z\in \mathbb {C}^+} \mu _{11}(x,t,z) (h(k)-\tilde{h}(k)+ \Xi (k))\textrm{e}^{-2\textrm{i}z x+\frac{\textrm{i}t}{2z}} \textrm{d}z. \end{aligned} \end{aligned}$$

9.9 $$\begin{aligned} \begin{aligned} \beta (x,t)=&\frac{1}{2\pi \textrm{i}}\int _{\mathbb {R}\setminus (-\epsilon ,\epsilon )} \mu _{11}(x,t,z) \frac{(h(z)-\rho (z))}{z}\textrm{e}^{-2\textrm{i}z x+\frac{\textrm{i}t}{2z}} \textrm{d}z-\\&\frac{2}{\pi }\int _{(-\epsilon ,\epsilon )} \mu _{11}(x,t,z) \frac{(\tilde{\rho }(z)-\tilde{h}(z))}{z}\textrm{e}^{-2\textrm{i}z x+\frac{\textrm{i}t}{2z}} \textrm{d}z -\\&\frac{2}{\pi }\int _{|z|=\epsilon , z\in \mathbb {C}^+} \mu _{11}(x,t,z) \frac{(h(k)-\tilde{h}(k)+ \Xi (k))}{z}\textrm{e}^{-2\textrm{i}z x+\frac{\textrm{i}t}{2z}} \textrm{d}z. \end{aligned} \end{aligned}$$

Again, following the ideas of [26] (c.f. Lemma 2.9), we conclude that the first two terms in (9.8)–(9.9) can be treated in the same way as in 8.4 and 8.5, and yield the desired $L^2$-bounds. As for the third term, since the integration is over the upper semicircle and x≤0, the exponential factor $\textrm{e}^{(-2ikx+\tfrac{it}{2k})}$ decays exponentially fast as $x\rightarrow -\infty$ on the contour away from the real line. In addition, the choice of *h* and $\tilde{h}$ ensures that $h-\tilde{h}+\Xi$ vanishes to the required order at the points where the arc intersects the real axis. It follows that this contribution is also in $L^2$. Therefore, we obtain the following result

#### Corollary 9.2

Assume that $u_0(x)\in H^{2,2}((-\infty ,0))+2\pi k$. Then, for all fixed t≥0

9.10 $$\begin{aligned} \xi (x,t),~\beta (x,t)\in L^2((1+x^{2})\textrm{d}x,(-\infty ,0)). \end{aligned}$$

### Existence result for Problem II

#### Theorem 9.3

Let $\{u_0(x)\in H^{2,2}((-\infty ,0))+2\pi k; ~v_0(t)\in H^{1}(0, T)\}$ be a set of functions satisfying internal compatibility condition $u_0(0)=v_0(0)$. Let *a*(*k*), *b*(*k*), *A*(*k*), *B*(*k*) be the spectral functions associated with $u_0$ and $v_0$, and define

$$ d(k)=a(k)A^*(k)+b(k)B^*(k). $$

Assume that $d^*(k)$ has no zeros in $\overline{\mathbb {C}^+}$.

Then the IBVP

9.11a $$\begin{aligned}&u_{xt} = \sin u,\end{aligned}$$

9.11b $$\begin{aligned}&u(0,t) = v_0(t), \quad 0\le t< T<\infty ;\end{aligned}$$

9.11c $$\begin{aligned}&u(x,0) = u_0(x), \quad x \le 0;\end{aligned}$$

9.11d $$\begin{aligned}&u(\cdot ,t)\in \tilde{H}^{1,2}((-\infty ,0)), \quad 0\le t< T<\infty . \end{aligned}$$

has a unique solution, *u*(*x*, *t*), which can be represented in terms of the solution of the associated RH problem  via (9.2)–(9.3).

#### Proof

The existence of a solution of the RH problem  follows from Zhou’s vanishing lemma. Furthermore, Sect. 6 shows that *u*(*x*, *t*) satisfies the sG equation, while Sect. 7.3 implies that *u*(*x*, *t*) is $C^1$ in both *x* and *t*, and Sect. 9.2 establishes that $u(x,t)\in \tilde{H}^{1,2}((-\infty ,0))$.

Therefore, it remains to prove that:

1. $$u(x,0)=u_0(x)$$.
2. $$ u(0,t) = v_0(t)$$.

**1.** Following the approach used for the right half-line problem, we establish a relation between the solution $\check{ M}^{(xt)}(x,t,k)$ of the Riemann–Hilbert problem  problem evaluated at t=0 and the solution $\check{M}^{(x)}(x,k)$ of the  in the form of the multiplication by an matrix factor:

$$\begin{aligned} P^{(x)}(x,k)=\textrm{e}^{-\textrm{i}kx\sigma _3}P_0^{(x)}(k)\textrm{e}^{\textrm{i}kx\sigma _3} \end{aligned}$$

with

$$\begin{aligned} P_0^{(x)}(k)={\left\{ \begin{array}{ll} \begin{pmatrix} 1& -\frac{B(k)}{a^*(k)d^*(k)}\\ 0& 1 \end{pmatrix},\quad k\in \mathbb {C}^+\setminus \{|k|<\epsilon \}\\ \begin{pmatrix} 1& -\frac{\tilde{B}(k)}{\tilde{a}^*(k)\tilde{d}^*(k)}\\ 0& 1 \end{pmatrix},\quad k\in \mathbb {C}^+\cap \{|k|<\epsilon \}\\ \begin{pmatrix} 1& 0\\ \frac{B^*(k)}{a(k)d(k)}& 1 \end{pmatrix},\quad k\in \mathbb {C}^-\setminus \{|k|<\epsilon \},\\ \begin{pmatrix} 1& 0\\ \frac{\tilde{B}^*(k)}{\tilde{a}(k)\tilde{d}(k)}& 1 \end{pmatrix},\quad k\in \mathbb {C}^-\cap \{|k|<\epsilon \}. \end{array}\right. } \end{aligned}$$

Thus, the problem reduces to show that ${ N}^{(x)}(x,k)$ defined by

9.12 $$\begin{aligned} { N}^{(x)}(x,k)=\check{ M}^{(xt)}(x,0,k)P^{(x)}(x,k) \end{aligned}$$

satisfies the Riemann–Hilbert problem .

- The jump conditions match by construction.
- Notice that $-\frac{B(k)}{a^*(k)d^*(k)}\textrm{e}^{-2\textrm{i}k x}=O\left( \frac{\textrm{e}^{-2\textrm{i}k x}}{k}\right)$. In particular, we have $-\frac{B(k)}{a^*(k)d^*(k)}\textrm{e}^{-2\textrm{i}k x}=O\left( \frac{1}{k}\right)$ for x≤0 in $\mathbb {C}^+$, and thus, the normalization condition is satisfied.
- Let us check that the residue conditions match. For $k\in \mathbb {C}^+$, we have
  9.13 $$\begin{aligned} { N}^{(xt)(1)}(x,k)&=\check{M}^{(xt)(1)}(x,0,k),\end{aligned}$$
  9.14 $$\begin{aligned} { N}^{(xt)(2)}(x,k)&=-\frac{B(k)\textrm{e}^{-2\textrm{i}k x}}{a^*(k)d^*(k)}\check{M}^{(xt)(1)}(x,0,k)+\check{M}^{(xt)(2)}(x,0,k). \end{aligned}$$
  Taking into account the residue condition (5.16b) and the symmetries (2.38) and (2.45), the singularities of the second column at $k=-\eta _j$ cancel. On the other hand, at $k=- k_j$, the second column is singular due to the singularity of $\frac{1}{a^*(k)}$, and, using $d^*(-k_j)=b^*(- k_j)B(- k_j)$, the corresponding residue condition takes the form (4.29a). The singularities in $\mathbb {C}^-$ can be treated in a similar way.

Thus, by uniqueness of solution of RH problem , we conclude that $\check{ M}^{(xt)}(x,0,k)P^{(x)}(x,k)=\check{ M}^{(x)}(x,k)$.

Recall that $u_{0x}(x)$ is expressed in terms of the second coefficient in the expansion of $\check{ M}^{(x)}(x,k)$ near k=∞. In this regard, notice that $-\frac{B(k)}{a^*(k)d^*(k)}\textrm{e}^{-2\textrm{i}k x}$ decays exponentially fast for x<0 in $\mathbb {C}^+$, which implies that

$$ {u}_{x}(x,0)={u}_{0x}(x). $$

**2.** Introduce

$$ P^{(t)}_0(k)={\left\{ \begin{array}{ll} \begin{pmatrix} \frac{A(k)}{d^*(k)}& 0\\ b^*(k)& \frac{d^*(k)}{A(k)} \end{pmatrix},\quad k\in \mathbb {C}^+\cap \{|k|>\epsilon \},\\ \begin{pmatrix} \frac{\tilde{A}(k)}{\tilde{d}^*(k)}& 0\\ \tilde{b}^*(k)& \frac{\tilde{d}^*(k)}{\tilde{A}(k)} \end{pmatrix},\quad k\in \mathbb {C}^+\cap \{|k|<\epsilon \},\\ \begin{pmatrix} \frac{d(k)}{A^*(k)}& -b(k)\\ 0& \frac{A^*(k)}{d(k)} \end{pmatrix},\quad k\in \mathbb {C}^-\cap \{|k|>\epsilon \},\\ \begin{pmatrix} \frac{\tilde{d}(k)}{\tilde{A}^*(k)}& -\tilde{b}(k)\\ 0& \frac{\tilde{A}^*(k)}{\tilde{d}(k)} \end{pmatrix},\quad k\in \mathbb {C}^-\cap \{|k|<\epsilon \}, \end{array}\right. } $$

and define

$$ \hat{{ N}}^{(xt)}(t,k):=\check{ M}^{(xt)}(0,t,k)P^{(t)}(t,k) $$

with $$P^{(t)}(t,k)=\textrm{e}^{\frac{\textrm{i}t}{4 k}\sigma _3}P^{(t)}_0(k)\textrm{e}^{-\frac{\textrm{i}t}{4 k}\sigma _3}$$.

1. The jump condition for $\hat{{ N}}^{(xt)}(t,k)$ coincides with that for $\check{ M}^{(t)}(t,k)$ by construction.
2. Noticing that $\frac{A(k)}{d^*(k)}=1+O(\frac{1}{k})$ and $b^*(k)\textrm{e}^{-\frac{\textrm{i}t}{2 k}}=O(\frac{1}{k})$ in $\mathbb {C}^+$ for all t≥0, the normalization condition is satisfied.
3. Let us check that the residue conditions match. For $k\in \mathbb {C}^+$, we have
  9.15 $$\begin{aligned} \hat{{ N}}^{(xt)(1)}(t,k)&=\frac{A(k)}{d^*(k)}\check{M}^{(xt)(1)}(0,t,k)+b^*(k)\textrm{e}^{-\frac{\textrm{i}t}{2 k}}\check{M}^{(xt)(2)}(0,t,k),\end{aligned}$$
  9.16 $$\begin{aligned} \hat{{ N}}^{(xt)(2)}(t,k)&=\frac{d^*(k)}{A(k)}\check{M}^{(xt)(2)}(0,t,k). \end{aligned}$$
  Taking into account the residue condition (5.16b) and $A(-\eta _j)=-\frac{b^*(-\eta _j) B(-\eta _j)}{a^*(-\eta _j)}$ together with the symmetries (2.38) and (2.45), the singularities of the first column at $k=-\eta _j$ cancel. On the other hand, at $k= \mu _j$, the second column is singular due to the singularity of $\frac{1}{A(k)}$, and, using $d^*(\mu _j)=b^*(\mu _j)B(\mu _j)$, the corresponding residue condition takes the form (4.17b). The singularities in $\mathbb {C}^-$ can be treated in a similar way.

By the uniqueness of solution of the RH problem , it follows that $\hat{{ N}}^{(xt)}(t,k)=\check{ M}^{(t)}(t,k)$.

Since $v_{0t}(t)$ is expressed in terms of the first coefficient (via $\sin \tfrac{v_0}{2}$ and $\cos \tfrac{v_0}{2}$) in the expansion of $\check{ M}^{(t)}(0,t,k)$ near k=0, it is important to control $P^{(t)}(t,k)$ near this point. In this regard, notice that in $\mathbb {C}^+$ as k→0

$$ \frac{\tilde{A}(k)}{\tilde{d}^*(k)}=1+O(k) $$

and

$$ \tilde{b}^*(k)\textrm{e}^{2\textrm{i}k\left( -\frac{t}{4 k^2}\right) }=O(k\textrm{e}^{- \frac{\textrm{i}t}{2k}}). $$

In particular, $\tilde{b}^*(k)\textrm{e}^{-\frac{\textrm{i}t}{2 k}}$ decays exponentially fast as k→0 in $\mathbb {C}^+$ for all t>0. Therefore,

$$ P^{(t)}(t,k)= I+\begin{pmatrix} O(k)&  0\\ O(k\textrm{e}^{- \frac{\textrm{i}t}{2k}}) & O(k) \end{pmatrix}, \quad k\rightarrow 0, $$

and the expressions for $u_t(0,t)$, given in (6.26) remain unchanged.

□

#### Remark 9.4

The above zero-free assumptions are made in order to focus on the main ideas and avoid additional technical details; cf. Remark 8.9.

## Concluding remarks

Throughout this paper, we consider the generic case, in the sense that the initial data for Problem I and the initial and boundary data for Problem II are assumed to generate the corresponding spectral functions with finitely many zeros, all of which are simple. Under this assumption, the associated RH problems can be formulated by imposing residue conditions at the zeros of the spectral functions. The restriction to simple zeros is not essential, however. Indeed, the general case can be treated by replacing the residue conditions with a jump condition on a sufficiently large circle enclosing all the zeros; see [9, 11, 27].

## Data Availability

Data sharing is not applicable to this article as no datasets were generated or analyzed during the current study.

## References

[1] Frenkel, Y., Kontorova, T.: On the theory of plastic deformation and twinning. Zhurnal Eksperimentalnoi i Teoreticheskoi Fiziki **8**, 1340–1348 (1939)

[2] Scott, A.C., Chu, F.Y.F., McLaughlin, D.W.: The soliton: a new concept in applied science. Proc. IEEE **61**, 1443–1483 (1973)

[3] Ablowitz, M.J., Clarkson, P.A.: Solitons, Nonlinear Evolution Equations and Inverse Scattering. Cambridge University Press, Cambridge (1991)

[4] Faddeev, L.D., Takhtajan, L.A.: Hamiltonian Methods in the Theory of Solitons. Springer, Berlin (1987)

[5] Ablowitz, M.J., Kaup, D.J., Newell, A.C., Segur, H.: Method for solving the Sine-Gordon equation. Phys. Rev. Lett. **30**, 1262–1264 (1973)

[6] Kaup, D.J., Newell, A.C.: The Goursat and Cauchy problems for the Sine-Gordon equation. SIAM J. Appl. Math. **34**, 37–54 (1978)

[7] Fokas, A.S.: Integrable nonlinear evolution equations on the half-line. Commun. Math. Phys. **230**, 1–39 (2002)

[8] Fokas, A.S.: A Unified Approach to Boundary Value Problems. Society for Industrial and Applied Mathematics (2008)

[9] Boutet de Monvel, A., Shepelsky, D.: The Camassa–Holm equation on the half-line: a Riemann–Hilbert approach. J. Geom. Anal. **18**, 285–323 (2008)

[10] Fokas, A., Its, A.R., Sung, L.-Y.: The nonlinear Schrödinger equation on the half-line. Nonlinearity **18**, 1771 (2005)

[11] Boutet de Monvel, A., Fokas, A., Shepelsky, D.: Integrable nonlinear evolution equations on a finite interval. Commun. Math. Phys. **263**, 133–172 (2006)

[12] Karpenko, I., Shepelsky, D.: The modified Camassa–Holm equation on the half line: a Riemann–Hilbert approach. Stud. Appl. Math. **156**, e70254 (2026)

[13] Lenells, J., Fokas, A.S.: An integrable generalization of the nonlinear Schrödinger equation on the half-line and solitons. Inverse Prob. **25**, 115006 (2009)

[14] Fokas, A.S., Its, A.R.: An initial-boundary value problem for the sine-Gordon equation in laboratory coordinates. Theor. Math. Phys. **92**, 778–782 (1992)

[15] Huang, L., Lenells, J.: Nonlinear Fourier transforms for the sine-Gordon equation in the quarter plane. J. Differ. Equ. **264**, 3445–3499 (2018)

[16] Fokas, A.S.: A unified transform method for solving linear and certain nonlinear PDEs. Proc. Roy. Soc. Lond. Ser. A Math. Phys. Eng. Sci. **453**, 61411–1443 (1997)

[17] Sakhnovich, A.L.: The Goursat problem for the sine-Gordon equation and the inverse spectral problem. Russian Math. (Iz. VUZ) **36**, 42–52 (1992)

[18] Sakhnovich, A.L.: Sine-Gordon equation in a semi-strip. Nonlinear Anal. Theory Methods Appl. **75**, 964–974 (2012)

[19] Vu, K.T.: Initial-boundary value problems for the sine-Gordon equation on the half-line. Inverse Prob. **21**, 1363–1380 (2005)

[20] Leon, J.: Solution of the Dirichlet boundary value problem for the Sine-Gordon equation (2002). arXiv preprint:0204042

[21] Zhou, X.: The Riemann–Hilbert problem and inverse scattering. SIAM J. Math. Anal. **20**, 62 (1989)

[22] Lenells, J.: Matrix Riemann–Hilbert problems with jumps across Carleson contours. Monatshefte für Mathematik **186**, 111–152 (2018) 10.1007/s00605-017-1019-0 PMC6560487 31258193

[23] Zhou, X.: The Riemann–Hilbert problem and inverse scattering. SIAM J. Math. Anal. **20**, 966–986 (1989)

[24] Lenells, J.: Nonlinear Fourier transforms and the mKdV equation in the quarter plane. Stud. Appl. Math. **129**, 347–371 (2012)

[25] Teschl, G.: Topics in Linear and Nonlinear Functional Analysis. American Mathematical Society (to appear)

[26] Zhou, X.: -Sobolev space bijectivity of the scattering and inverse scattering transforms. Commun. Pure Appl. Math. **51**, 697–855 (1998)

[27] Boutet de Monvel, A., Fokas, A., Shepelsky, D.: The mKdV equation on the half-line. J. Inst. Math. Jussieu **3**, 139–164 (2004)

